# New Analogs of the Compstatin Family of Clinical Complement Inhibitors with Low Picomolar Target Affinity

**DOI:** 10.1021/acs.jmedchem.6c00832

**Published:** 2026-05-01

**Authors:** Stephanie A. Vogt, Alexander J. Lander, Karl Herbine, Ekaterina Umnyakova, Jannes Felsch, Roman Aschwanden, Sarah E. Hughes, Oliver Schwardt, Markus A. Lill, Martin Smieško, John D. Lambris, Christina Lamers, Daniel Ricklin

**Affiliations:** † Department of Pharmaceutical Sciences, 27209 University of Basel, 4056 Basel, Switzerland; ‡ Department of Pathology & Laboratory Medicine, Perelman School of Medicine, 6572 University of Pennsylvania, 422 Curie Blvd, Philadelphia 19104, Pennsylvania, United States; § Institute for Drug Discovery, Faculty of Medicine, 70622 Leipzig University, Brüderstrasse 34, 04103 Leipzig, Germany

## Abstract

Compstatin-class macrocyclic peptides have emerged as therapeutic complement modulators, with a PEGylated compstatin derivative being approved and sequence-optimized analogs with enhanced PK/PD properties showing clinical promise. By extending structure–activity relationship studies of compstatin, we identified a modification (V3I) that enhances the target affinity up to 30-fold. Analog Cp01-V3I represents the most potent proteinogenic compstatin (*K*_D_ = 20 nM), opening paths toward recombinant applications. Introducing the V3I modification into late-generation compstatin analogs yielded a low-picomolar-affinity derivative (*K*_D_ = 0.08 nM), termed Cp60, featuring potent complement inhibition in vitro. Cryogenic electron microscopy of the C3bB-Cp60 complex at 2.88 Å resolution confirmed the structural basis for enhanced target affinity and provided mechanistic insights. Lastly, we demonstrate that Cp60s ultralong target residence time enables diagnostic applications for detecting complement opsonins on biosurfaces. Collectively, this work highlights the importance of rigorous optimization of de novo peptide inhibitors to improve PK/PD properties and enable novel applications.

## Graphical abstract

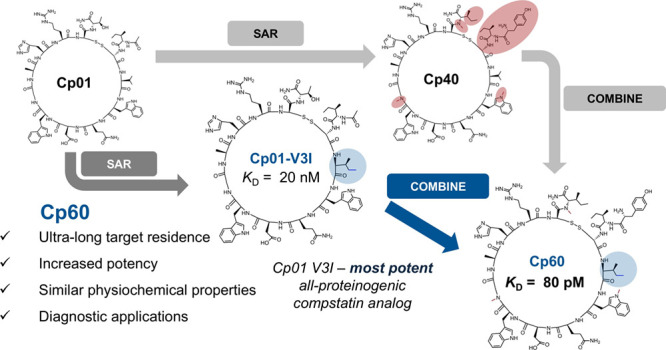

## Introduction

Compstatins, a class of macrocyclic peptides comprising 13–14 amino acids that bind and inhibit complement component C3, have emerged as a pillar in both experimental and therapeutic modulation of the complement system.^1^ As part of innate immunity, complement plays an essential role in host defense and homeostasis via recognition and clearance of foreign and apoptotic cells.^2^ However, ill-controlled complement activation is associated with a range of immune and inflammatory diseases. Therapeutic modulation of the complement system therefore represents a promising intervention strategy, and several complement-targeted drugs have been approved.^3^ Among those, pegcetacoplan (Empaveli/Aspaveli, Syfovre; Apellis), a PEGylated analogue of compstatin Cp05, remains the only C3 inhibitor in the clinic.^4,5^ Despite its approved use in multiple indications, including paroxysmal nocturnal hemoglobinuria (PNH), age-related macular degeneration, and C3 glomerulopathy (C3G), the rather short target residence time and, consequently, dependence on 40 kDa PEG as a pharmacokinetic (PK) modulator may impose limitations.^6^ A sequence-optimized candidate based on compstatin Cp40 (AMY-101; Amyndas) has since passed phase 2 clinical trials;^7^ its profoundly enhanced target affinity negates the need for PEGylation, with expected implications for safety and administration profiles.^8^ Further optimization of compstatin-family peptides, therefore, presents promising opportunities for the development of next-generation complement therapeutics.

Compstatins directly impair the activation of C3, a central hub of the complement cascade, which can provide advantages over other clinical inhibitors.^9^ While the cascade can be initiated by various means, such as recognition of antibody–antigen complexes (classical pathway; CP) or foreign carbohydrate signatures (lectin pathway; LP), it quickly converges at the formation of C3 convertase complexes on the activating cell surface.^10^ These convertases cleave the abundant plasma protein C3 into an opsonic fragment (C3b) and an anaphylatoxin fragment (C3a). C3b builds the platform for a concerted binding of two serine proteases, factor B (FB) and factor D (FD), generating the C3bBb complex that serves as the main C3 convertase and further amplifies the response via the alternative pathway (AP).^11^ Thereby, the system is able to rapidly opsonize surfaces that lack complement regulators, including bacterial or viral cells. Increasing C3b deposition enables the formation of C5 convertases that cleave the plasma protein C5 to release the pro-inflammatory anaphylatoxin C5a and induce the generation of membrane attack complexes (MAC) that lyse or damage susceptible cells.^12^ Compstatins were shown to tightly bind the macroglobulin (MG) core of C3 and C3b; as protein–protein-interaction (PPI) inhibitors of C3 binding to the convertase, they prevent cleavage and activation of C3.^9,13^ Consequently, compstatins broadly attenuate complement amplification and effector generation independent of the initiation pathway.^1^

Compstatin was originally identified from phage display library screening as a 13-amino-acid cyclic peptide featuring micromolar affinity for C3/C3b.^14,15^ Optimization efforts led to several generations of compstatin analogs with improved affinity and target residence, inhibitory efficacy, and PK properties (Figure 1). Initial improvements were achieved by replacing individual amino acids in the cyclic core,^16,17^ resulting in analog Cp01, which remained the most potent compstatin derivative solely composed of canonical amino acids.

**Figure 1:**
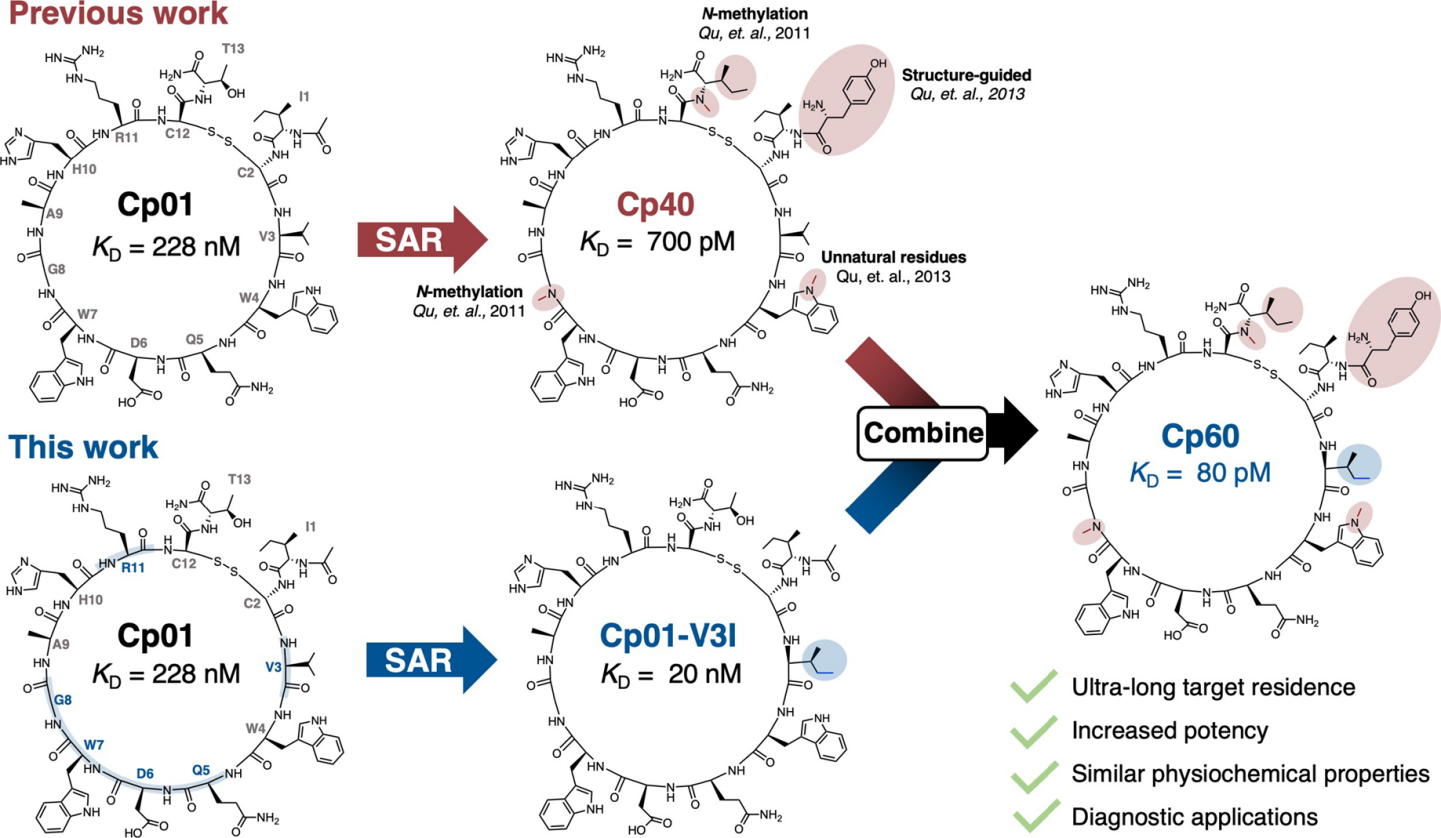
Overview of the Structure–Activity Relationship (SAR) Studies in the compstatin family of peptidic C3 inhibitors. Modifications in the development of Cp40, starting from Cp01, included amino-acid backbone methylation,^18^ inclusion of unnatural residues,^8^ and structure-guided N-terminal peptide extension.^8^ *K*_D_ values for the compstatin analogs binding to human C3b determined by SPR are indicated.

Indole-*N*-methylation of Trp4 marked a breakthrough that produced analog Cp05 with low-nanomolar target affinity.^19^ Cp05 was licensed to Apellis for clinical development and forms the active moiety of pegcetacoplan.^5,20^ Since the plasma half-life of compstatins is largely defined by their interaction with the abundant plasma protein C3 (1–2 g/L), the comparatively short target residence of Cp05 necessitated the bivalent conjugation to a central 40-kDa PEG unit to reduce elimination.^21^

Even if the clinical use of pegcetacoplan is generally considered safe,^21–23^ the bivalent format and high dose requirements for systemic use of the PEGylated drug (i.e., 1080 mg twice weekly in the case of PNH and C3G)^24^ may impose limitations for some indications. Enhancing the target residence therefore defined a major aim of subsequent development efforts guided by *N*-methylation screening and structure–activity relationship (SAR) studies.^8,18^ The resulting lead Cp40 contains 4 noncanonical residues, i.e., d-Tyr0, ^1Me^Trp4, and backbone *N*-methylated Gly8 (sarcosine; Sar8) and Ile13 (^N-Me^Ile13) (Figure 1). Owing to its improved target affinity/residence (C3b *K*_D_ = 700 pM), an extended half-life could be achieved in Cp40 without the need of PEGylation.^8^ The Cp40-based candidate AMY-101 has since been successfully evaluated in clinical trials for periodontitis and acute respiratory distress syndrome due to SARS-CoV-2 infection.^7,25,26^

Iterative optimization of the compstatin class yielded valuable drugs and candidates, generally emphasizing the potential of SAR studies to modulate target-binding properties. So far, optimization efforts mainly focused on extending *N*-terminal contacts or modifying key residues identified during compstatin’s early characterization. Although earlier alanine-scanning studies revealed that residues 3 and 5–8 are essential in the activity of compstatin,^17^ and have been subject to some investigation,^27^ these positions have not been examined at the same level of scrutiny but may bear potential for PK/PD enhancement, drug conjugation/targeting, or specificity/selectivity tuning.

In this work, which builds on over a decade of systematic research on lead peptide optimization through SAR and rational, structure-guided peptide modifications,^1^ we bridge the gap in the SAR of compstatin by focusing on positions such as Val3 or the peptide segment from Gln5 to Gly/Sar8, the latter of which was early recognized as critical for target binding and stabilization of a beta-turn motif (Figure 1).^17,28^ Among those, we found a V3I modification of particular interest, as it boosted target affinity for current lead analogs, yielding compstatin Cp60 with picomolar target affinity (C3b *K*_D_ = 80 pM). The profound impact on target residence enabled new applications of high-affinity compstatins for the detection of C3-opsonized particles and cell surfaces. Collectively, we demonstrate the critical importance of careful and rigorous optimization of clinical peptides, with the new analogs paving the way for next-generation complement inhibitors and new biochemical applications.

## Results

### Extended SAR Analysis of Compstatin Reveals Analogs with 12-Fold Improved Potency

For our extended SAR study, we use analog Cp01 rather than Cp40 or other high-affinity derivatives as reference compound. From a technical perspective, the rapid dissociation rate of Cp01 facilitates kinetic screening by surface plasmon resonance (SPR), and its composition of only proteinogenic amino acids simplifies synthesis. Moreover, the identification of all-canonical derivatives with improved target binding properties may beneficially impact drug production costs and enable the design of peptide–protein fusion strategies. Finally, an experimentally achieved cocrystal structure of Cp01 in complex with the target fragment C3c is available, which has been used to guide the SAR study (Figure 2A).^9^

**Figure 2:**
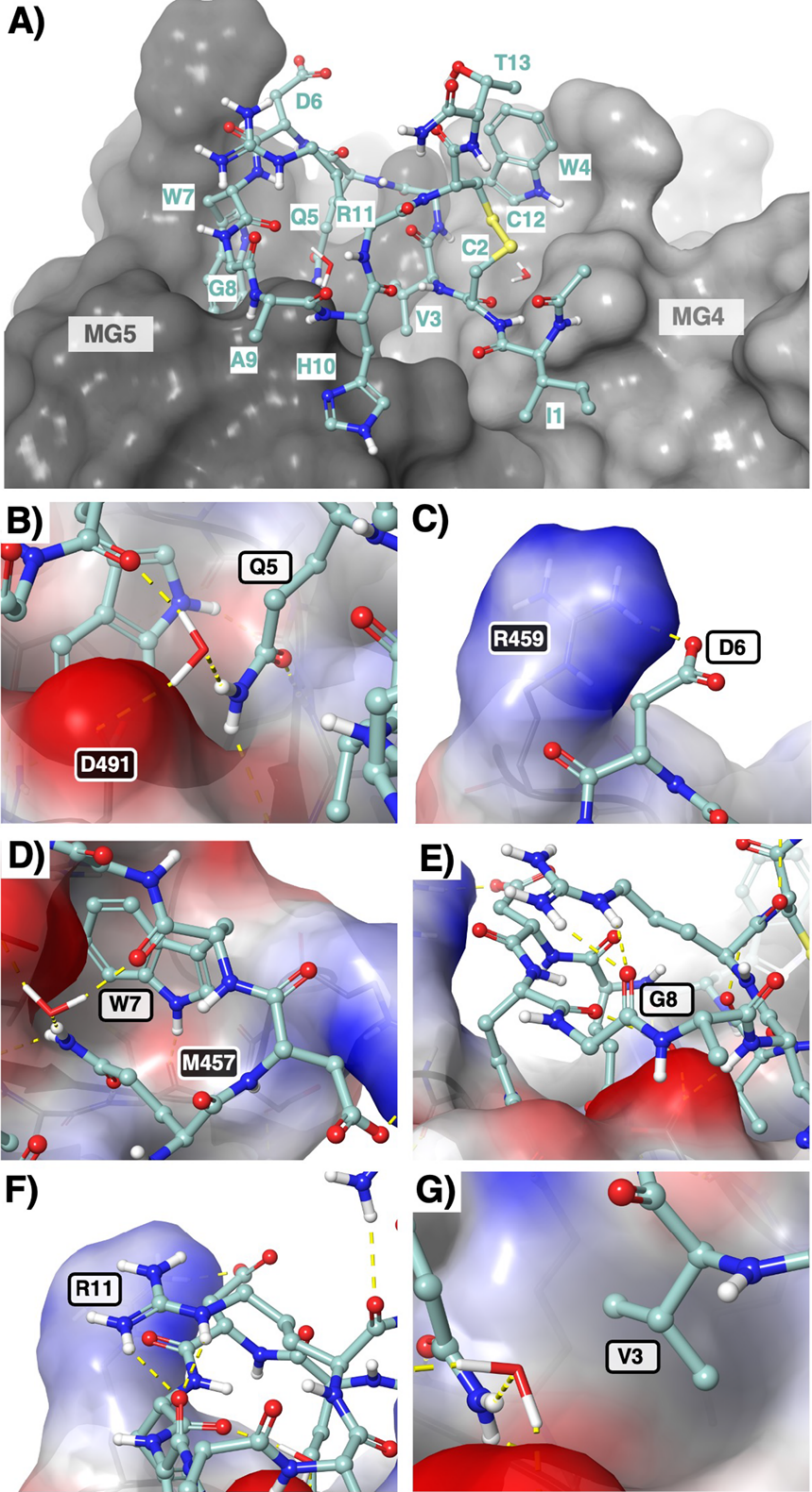
Drug–target interaction profile of compstatin Cp01. (A) Full view of Cp01 binding on the surface of C3c. (B–G) Close-up views of peptide–protein interactions investigated in this work. Structures derived from deposited coordinates (PDB: 2QKI)^9^ and figures constructed in Maestro. Cp01 is shown in ball-and-stick representation and the target C3c as solvent-accessible surface colored in gray (A) or according to electrostatic potential (B–G).

We first explored amino-acid substitutions at positions 5–8, as solution-state NMR experiments during early compstatin development suggested that these residues were responsible for stabilization of a β-turn motif considered important for target binding.^17,28^ To probe the roles of these amino-acid side chains, we prepared cyclic peptide analogs with single substitutions by solid-phase peptide synthesis (SPPS) and tested their binding to human C3b by SPR. All analogs were initially screened for binding at fixed concentrations (2 and 1 μM, Figure S1). Analogs that showed noticeable binding were further analyzed by serial 2-fold concentration series to assess target binding kinetics (Table 1 and Figures S2–S15).

**Table 1:** Structure–Activity Relationships of Cp01 Showing Kinetic Constants for Binding to Human C3b by SPR

		**affinity hC3b** ^a^		
**Cp01 residue**	**substitution**	***K***_**D**_ (nM)	***k***_**a**_ **(10** ^**5**^ **M** ^**–1**^ **s** ^****–**1**^ **)**	***k***_**d**_ **(10** ^****–**2**^ **s** ^****–**1**^ **)**
unmodified Cp01		228(31)	3.5(0.5)	7.8(0.7)
Val3	Ala	N.B.^h^		
	Leu	552(139)	2.0(0.5)	11(1)
	Aib^b^	N.B.		
	Tbg^c^	103(16)	5.7(0.8)	7.9(3.7)
	Trp	N.B.		
	Phe	N.B.		
	Tyr	N.B.		
	Ile	19.8(1.7)	8.8(0.1)	1.7(0.1)
	Abu^d^	7753(2595)	0.4(0.2)	26(5)
	Nva^e^	1663(503)	1.5(0.5)	24(3)
Gln5	Lys	N.B.		
	Orn	N.B.		
Asp6	Ser	609(104)	3.1(0.9)	18(3)
	Asn	219(47)	5.4(1.5)	11(1)
	Glu	528(206)	3.8(1.6)	18(2)
	Asu^f^	2077(637)	1.2(0.5)	23(4)
Trp7	Bta^g^	W.B.^i^		
Gly8	Ala	N.B.		
	d-Ala	W.B.		
Arg11	Lys	502(57)	3.0(0.8)	15(2)
	Ser	529(135)	3.3(1.2)	16(3)

a Kinetic parameters are reported as the average of 3 independent experiments fitted with 1:1 Langmuir binding models; standard deviations are included in brackets (SPR sensorgrams shown in Figures S2–S15). Noncanonical amino acids.

b 2-Aminoisobutyric acid.

c α-*tert*-Butyl-glycine.

d α-Aminobutyric acid.

e Norvaline.

f 2-Aminosuberic acid.

g (3-Benzothienyl)­alanine.

h N.B.: no binding detected at concentrations up to 2 μM.

i W.B.: kinetic parameters could not be determined due to weak binding (*K*_D_ > 16 μM).

Gln5 has a central role in Cp01’s target-binding interface as it engages directly in H-bonding interactions with Asp491 and backbone atoms of C3c, and can also coordinate a bridging water molecule (Figure 2B). Its substitution by Ala was previously shown to result in a profound loss of target binding.^17^ Based on this insight, we substituted Gln5 with either Lys or ornithine (Orn), the latter of which contains a side chain of equal length to that of Gln. Both analogs showed no detectable target binding, despite the potential for electrostatic interactions with Asp491. The replacement of the amide side chain with a charged primary amine may disrupt peptide–target interactions by destabilizing the crystal water, introducing a charge and H-bond donor excess in the proximity of the lipophilic cleft between MG4 and MG5, or destabilizing the β-turn motif. Additionally, the loss of an H-bond to the backbone-NH of Met457 might contribute to the effect.^16,28^

Although Asp6 appears oriented toward the solvent in the Cp01-C3c structure, its proximal location to Arg459 indicates a potential for electrostatic interactions (Figure 2C).^13^ Increasing the length of the aliphatic side chain carrying the carboxylate by substituting Asp with Glu or 2-aminosuberic acid (Asu), resulted in 2- and 10-fold affinity drops, respectively (Table 1). Replacing this acidic residue with uncharged Ser also resulted in a weaker target affinity (Table 1). To further explore the importance of a negative charge at position 6 for target binding, we substituted Asp with Asn as an isosteric, neutral analog. Surprisingly, negligible changes in affinity were observed, indicating that the presence of a negative charge at position 6 is not essential for target binding; rather, the interplay between side-chain length and a potential H-bond acceptor (HBA) in close proximity of Arg459 appears to be more important (Table 1). Thereby, these findings support previous hypotheses about a low relative impact of solvent-exposed salt-bridges.^29^ To investigate whether this insight regarding charge-independence extends to Arg11, which appears similarly solvent-oriented and is not directly involved in target interactions in the crystal structure (Figure 2F), this residue was substituted by either Ser or Lys. In both analogs, a ∼2-fold weaker affinity was observed, which could be attributed to faster dissociation rates (Table 1). Therefore, the Arg side chain plays a role in stabilization of the peptide–protein complex, likely through engaging in intramolecular H-bond interactions with macrocycle backbone carbonyls (Figure 2F).^13,28^

Substitution of Trp7 by Ala has earlier been found to weaken, but not abolish, affinity,^17^ with the indole-NH of Trp7 acting as H-bond donor (HBD) for the backbone carbonyl of Met457 (Figure 2D).^9,19^ Moreover, indole *N*-methylation at this position had resulted in a complete loss of target binding.^19^ However, it remained unclear whether this effect was caused by the loss of HBD capacity or by steric restrictions for accommodating the extra methyl group. We therefore prepared Cp01 analogs carrying 3-benzothienyl alanine (Bta) as a Trp bioisostere that lacks conventional HBD capacity without the additional steric constraints of the methyl group.^30^ Although the sulfur atom of Bta is capable of forming chalcogen S···O bonds with carbonyl oxygens, this would require slightly different directionality (*a*_C–S···O_ ∼ 80°) and distance (*d*_S···O_ ∼ 3.2 Å) than the conventional indole N–H donor of Trp (*a*_C–N···O_ = ∼120°, *d*_N**···**O_ = ∼2.8 Å) (Figures S16 and S17). The W7Bta analog showed strongly impaired affinity for C3b (Table 1), thereby confirming the hypothesis that the HBD property of Trp at position 7 is critical for target binding with stringent requirements for molecular recognition.^19^ Gly8 is in close proximity to the target interface and likely plays the role of a conformationally unrestricted hinge that maintains the cyclic peptide in a bioactive conformation (Figure 2E).^16,17,28^ Substituting Gly with Ala, in either chirality, diminished target binding; this may be ascribed to unfavorable steric hindrance, with either the carbonyl group of Gly489 and Trp7 (l-Ala) or the side chain of Asp 491 (d-Ala). These observations suggest that there is little to no room for side-chain derivatization at position 8 (Table 1).

While Trp4 had early been recognized as a key residue for lead development and was substituted with ^1Me^Trp in Cp05 and all subsequent analogs, the adjacent Val3 remained unchanged throughout the evolution of the compstatin family.^1^ Earlier SAR studies and patent applications identified Val3 as an important interaction determinant and suggested substitutions with hydrophobic residues.^17,27,28^ However, the observed affinity loss upon replacement of Val3 with structurally related Ala or Leu in such studies favored an interpretation that position 3 would not provide sufficient room for optimization. Structural models confirmed that the hydrophobic side chain of Val3 engages in van der Waals interaction within a hydrophobic cleft between the MG4 and MG5 domains of C3 (Figure 2A,G),^9,13^ supporting the view that this residue was already optimally positioned. Advances in SAR methods prompted us to re-examine this position and consider alternative hydrophobic or noncanonical residues (Table 1 and Figure 3A).

**Figure 3:**
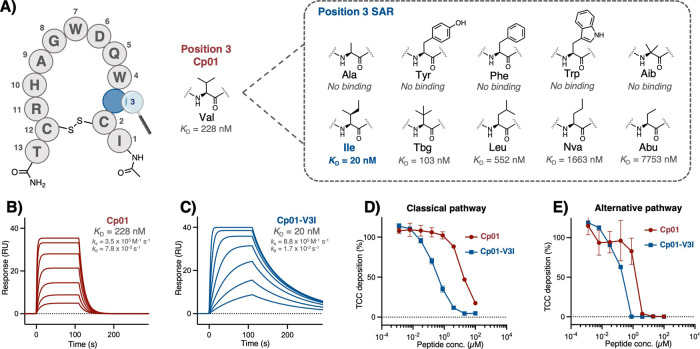
SAR studies focused at position 3 of Cp01. (A) Schematic overview of Cp01 and amino acid substitutions of Val3, with their impact on target interaction indicated by measured affinities (*K*_D_) of the analogs for binding to C3b by SPR, (B and C) SPR analysis of Cp01 (B) and Cp01 V3I (C) binding to C3b, with fitted data curves (1:1 binding) illustrating the improved dissociation rate of Cp01-V3I, (D and E) inhibition curves from in vitro complement activity ELISA assays in 1% diluted normal human serum for classical pathway (D) and alternative pathway (E), showing the improved inhibition by Cp01-V3I.

We prepared and analyzed 10 analogs of Cp01, including both canonical and noncanonical amino acids at position 3, focusing on those bearing hydrophobic side chains. Amino acids with larger aromatic side chains, i.e., Tyr, Phe, and Trp, were all detrimental to binding; similarly, the disubstituted C-α atom in 2-aminoisobutyric acid (Aib) also led to an affinity loss (Table 1 and Figure 3A). In contrast, and despite differing by only one additional methyl group, substitution of Val3 with Ile resulted in improved binding affinity when compared to Cp01 (*K*_D_ = 19.8 vs 228 nM) (Table 1 and Figure 3B,C). Except for *tert-*butyl glycine (Tbg, *K*_D_ = 103 nM), aliphatic side chains with subtle changes to the hydrocarbon configuration, including Leu, norvaline (Nva), and aminobutyric acid (Abu), all resulted in weaker target affinity when compared to the parental Val3 (Table 1 and Figure 3A). Thus, the addition of a single δ-methyl group with proper chirality, changing Val to Ile, appears to result in highly specific target contacts. With the Cp01-V3I analog at hand, we tested whether the improved binding affinity translated to functional efficacy. Both Cp01 and Cp01-V3I were tested for the ability to impair complement activation in diluted human serum via the classical (CP) or alternative (AP) pathway.^31^ In line with target interaction analyses, Cp01-V3I demonstrated markedly improved potency for both CP (IC_50_ = 0.43 vs 20 μM) and AP (IC_50_ = 0.20 vs 1.4 μM) inhibition when compared to Cp01 (Figure 3D,E). To our knowledge, Cp01-V3I represents the most potent compstatin analog comprised entirely of proteinogenic amino acids reported to date.

### SAR-Based Enhancements in Cp01 Are Directly Transferrable to Newer-Generation Clinical Compstatin Analogs

Intrigued by the strong impact of a Val-to-Ile substitution at position 3 on the target affinity and functional efficacy in the case of Cp01, we aimed to combine this substitution with modifications introduced into later generations of the compstatin family. Cp05, the active peptide moiety in pegcetacoplan, contains indole *N*-methylation at Trp4.^19^ Combining the V3I and W4^1Me^W modifications indeed resulted in synergistic effects, with 150-fold improvement in affinity (*K*_D_ = 1.5 vs 228 nM) and 40-fold slower dissociation rate (*k*_d_ = 0.002 vs 0.078 s^–1^), when compared with Cp01 (Table 2, Figures S18, and S19). The latest-generation compstatin Cp40 includes 4 noncanonical modifications, i.e., acetyl0d-Tyr, Trp4^1Me^Trp, Gly8Sar, and Thr13^N‑Me^Ile (Figure 4A).^8,18^ Combined, these modifications result in a potent analog with high affinity (*K*_D_ = 0.7 nM) and long target residence (*k*_d_ = 0.1 × 10^–2^ s^–1^) that defines the clinical candidate AMY-101 (Table 2). Transferring the V3I modification to the highly optimized Cp40 scaffold resulted in a compstatin analog with even further improved affinity (*K*_D_ = 80 pM) (Table 2, Figures S20, and S21). The peptide, termed Cp60, showed an exceptionally slow dissociation rate (*k*_d_ = 0.01 × 10^–2^ s^–1^), reflecting a target residence exceeding 100 min that is rarely observed in peptides targeting a PPI interface and more akin to antibody interactions (Figure 4B).

**Figure 4:**
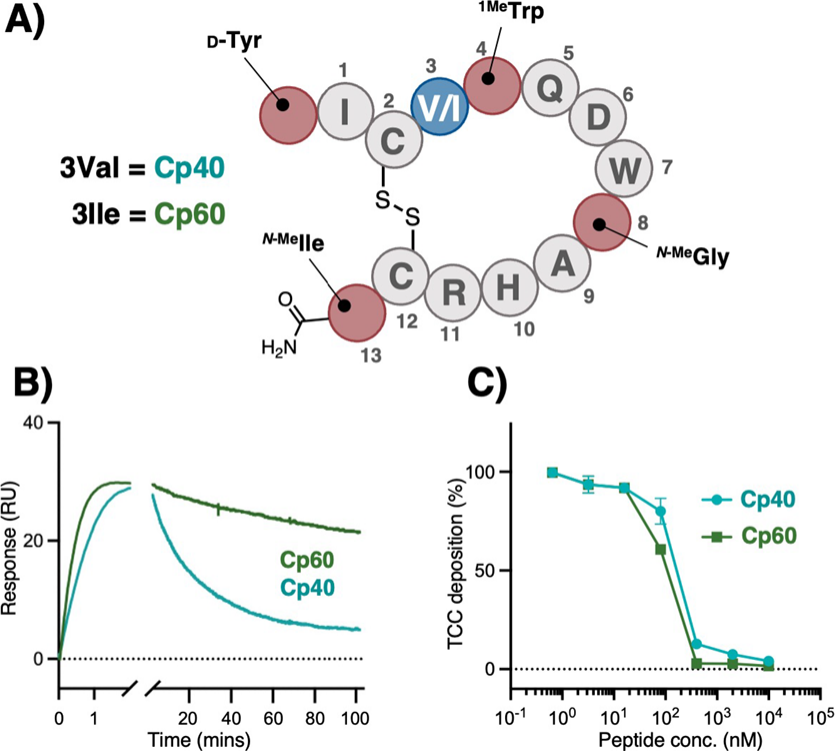
Transfer of Val-to-Ile substitution to Cp40 produces analog Cp60. (A) Schematic overview of the structure of Cp40 (3Val) and Cp60 (3Ile). (B) SPR analysis of Cp40 and Cp60 (both at 40 nM) binding to C3b, illustrating the improved target residence (>100 min) of Cp60. (C) Inhibition curves from in vitro complement activity assay (CP ELISA) in 5% human serum, showing the superior inhibitory potency of Cp60 when compared to Cp40.

**Table 2:** Effect of Position-3 Analogs on the Binding of Different Compstatin Generations to Human C3b, Measured by SPR

		**affinity hC3b** ^a^		
**compstatin scaffold**	**residue** (Pos. 3)	***K***_**D**_ (nM)	***k***_**a**_ **(10** ^**5**^ **M** ^****–**1**^ **s** ^****–**1**^ **)**	***k***_**d**_ **(10** ^****–**2**^ **s** ^****–**1**^ **)**
Cp01	Val	228(31)	3.5(0.5)	7.8(0.7)
	Ile	19.8(1.7)	8.8(0.1)	1.7(0.1)
	aIle^b^	545	n.d^d^	n.d
	Dea^c^	466	n.d	n.d
Cp05	Val	46.8(5.0)	3.0(0.4)	1.4(0.2)
	Ile	1.5(0.0)	11.4(1.2)	0.2(0.0)
Cp40	Val	0.7(0.0)	17.6(0.3)	0.1(0.0)
	Ile^e^	0.08(0.2)	17.6(1.4)	0.01(0.0)
	aIle	15.6(5.0)	4.6(0.7)	0.7(0.3)
	Dea	13.6(2.9)	4.3(0.7)	0.6(0.1)

a Kinetic parameters are reported as the average of 3 independent experiments fitted with 1:1 Langmuir binding models, with standard deviations included in brackets (SPR sensorgrams shown in Supporting Information).

b Allo-isoleucine.

c β,β-Diethylalanine,

d n.d.: not determined; reported *K*_D_ determined as steady state affinity.

e Analog Cp40-V3I is also referred to as Cp60.

Next, we evaluated the in vitro complement inhibition potency of Cp60 using the CP ELISA as described above. Despite the clear advantage regarding target binding, Cp60 did not show improved CP inhibition compared with Cp40 when assessed in 1% human serum (Figure S22). Suspecting that the serum dilution in the standard assay may limit the dynamic range and resolution of the two analogs regarding IC_50_ values, we increased the serum concentration to 5%. Indeed, under these conditions, superior CP inhibition potency was observed for Cp60 (IC_50_ = 97 nM) when compared to Cp40 (IC_50_ = 158 nM) (Figure 4C). While technical limitations prevent a use of higher serum levels, the increase from 1 to 5% did improve the assay’s dynamic range.

Nevertheless, there remains considerable divergence between the detected enhancements in binding affinity (>8-fold) and inhibitory potency (<2-fold) when comparing Cp40 and Cp60. Such nonlinear relationship between affinity and apparent potency under in vitro conditions has earlier been described,^8,16,18,19,32^ and appears to have become more prominent during compstatin’s development. To corroborate these observations experimentally under controlled conditions, we determined apparent potency (IC_50_; CP ELISA) and C3b affinity (*K*_D_; SPR) for a panel of 10 compstatin analogs within a broad affinity range (*K*_D_ 0.08–552 nM). Indeed, a correlation plot confirms the nonlinear relationship between these values, with a 6900-fold *K*_D_ range yet only 240-fold variation of IC_50_ (Figure S23). Therefore, alternative assay formats may be used during further studies to explore whether the activity difference between Cp40 and Cp60 becomes even more prominent at physiological serum concentrations.

Notwithstanding dynamic range limitations of in vitro assays, earlier in vivo studies reported notable correlations between target affinity, and particularly, target residence time, and PK profiles of compstatin analogs.^8^ Given its slow dissociation rate constant (*k*_d_ = 0.0001 s^–1^) and long target residence (τ > 100 min), it could therefore be speculated that Cp60 may feature an improved terminal half-life when compared to previous compstatin derivatives. While the narrow species specificity of compstatins for human and nonhuman primate (NHP) complement restricts full in vivo PK profiling of Cp60, a limited single-dose study of a lysine-modified Cp60 derivative (Cp60-KK, 2 mg/kg) in cynomolgus monkeys could be conducted. Drug monitoring of Cp60-KK over a period of 120 h revealed high systemic drug exposure (AUC_0–120h_ = 230–400 μM h) and a slow elimination profile (*t*_1/2_ = 31 and 41 h, *n* = 2) in line with high-affinity C3 binding in vivo (Table S1 and Figure S24). However, considering the limited number of animals and lack of direct comparison with other analogs such as Cp40-KK,^32^ further studies are required to define the full PK profile should Cp60 enter preclinical development. Nevertheless, Cp60 features promising molecular and functional properties that may guide the development of next-generation compstatin analogs for clinical or biomedical applications.

### Alkyl Extension at Position 3 Addresses the Specific Target Pocket

The evolution of the compstatin class serves as an intriguing example of how subtle structural modifications of a lead peptide can result in profoundly enhanced target affinity. Indeed, indole *N-*methylation of Trp4 by itself resulted in 5-fold improved binding (Cp01 → Cp05, Table 2),^19^ which was later attributed to shielding of a structural water at the target interface by the additional methyl group.^13^ Meanwhile, earlier work has shown that backbone *N*-methylation at Gly8 and Ile13 benefit target affinity by stabilizing a bioactive conformation in solution.^18^ In analogy to these affinity-enhancing modifications, the Val-to-Ile substitution reported here also entails the addition of a single methyl group, in this case at the γ-position of Val3 (new δ-methyl). Of note, this modification produces a diastereomeric side chain in 2*S,*3*S* configuration (Figure 5A).

**Figure 5:**
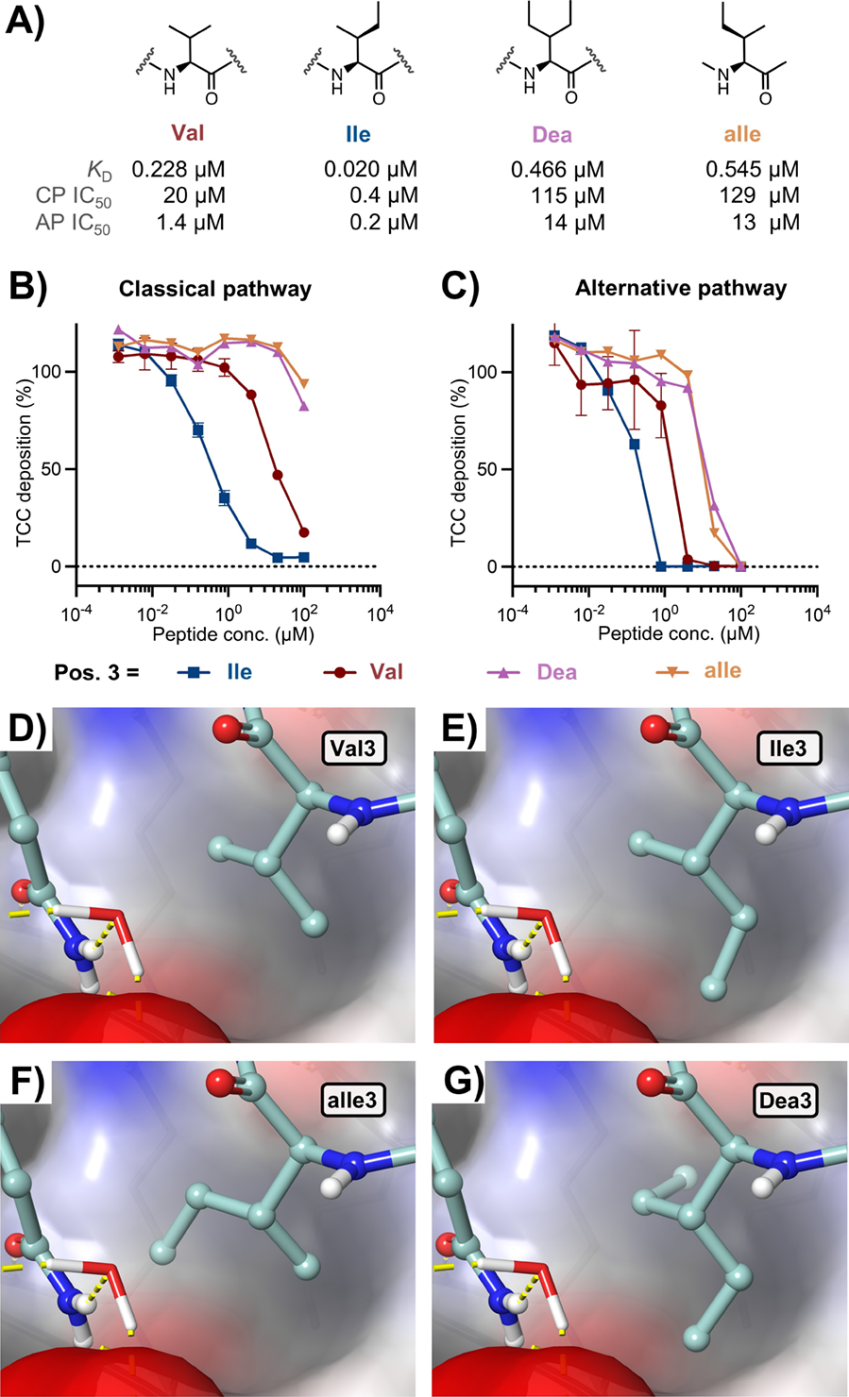
Characterization of drug–target interaction determinants at position 3. (A) Structures of amino acid analogs screened at position 3, indicating inclusion in Cp01: C3b *K*_D_, determined by SPR, CP and AP IC_50_, determined by ELISA in 1% diluted human serum, (B, C) Inhibition curves of the Cp01 position 3 analogs for complement activity ELISA assays, (D–G) in silico structural models of Cp01 position 3 analogs, highlighting specific accommodation of the additional methyl group of Ile3.

To gain further insight into the regio- and stereospecificity of the added methyl group regarding target binding, we prepared analogs of Cp01 and Cp40 that each carried 2 different amino-acid analogs at position 3. Whereas allo-isoleucine (aIle), the 2*S*,3*R*-diastereomer of Ile, reflects a single methyl addition at distinct stereoconfiguration, β,β-diethylalanine (Dea) represents the addition of methyl groups to both branches of Val (Figure 5A). In both the Cp01 and Cp40 scaffolds, the aIle3 and Dea3 analogs resulted in weaker target affinity and complement inhibition potency when compared to Val3 (Table 2, Figures 5B,C, and S25–S30). Therefore, these results indicate that the additional δ-methyl group in the Val-to-Ile substitution addresses a distinct pocket in the target interface and that both stereospecificity and regiospecificity of the added methyl group are critical. The bifurcation at C-β naturally restricts accessible rotamers of Val, Ile, and related residues for favorable interaction with the pocket. The most likely Ile rotamer (i.e., *gauche*) orients the additional δ-methyl group toward Leu454 without introducing steric clashes (Figures 5D,E, S31, and S32). Mutations to aIle and Dea are sterically more demanding (Figure 5F,G) and do not have the same rotamer preference as Ile. Even if Abu and Nva are sterically less demanding, both analogs lack bifurcation at the β-carbon that could interact with Met346 and Arg456. Abu and Nva also feature more accessible conformations, which may increase the entropic penalty upon binding, as indicated by reduced target association rates (Table 1).

Together, in silico modeling and focused SAR strongly imply a critical role of the alkyl extension at Val3 (Val3Ile) in addressing a distinct hydrophobic pocket within the target interface. As the predictive value of our in silico findings relies on the assumption that Cp60 shares a conserved binding mode with earlier-generation compstatins such as Cp01 and Cp40,^9,13^ we aimed at corroborating the model by determining an experimental structure of target-bound Cp60. Previously, X-ray crystallography was used to resolve the structure of Cp01 bound to fragment C3c,^9^ and Cp40 bound to C3b.^13^ However, it is known that compstatins also bind to AP C3 convertase complexes, defining a critical aspect of their mechanism of action.^13^ As the instability of the active convertase C3bBb (*t*_1/2_ ≈ 90 s) restricts experimental structure determination,^33^ we employed cryogenic electron microscopy (cryo-EM) to determine the structure of a Cp60 derivative, Cp60-KK, bound to the AP pro-convertase complex, C3bB. Plasma-purified C3b and FB were premixed with Cp60-KK to assemble the C3bB-Cp60 complex. Single-particle cryo-EM analysis of the drug–target complex (Figures S33 and S34) revealed two closely related conformations of the thioester-containing domain (TED). Although both reconstructions exhibited well-resolved density, only one map was used for structural interpretation, which resulted in a detailed structural model at 2.88 Å resolution (Figure 6). The observed differences in TED conformations are outside the scope of the current study but likely reflect intrinsic flexibility within TED.

**Figure 6:**
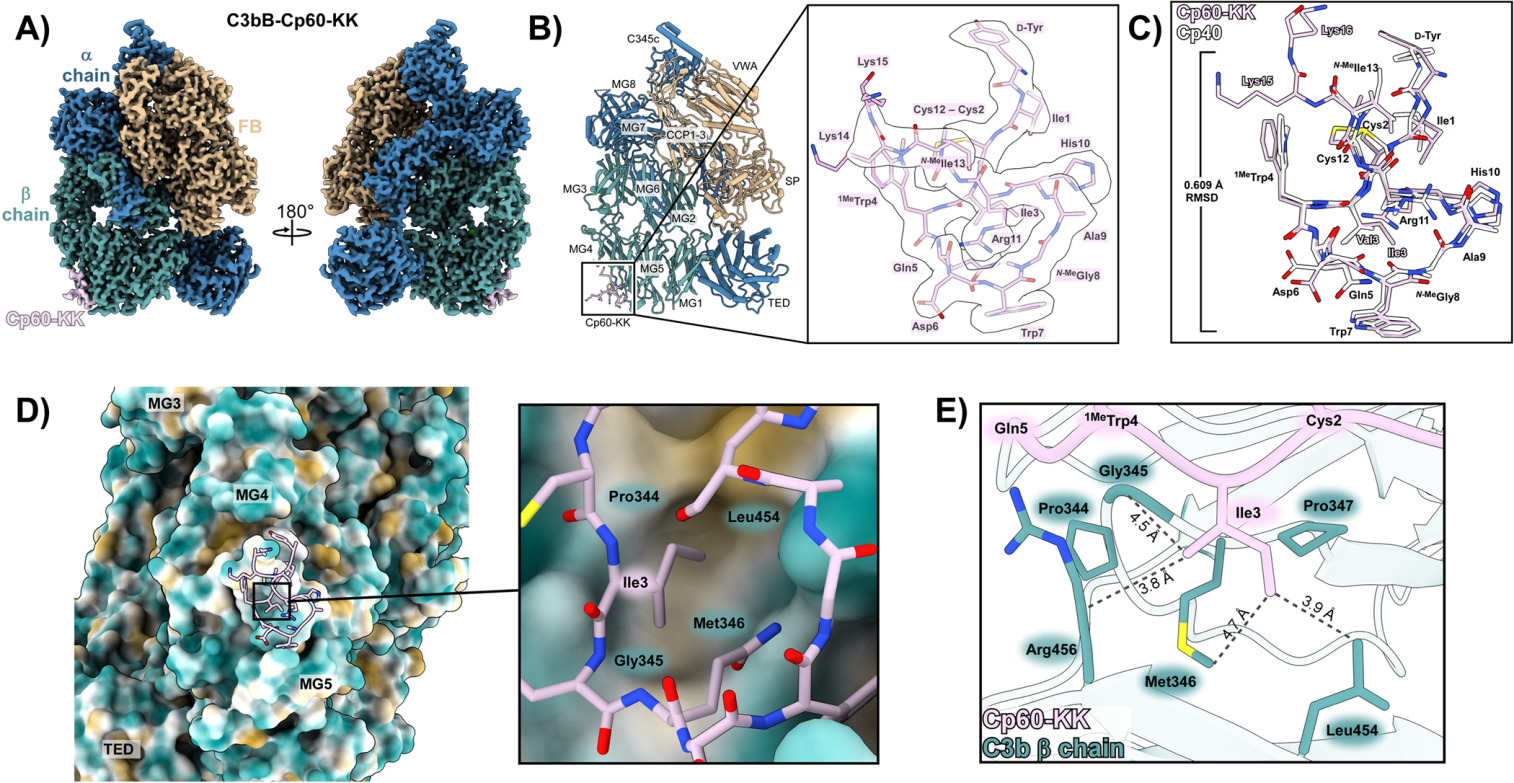
Structure of compstatin analog Cp60-KK in complex with the pro-convertase C3bB. (A) Cryo-EM density for the C3bB:Cp60-KK structure. The C3b α- and β-chains, factor B (FB), and the cyclic peptide Cp60-KK are colored steel blue, cadet blue, tan, and thistle, respectively. (B) Structural model of Cp60-KK bound to C3bB, with C3b and FB domains indicated. Inset: close-up view of Cp60-KK (pink) with residues indicated and cryo-EM density shown as a transparent surface. (C) Structural comparison between Cp60-KK (pink) and Cp40 (white). The Cp40 model was obtained from PDB ID: 7BAG,^13^ and the structures were aligned based on their Cα backbones. Amino-acid substitutions are labeled and colored according to their respective structures. The Cα RMSD between Cp60-KK and Cp40 (0.609 Å) indicates a high degree of structural similarity. (D) Binding interface between C3bB and Cp60-KK, with structures of C3b and Cp60-KK colored based on hydrophobicity (mlp command, ChimeraX)^34^ with cyan, white, and gold indicating hydrophilic, neutral, and hydrophobic residues, respectively. Inset: Close-up view of Ile3 shows that the side chain sits inside a hydrophobic pocket coordinated by Pro344, Met346, and Leu454 on the C3b β-chain. (E) Close-up view of Ile3 and adjacent residues within Cp60-KK and C3b. The introduction of the δ-methyl group from the Val-to-Ile substitution enables additional van der Waals (vdW) interactions with Met346 and Leu454. Alternative rotamers of Ile3 would likely cause steric clashes with Gln5 of Cp60-KK or lead to a loss of favorable vdW contacts within the hydrophobic pocket; the modeled rotamer thus represents the most favorable conformer for Ile3, rationalizing the affinity gain observed for the Val3 to Ile3 substitution.

Clear density was observed for the α- and β-chains of C3b and complex-bound FB (Figure 6A). Moreover, the map revealed a clear density of Cp60-KK bound within the cleft between the MG4 and MG5 domains of the C3b β-chain, consistent with the binding mode reported in previous X-ray crystal structures of Cp01 and Cp40 (Figure 6A).^9,13^ Alongside confirming a conserved target interaction profile across compstatin analogs, this study also provides the first structural evidence of compstatins binding to an AP C3 convertase complex, complementing previous mechanistic studies.^13^ While binding to the C3 substrate alone has been shown to confer relevant inhibitory activity, simultaneous occupation of compstatin sites on C3 and the AP C3 convertase is expected to essentially contribute to the strong potency as an AP modulator.^13^

The high overall resolution of the cryo-EM structure yielded well-defined density across the entire cyclic peptide backbone and side-chains of Cp60, with the exception of the solvent-facing C-terminal di-Lys motif (Figure 6B). Superimposition of the target-bound structures of Cp60 and Cp40 further illustrates the conserved binding mode between the analogs, featuring highly similar peptide conformation with only minor backbone deviation (Cα RMSD = 0.61 Å; Figure 6C). Importantly, the well-defined density of Cp60 allowed us to directly confirm our in silico predictions that the alkyl extension at position 3 addresses a specific target pocket by engaging in hydrophobic interactions with Leu454 and Met346 of C3b (Figure 6D,E). Thus, Cp60 achieves its ultrahigh target affinity via a finely tuned contact network, even if the overall binding mode is almost identical to the clinical candidate Cp40.^13^ Our experimentally determined C3bB-Cp60 structure provides valuable insight at multiple molecular scales by (1) extending structural detail on compstatin’s target spectrum and mode of action; (2) confirming conserved target interaction sites and contact networks across the compstatin family; and (3) providing a refined understanding of how a minor modification within a short hydrophobic side chain can lead to a substantial gain in affinity.

### Application of Cp60 for Particle- and Cell-Surface Detection of Deposited C3 Fragments

Even if the pronounced impact of the V3I substitution on affinity and potency may suggest a translational value for the development of next-generation compstatin therapeutics, it remains to be examined whether the PK/PD properties of Cp60 are indeed superior to those of the clinical candidate Cp40 (AMY-101). Beyond therapeutic uses, however, Cp60s' exceptional target residence may also enable diagnostic applications of this peptide family for monitoring C3-mediated opsonization of (bio)­surfaces. As a proof-of-concept study to explore the suitability of labeled Cp60 for detecting surface deposition of C3 fragments, we utilized a bead-based model system (Figure 7A). To enable detection, we fluorescently labeled compstatin analogs Cp01, Cp01-V3I, Cp40, and Cp60 by adding *C*-terminal Lys, in which the ε-amino group was functionalized with sulfo-cyanine-5 (sCy5). An opsonized model surface was prepared by capturing C3b, which was site-specifically biotinylated at its TED domain, onto streptavidin-coated beads. After the opsonized beads were incubated with serial dilutions of the labeled peptides, fluorescent signals were quantified by flow cytometry. In agreement with biophysical and functional assays, Cp60 showed superior binding to C3b-coated beads, as indicated by increased mean fluorescence intensity (MFI) at lower peptide concentrations (Figure 7B). As expected, the detection efficacy decreased in direct relation to the analog’s target residence, i.e., Cp60 > Cp40 > Cp01-V3I > Cp01.

**Figure 7:**
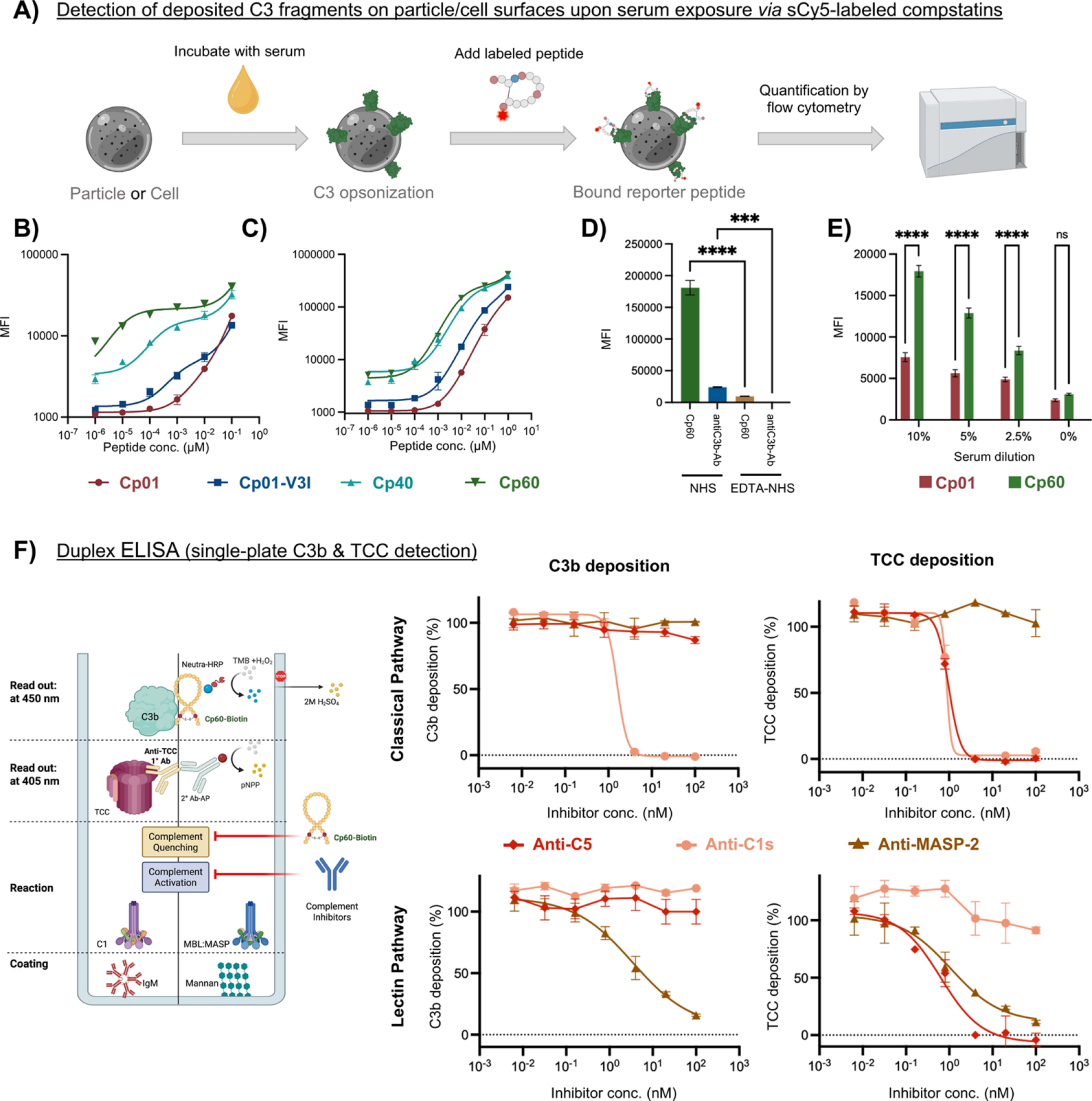
Proof-of-concept study to explore diagnostic applications of compstatin. (A) Assay format used for detecting C3-fragment deposition on particle surfaces upon serum exposure via sCy5-labeled compstatins. (B) Titration of sCy5-labeled compstatins for the detection of C3b-biotin immobilized on streptavidin beads. (C) Titration of sCy5-labeled compstatins for the detection of C3-derived opsonins on beads upon incubation with 50% normal human serum (NHS). (D) Comparative assessment of signal intensities (median fluorescence intensity; MFI) between Cp60-sCy5 and a commercial anti-C3b mAb (clone 3E7; APC-labeled) in the bead assay described above; NHS containing 10 mM EDTA was used as an inactive complement control. (E) Detection of C3-derived opsonins on the surface of porcine iliac-artery endothelial cells (PIEC) by Cp01-sCy5 and Cp60-sCy5 upon incubation with 0–10% NHS. (F) Duplex ELISA format enabling the simultaneous monitoring of opsonization (C3b; Cp60-biotin) and terminal effector generation (TCC; anti-TCC, clone aE11, Hycult) in a single well. Surrogates of clinical mAb with selectivity for the classical (anti-C 1s; sutimlimab), lectin (anti-MASP-2; narsoplimab), and terminal (anti-C5; eculizumab) pathways were used to provide relevant inhibition readouts in CP and LP activation assays.

After establishing and validating the assay using purified C3b, we determined the detection capacity of all peptides for physiologically deposited, serum-derived C3 fragments on the bead surface. To this end, unmodified streptavidin-coated magnetic beads were incubated with 50% normal human serum (NHS) to trigger complement activation and C3 opsonization. Following traditional assay protocols, serum was removed by several wash steps, followed by incubation with sCy5-labeled peptides and flow cytometry (Figure 7A). In analogy to the previous experiment, Cp60 enabled superior detection of deposited C3 fragments at lower concentrations when compared to other compstatin analogs (Figure 7C), thereby supporting the suitability of Cp60 for the detection of C3-derived surface opsonins.

Alongside expected benefits regarding production cost and labeling versatility, the long target residence and comparatively small size may confer additional advantages over antibodies as detection tools. Moreover, compstatin’s capacity to block further complement activation when added to serum in excess of C3 (5 μM in 50% NHS) could enable a direct “quench-and-stain” approach that eliminates the need for initial wash steps to remove serum. To experimentally validate this hypothesis and quantify a potential impact on detection sensitivity, we repeated the bead-based assay and compared the traditional assay format using an established commercial anti-C3b/iC3b detection antibody (clone 3E7, APC-labeled; BioLegend)^35^ with the “quench-and-stain” detection using Cp60-sCy5. When adjusted for emission maxima of the corresponding label, Cp60-sCy5 showed profoundly enhanced detection signals when compared to anti-C3b (Figure 7D). Whether the residual C3b signals measured with Cp60-sCy5 for beads incubated with complement-impaired EDTA serum are related to background activity in the facilitated assay protocol and to sensitive detection of passively absorbed C3 remains to be investigated.

While providing a model for comparing labeled compstatin analogs and optimizing detection methods, magnetic bead assays are rarely used to investigate complement activation on biologically relevant surfaces. To assess the diagnostic potential of Cp60-sCy5 in a more physiological context, we explored its application for monitoring C3-derived opsonization in endothelial cells. For this purpose, we exposed porcine iliac artery endothelial cells (PIEC) to human serum to trigger complement activation.^36^ PIEC were exposed to increasing serum concentrations (0–10%), followed by detection of deposited C3 fragments using 1 μM Cp60-sCy5; as a comparison, we included Cp01-sCy5 that failed to achieve saturation at 1 μM in the bead assays. As expected, the detection of C3-opsonized PIEC cells was superior for Cp60-sCy5 over Cp01-sCy5 at all tested serum concentrations (Figure 7D), thus confirming Cp60s potential as a diagnostic tool to detect C3b/iC3b on cells and other surfaces.

ELISA-type assays that detect markers of opsonization (C3b) or terminal effectors (TCC) upon pathway-specific complement activation in serum are frequently employed in biomedical research and screening of complement inhibitors.^31^ We therefore extended our comparative detection study to CP and LP activation ELISAs, using surrogates of the clinically approved antibodies sutimlimab (anti-C 1s; CP-specific) and narsoplimab (anti-MASP-2; LP-specific) as relevant example for quantitative applications.^37^ Following the protocol for Cp60-sCy5, a biotinylated Cp60 derivative was generated to enable detection via HRP-conjugated NeutrAvidin. As in the bead assay, Cp60-biotin could be directly added to serum in excess (100 nM in 1% NHS) to enable a “quench-and-stain” approach with a reduced number of wash steps. When compared to the traditional protocol using the anti-C3b mAb 3E7, the detection of C3 deposition using Cp60-biotin yielded highly comparable IC_50_ values with strong reproducibility (Figures S35 and S36).

The inherent orthogonality of peptides and antibodies may also be exploited to develop “duplex ELISA” formats. While C3-derived opsonization is a critical biomarker of complement activation and amplification, detection of TCC serves as an indicator of downstream effector generation via the terminal pathway. In an adaptation of the pathway-specific ELISAs, following the direct serum addition of Cp60-biotin, a commercial anti-TCC mAb was added to the same wells. Whereas quantification of C3b deposition was again achieved via the HRP reaction, TCC could be orthogonally detected using a secondary antibody conjugated to alkaline phosphatase. When applied to the CP and LP ELISAs, this dual detection approach captured the distinct intervention points of proximal inhibitors (anti-C 1s, anti-MASP2) in comparison with a terminal pathway blocker (anti-C5; eculizumab surrogate);^38^ as expected, the latter affected only TCC deposition but not C3 opsonization. In combination, these experiments demonstrate that labeled Cp60 may not only serve as an efficient replacement for commonly employed anti-C3 mAb in various assays, with potential benefits regarding cost and ease-of-use, but that the peptide’s functional properties and orthogonality to antibodies can be exploited to develop improved assay formats with streamlined protocols, enhanced sensitivity, and/or duplex readout capacity.

## Discussion

The compstatin family of macrocyclic peptides has emerged as a valuable drug class for treating complement-related diseases.^1^ In the approved drug pegcetacoplan, two compstatin Cp05 peptides are linked by a central 40-kDa PEG to delay renal filtration.^23^ Meanwhile, the clinical candidate AMY-101 is based on the optimized Cp40 scaffold, the enhanced affinity and target residence of which eliminates the need for PEGylation.^7,8,25,26^ While such PK/PD enhancement was enabled by early SAR studies, the fact that some positions within the compstatin sequence have not been fully investigated raised the question of whether bridging SAR gaps could procure further benefit.

Previous SAR studies of compstatin, largely centered on positional scanning,^8,17,18^ revealed that positions 3 and 5–8 were crucial for target interaction. Here, we show that even subtle changes to residues 5–8 using methylene insertion and bioisosteric replacement negatively affects affinity. Our findings confirm that this central region in the peptide is highly optimized with little room for improvement, providing further validation to target contacts unveiled in the Cp40-C3b crystal structure.^13^ In the case of position 3, however, revisiting and extending SAR efforts resulted in a strong enhancement of compstatin’s properties. Although the potential for substituting Val3 by Ile or similar residues had been noted in early intellectual property filings from our laboratories,^27^ experimental assessment of such modifications on the framework of Cp01 or subsequent compstatin generations has not been reported; indeed, all compstatin-based drugs and candidates feature Val at this position. We therefore employed our SAR platform to revisit the originally considered hydrophobic-residue substitutions.^27^ Among these, replacement of Val with Ile in analog Cp01 resulted in vastly improved affinity for C3b and up to 40-fold enhanced potency for inhibiting complement activation in vitro. Our results thus provide the first direct experimental confirmation that a Val-to-Ile substitution enhances target affinity. Given compstatin’s discovery by phage display,^14^ the strong impact of substituting canonical amino acids may appear surprising but could be explained by a lack of sequence coverage in the initial 27-mer phage-displayed peptide library. Indeed, previous canonical amino-acid substitutions in the phage-derived compound (i.e., V4W, H9A, T13I) all resulted in improved potency,^16^ highlighting the importance of continuous SAR studies even for de novo peptide-based inhibitors.

With a target affinity (*K*_D_) of 20 nM and ∼10-fold improvement over the parental analog Cp01,^16^ Cp01-V3I represents the most potent compstatin analog entirely composed of canonical amino acids, based on in vitro performance. In our assays, Cp01-V3I even showed superior affinity over the clinically used Cp05 modality that contains a noncanonical residue. Reliance on proteinogenic amino acids is expected to facilitate biotechnological applications; for instance, all-canonical peptide drugs can be produced by recombinant methods to reduce costs and environmental impact associated with SPPS.^39^ Recombinant expression can also afford alternative PK-enhancement strategies, including peptide–protein fusions with serum albumin (e.g., albiglutide) or IgG-Fc (e.g., dulaglutide).^40^ Additionally, lasso-grafting of de novo canonical cyclic peptides into protein loops has recently emerged as a method for improving PK properties or engineering bispecific inhibitors.^41^ Finally, genes encoding the peptide, or fusion constructs thereof, could be delivered via viral or other vectors as part of gene therapy approaches.^42^ Collectively, Cp01-V3I may open novel opportunities for the design of compstatin-based treatment modalities.

By transferring the productive V3I modification into the optimized Cp40 scaffold, we obtained Cp40-V3I, also termed Cp60. Featuring a *K*_D_ of 80 pM for C3b, Cp60 is the highest-affinity compstatin analog reported so far, surpassing Cp40 in our assays. Given the close similarity between Cp40 and Cp60, differing only in the presence of an extra methyl group in Ile’s side chain when compared to Val, the 10-fold affinity enhancement may appear surprising. While a visual inspection of the contact site on C3b would not point to unoccupied pockets but rather suggest a constrained space, our in silico model revealed a narrow yet defined hydrophobic cleft within the C3b interface that accommodates Ile’s extended side chain. The ideal fit of Ile is supported by the observation that other subtle modifications of side-chain size or stereochemistry result in weaker affinities. While originally anticipated to confirm Cp60's binding mode, the high-resolution cryo-EM structure of the C3bB-Cp60 complex enabled direct experimental validation of the predicted interaction between Ile3 and the hydrophobic pocket in C3b.

Beyond confirming molecular determinants of Cp60s' strong target affinity, the cryo-EM structure also provided valuable mechanistic insights. Given their contact site at the MG4/MG5 domain interface, compstatins bind to native C3 and all fragments and complexes thereof that contain the β-chain.^9,13^ For the mode of action as a PPI inhibitor of C3 activation, engagement with the C3 substrate and the AP C3 convertase (C3bBb) are most relevant.^13^ Since previous target-bound structures of compstatin had been obtained with individual C3 fragments,^9,13^ this work aimed to achieve the first structural validation of compstatin bound to a convertase complex. When cryo-EM is employed, a larger target size is also expected to facilitate single-particle analysis. Given the metastable nature of C3bBb, via irreversible dissociation of Bb,^33^ and the resulting technical challenges for structural studies, we used the AP C3 pro-convertase (C3bB) as a relevant surrogate. Upon surface deposition of C3b, FB binds to the opsonin and transitions between “closed” and “open” conformations, the latter of which facilitates binding of FD to release the Ba fragment and form the active convertase.^43,44^ That compstatins bind to C3b, and the pro-convertase rather than exclusively to the “final” target C3bBb, may well contribute to its potent and preventative activity in impairing AP-mediated amplification. Although structures of nickel-stabilized or ligand-bound C3bB had been reported previously,^44,45^ the cryo-EM structure in this study describes the pro-convertase complex at improved resolution (2.88 Å). The arrangement of C3b-bound FB in our structure aligns closely with the “open” conformation observed in earlier structures of nickel-stabilized and FD-bound C3bB,^44^ yet is distinct from the “closed” conformation stabilized by the inhibitor lufaxin (Figure S37).^45^ Previous studies established that compstatin prevents convertase-binding of C3 without disrupting convertase assembly;^13^ given the distant binding sites, it is improbable that Cp60 would stabilize the “open” conformation of C3b-bound FB. Instead, this state likely reflects the more stable conformer. As expected, our structure also confirms that the presence of FB does not prevent compstatin’s binding to the C3b component of AP C3 convertases, structurally validating C3bB as a target and supporting compstatin’s mode of action.

Notably, the 10-fold affinity enhancement of Cp60 over Cp40 is primarily defined by its slow dissociation rate constant (*k*_d_ = 1 × 10^–4^ s^–1^), resulting in long target residence (τ > 100 min). Beyond improving the PD profile, the remarkable stability of the drug-target complex could potentially also prove beneficial for PK properties. Owing to the high plasma levels of C3 (1–2 g/L),^46^ compstatin analogs with long target residence have been shown to feature extended plasma half-lives, even in the absence of PEGylation, likely due to less free peptide being subjected to renal filtration.^8,47^ In our limited PK study, which involved only 2 animals, a single dose, and no analog comparison, Cp60-KK generally aligned with the expected PK model by featuring a slow terminal elimination phase (*t*_1/2_ ∼ 30–40 h). When compared to previous PK studies in NHP, the half-life of Cp60-KK appears strongly improved over early compstatin analogs such as Cp20 (*t*_1/2_ < 10 h) but, despite its superior target affinity, considerably shorter than the related analog Cp40-KK, with a reported terminal half-life in the range of 100 h.^32^ This may suggest that differences in distribution, tissue sequestration, extravascular space retention, or elimination mechanisms can outweigh the expected target-residence benefit on plasma exposure. Of note, however, such direct quantitative comparison with reported NHP studies of earlier compstatin analogs (Cp20–Cp40)^8^ and Cp40-KK^32^ must be interpreted with utmost caution due to differences in structural modifications (e.g., di-Lys extension), administration route (intravenous vs subcutaneous), study design, and animal numbers. A full characterization of Cp60's clinical PK/PD relationship will therefore require extensive preclinical in vivo studies designed to disentangle these contributions. While the reliance on NHP models precludes such comprehensive profiling within the scope of this study, our preliminary PK analysis of Cp60-KK in 2 cynomolgus monkeys nevertheless supports that this next-generation analog generally follows the affinity-related PK paradigm proposed for the compstatin class,^8^ yet it is not powered to substantiate definitive PK advantages over current drug candidates.

In contrast to PK profiling, which critically relies on relevant animal models (currently, only NHP), ex vivo assays using human donor serum provide a suitable approximation to evaluate PD properties of compstatin analogs.^18,32^ Nevertheless, highly diluted serum conditions are a technical prerequisite for most in vitro complement assays, which impose limitations regarding both the dynamic range and translational value. Along the structural optimization of the compstatin class, an increasing divergence between affinity and potency gains could be observed, in this study and previous reports;^8,16,18,19,32^ our comparative assessment of analogs covering a broad activity range now formally confirms this nonlinear relationship. Increasing the serum concentration in the CP inhibition ELISA emphasized the superior potency of Cp60 over Cp40 (IC_50_ = 97 vs 150 nM), but not to an extent matching the 10-fold affinity improvement. Even if these studies suggest that the lower-than-expected potency gain for Cp60 is more likely related to dynamic range limitations, further studies in more physiological systems and disease models are warranted to obtain a complete PD profile. Alongside target binding and inhibitory efficacy, other parameters need to be considered for assessing the clinical potential of a new compstatin analog, including solubility, stability, and safety. Immunogenicity needs to be considered in this context, even if macrocyclic peptide drugs approved so far do not appear to produce substantial immunogenicity potential.^39^ While the 4 noncanonical amino acids in Cp60 could accentuate immunogenicity, these residues remain unchanged from Cp40 as the basis of the clinical candidate AMY-101, which has completed safety and tolerability assessment as part of first-in-human trials.^7,26^ This study attests AMY-101 to a good safety profile when administered in both single and multiple doses via different routes, with no evidence of drug-related immunogenicity or increase in antidrug antibodies. Although safety profiles need to be obtained for each drug candidate, the positive experience with AMY-101 and the high similarity between the two compounds’ properties and binding modes, as confirmed by our cryo-EM data, could be indicative of a low immunogenic potential.

Owing to its direct descent from and affinity-gain over the clinical candidate Cp40/AMY-101, a discussion of Cp60's clinical potential and limitations is both obvious and warranted. It is important to emphasize, however, that this study aimed to demonstrate the value of continuous SAR and medicinal chemistry campaigns, even in cases of established de novo peptide macrocycles rather than providing a preclinical assessment of a next-generation drug candidate. In fact, clinical development of Cp60 would likely necessitate benefits across a broad range of properties (PK, PD, safety, stability, solubility) over existing candidates such as Cp40/AMY-101,^7,25,26^ or Cp40-KKK/AMY-106.^48^ In this context, the versatility of applications enabled by Val3Ile substitution in the compstatin scaffold should be considered of higher importance.

This broader impact of Val3Ile-mediated affinity enhancement was evident in diagnostic applications. Cp60's remarkable target residence, together with the knowledge that compstatin analogs can be labeled C-terminally without major impact on target binding,^32,47^ motivated an evaluation of labeled Cp60 for the detection of C3-derived opsonization in complement assays. Traditionally, labeled antibodies that specifically recognize activation fragments (C3b, iC3b, C3d) are used for this purpose;^35^ however, antibodies may face limitations regarding production cost, labeling options (e.g., conjugation chemistry, stoichiometry, homogeneity), and, for some applications, tissue penetration. The accessibility of Cp60 for production via chemical synthesis is expected to mitigate labeling constraints, reduce cost, and afford new modalities and applications for the detection of opsonized surfaces.^49^ Indeed, the proof-of-concept studies in this work establish that site-specifically labeled Cp60, carrying either fluorescent dyes or biotin handles, serves as a reliable detection entity to monitor and quantify surface deposition of C3 fragments across ELISA-type, bead-based, and living-cell assay systems. While the potential benefits mentioned above generally apply to high-affinity macrocyclic peptides, Cp60s function as a potent inhibitor of complement amplification confers another intriguing advantage. Due to the self-propagating mechanism of C3b opsonization, wash steps are typically required to remove serum and stop amplification before the addition of the detection antibody. We could now show that the tight binding of Cp60 to C3b deposits enables a “quench-and-stain” approach that eliminates the need for initial wash steps, thereby facilitating assay procedures. In the case of the bead-based assay, which reflects microparticle and biomaterial applications, the use of Cp60-sCy5 resulted in profound sensitivity gain when compared to detection via anti-C3b mAb. Among the potential reasons for this unexpectedly strong impact is the peptide’s better accessibility to binding sites when compared to antibodies that may be sterically restricted, especially on densely opsonized surfaces. The ability to use biotinylated Cp60 instead of antibodies also proved advantageous in ELISA-type complement assays, with 1 mg of Cp60-biotin providing enough material for more than 40,000 assay points in 96-well plates. Moreover, the new “duplex ELISA” format enables a simultaneous monitoring of two complement biomarkers in a single assay plate, which was previously constrained to separate assay setups.^31^ Of note, this approach is even compatible with commercial TCC complement activation kits that use alkaline-phosphatase detection, such as WIESLAB assays.^50^ We envisage that duplex ELISA could be particularly useful for activity profiling of complement inhibitors with undefined targets, differentiating proximal and terminal intervention in a single assay.

These proof-of-concept studies impressively illustrate the potential of labeled compstatin analogs, and in particular Cp60, for diagnostic applications. Most of the formats shown so far were directly converted from standard protocols without further optimization; standardized assays resulting from such efforts need to be fully validated regarding reproducibility, sensitivity, and dynamic range, among other parameters. Of note, there are some intrinsic limitations associated with a diagnostic use of labeled Cp60. Compstatin’s narrow species specificity for human and NHP C3 currently restricts its use as a detection entity in most animal models.^13,51^ Moreover, compstatin-mediated readouts will be unable to distinguish between C3b and its degradation fragment iC3b when monitoring opsonization, or between C3 and C3c as soluble markers, as these forms of C3 all contain the compstatin site.^9,13^ Similarly, compstatins will not detect late-stage opsonin C3d, which may comprise a dominant form in certain live-cell assays or tissue staining. In most cases, such limitations are shared with marker-specific antibodies, though. At the same time, the strategy may be extended to other applications; peptides in general have emerged as promising scaffolds for diagnostic purposes, due to exquisite control of labeling choice, stoichiometry, and homogeneity.^49^ Beyond fluorescent dyes, options include metal-chelators to enable radiolabeling, or highly sensitive time-resolved Förster resonance energy transfer reporters.^52^

## Conclusions

Enabled by continuous optimization of the macrocyclic peptide scaffold, the compstatin family of C3 inhibitors has found broad application in biomedical research and drug therapy. The SAR studies presented in this work and the first high-resolution structure of a compstatin-bound convertase complex, provide important validation regarding drug–target interaction determinants of the compstatin family. Guided by this insight and in silico predictions, we identified the promising V3I modification that enhanced the affinity of tested compstatin analogs by up to 30-fold. While a potent complement inhibitor by itself, analog Cp01-V3I’s composition of only canonical amino acids also facilitates recombinant production and provides opportunities for exploiting biotechnological strategies in PK/PD enhancement or drug targeting. When transferred to the clinical candidate Cp40, the V3I substitution yielded analog Cp60 featuring low picomolar affinity; this marks not only the most potent compstatin analog but also one of the highest-affinity peptidic PPI inhibitors reported to date. While it remains open whether Cp60s superior in vitro properties can inform next-generation clinical candidates, its exceptional target residence positions it as an intriguing entity for diagnostic applications to monitor C3-opsonization of cell and biomaterial surfaces. Labeled Cp60 demonstrated equivalent or superior detection performance to antibody reagents, while its complement-inhibition properties enabled extended assay formats. Collectively, this work highlights the value of continuous, vigorous SAR profiling of clinical peptides, solidifies our understanding of compstatin’s binding mode and functional properties, and adds new biotechnological and diagnostic opportunities to the ever-growing application range of the compstatin class.

## Experimental Section

### Peptide Synthesis

Peptides were prepared by automated SPPS, cyclized by H_2_O_2_, and purified by preparative HPLC based on previously established methods and detailed in the supplementary methods.^13^ Isolated peptides were analyzed by HPLC (Agilent 1100) using a reversed-phase C18 column (Waters Atlantis T3, 2.1 × 150 mm, 100 Å, 3 μm) held at 30 °C. A 5–95% gradient of acetonitrile (+0.1% TFA) in water (+0.1% TFA) was applied over 30 min; analytes were detected using a UV/vis detector at 214 nm (and 650 nm for sCy5-labeled peptides). Peptide identities were confirmed by positive electrospray ionization mass spectrometry (ESI+ MS). Unless otherwise stated, the purity of all peptides is >95% as determined by the relative integration of the target peak in the chromatogram recorded at 214 nm. Analytical HPLC and ESI+ MS data are provided in the Supporting Information (Figures S38–S73).

Stock solutions for bioassays were prepared by dissolving lyophilized peptides in water and measuring the UV absorbance at 280 nm using a UV spectrophotometer (DS-11, DeNovix). Stock concentrations were determined using the calculated molar extinction coefficient at 280 nm for each peptide analog (PepCalc.com).

### Kinetic Binding Studies

Compstatin analogs were tested for their binding affinity and kinetic profiles by surface plasmon resonance (SPR) using a Biacore T200 instrument (Cytiva, Piscataway, NJ). Experiments were conducted at 25 °C in HBST buffer (10 mM HEPES, 150 mM NaCl, and 0.05% Tween-20, pH 7.4; XanTec bioanalytics). Human C3b (Complement Technology, Tyler, TX, USA) was immobilized via amine-reactive coupling. After activation of carboxymethyldextran hydrogel chips (CMD500 or CMD200L; XanTec bioanalytics, Düsseldorf, Germany), C3b in sodium acetate buffer pH 4.5 was injected into two flow cells until densities of 4000–6000 resonance units (RU) were reached. The reference flow cell was activated with the same method, and the remaining active sites on all flow cells were blocked with ethanolamine. For immobilization, the flow rate was set at 10 μL/min, and for all interaction experiments, at 30 μL/min.

An initial peptide screening was conducted at concentrations of 2 and 1 μM. Peptides showing relevant binding at those concentrations were tested in serial 2-fold dilutions of concentration in a multicycle kinetics experiment, with a regeneration phase (1 M NaCl for 30 s) between injections. Individual peptide samples were injected for 120 s, followed by a 180 s dissociation phase. Analog Cp01 was included in each experimental series as an internal control. Signals from the reference cell and buffer blank injections were subtracted within each dilution series to correct for buffer effects and injection artifacts. Data were fitted to a Langmuir 1:1 binding model to calculate association (*k*_a_) and dissociation (*k*_d_) rate constants, and the equilibration constant *K*_D_ is calculated based on the equation: *K*_D_ = *k*_d_/*k*_a_.

In the case of Cp40 derivatives and Cp60, a single-cycle approach was used for kinetic analysis by injecting 5 increasing concentrations of each compound (0.5–40 nM) consecutively without a regeneration period in between injections. Individual injections within a cycle were 120 s long with a 5 min dissociation phase between injections. After the highest concentration of each derivative, a dissociation period of 100–240 min was added to complete dissociation and establish baseline conditions since no suitable regeneration method could be identified. Analog Cp40 was included in each experimental series as a reference and internal control. Signals from the reference cell and buffer blank injections were subtracted to correct for buffer effects and injection artifacts. Kinetic data were processed in BiaEvaluation (Cytiva) using single-cycle evaluation by global fitting each data set to a Langmuir 1:1 binding model.

The reported kinetic parameters (Tables 1 and 2) indicate the mean values and standard deviations of three independent experiments. SPR sensorgrams are provided in the Supporting Information.

### Complement Inhibition Assays (ELISA)

The inhibitory activity of selected peptides in preventing pathway-specific complement activation was evaluated using ELISA as previously described.^31^ All assays used normal human serum (NHS), pooled from five unrelated, anonymized healthy donors, obtained with informed consent according to the local ethics committee, and following the guidelines of the Declaration of Helsinki (Blutspendezentrum Basel, Switzerland).

To assess classical pathway activity, flat-bottom MaxiSorp 96-well plates (Thermo Fisher) were coated overnight at room temperature with 33 μg/mL IgM (Sigma) in a 0.1 M sodium carbonate buffer. Plates were washed 5 times with phosphate-buffered saline (Dubleccòs, Bioconcept) supplemented with 0.05% Tween-20 (PBS-T). Serial dilutions of peptides were prepared in 1% (v/v) NHS using HBBT++ assay buffer (10 mM HEPES, 150 mM NaCl, pH 7.4, 0.5% BSA, 0.15 mM CaCl_2,_ 0.5 mM MgCl_2_, 0.1% Tween-20), with samples diluted in 1% (v/v) NHS. Negative controls included each inhibitor in assay buffer alone, assay buffer only, and 1% (v/v) NHS with 10 mM EDTA. HBBT++ with 1% (v/v) pooled NHS served as the 100%-control. After incubating for 1 h at 37 °C and shaking at 700 rpm, plates were washed 5 times with PBS-T. Next, 100 μL of biotinylated monoclonal mouse antihuman TCC antibody (1:1000, Hycult, clone: aE11) to detect terminal complement complexes was added to each well, followed by 1 h incubation at room temperature while shaking. After another 5 washes with PBS-T, 100 μL of HRP-conjugated NeutrAvidin (1:10,000, Invitrogen) was added to each well and incubated for 20 min at room temperature while shaking. Plates were washed 5 more times with PBS-T before adding 100 μL of 1-Step Turbo TMB-ELISA solution (Thermo Scientific) to each well. The reaction was allowed to develop until a light blue color appeared, and then stopped with 100 μL of 2 M sulfuric acid. Colorimetric detection was performed at 450 nm by using a plate reader (Synergy HT, Biotek). All assays were conducted in triplicate and normalized against 100% (no inhibitor, 1% (v/v) serum in buffer) and 0% (1% (v/v) serum in EDTA buffer) controls. In the cases of Cp60 and Cp40, 5% (v/v) NHS was used instead of 1% (v/v) NHS in some assays. Inhibition curves were fitted using nonlinear regression to a normalized variable slope model in Prism (v9-10, GraphPad).

The activity of selected peptides in preventing the activation of the alternative pathway was evaluated by analogy to the classical pathway ELISA described above, with some adjustments. Flat-bottom PolySorb 96-well plates (Thermo Fisher) were coated overnight at room temperature with 40 μg/mL lipopolysaccharide from *Salmonella enteritidis* (Sigma-Aldrich) in 0.1 M sodium carbonate buffer and washed 5 times with PBS-T. Serial dilutions of peptides were prepared in 10% (v/v) pooled NHS using HBBT + assay buffer (10 mM HEPES, 150 mM NaCl, pH 7.4, 0.5% BSA, 5 mM EGTA_,_ 5 mM MgCl_2_, and 0.1% Tween-20), with samples diluted in 10% (v/v) NHS. Negative controls included each inhibitor in assay buffer alone, assay buffer only, and 10% (v/v) serum with 10 mM EDTA. HBBT+ with 10% (v/v) pooled NHS served as the 100%-control. Subsequent steps were performed as described above.

### In Silico Structural Analysis

Crystal structures of Cp01 (PDBID: 2QKI)^9^ and Cp40 (PDBID: 7BAG)^13^ were prepared using Maestro (Schrödinger Drug Discovery Suite, v. 2024-3). In order to limit the crystal structures to the relevant regions for this study, the protein was truncated to include only domains MG1, MG4, and MG5, as well as part of the LNK domain. Specifically, we included residues 1–105 (MG1), 590–643 (LNK), and 329–536 (MG4/MG5). All crystallization additives, such as glycerol and bromine ions, were removed. Protein Preparation Wizard was used to assign bond orders, add hydrogens, create disulfide bonds, cap the termini of the protein, and generate net states with Epik at pH 7.4 ± 0.1. After the initial preparation, the same tool was used to optimize the hydrogen bond network with Propka and minimize the structures with OPLS 2005 to an RMSD of 0.3 Å.^53,54^ The resulting structures were the starting points for the introduction of mutations on the ligand side.

For each of the substituted analogs tested in vitro, the corresponding position on the ligand side was replaced with the new amino acid. In cases where different rotamers were possible, the built-in rotamer library was used to assess the likelihood of each rotamer. If a clear decision could not be made, then the different rotamers were analyzed individually. In the case of a substitution to an unnatural amino acid, the favorable rotamers devoid of steric clashes were explored manually. After incorporating the change, each analog was reminimized with OPLS 2005 to an RMSD of 0.3 Å to relieve steric clashes on both the ligand side and the protein side.^54^ Finally, the structures were then evaluated in Maestro by expert visual analysis.

All residue substitutions were designed by using structure-guided medicinal chemistry considerations, focusing on hydrophobic side chains with related steric profiles but distinct conformational preferences. Particularly at position 3, Val, Ile, allo-isoleucine, and 2,2-diethylalanine were selected to systematically probe the effects of β-branching, side-chain topology, and conformational restriction on the ability of the residue to adopt rotamers compatible with the bound conformation while maintaining steric complementarity with the hydrophobic pocket. This small set of analogs was intended to modulate the side-chain shape and accessible rotamer space without substantially altering the overall physicochemical properties of the peptide scaffold.

### Pharmacokinetic Analysis of Cp60-KK in Nonhuman Primates

The in vivo studies were conducted at the Simian Conservation Breeding and Research Center (SICONBREC) in Makati, Philippines. Two male cynomolgus monkeys (*Macaca fascicularis*), aged 6–7 years and weighing approximately 4 kg, were used. Animals were acclimatized for 2 weeks in sterilized ILAR type 3 stainless steel cages under controlled environmental conditions (26 ± 4 °C, 60 ± 25% humidity, natural light cycle, and proper ventilation). They were fed 100 g/day of a standard monkey grower pellet (Jetstar Milling Corp.) and had ad libitum access to water. Bananas were provided daily as a dietary supplement. On dosing day, food was offered post administration.

The peptide was administered via subcutaneous (sc) injection at a dose of 2 mg/kg. Cp60-KK (8 mg each) were dissolved in sterile saline, and injections were delivered using 3/10 mL insulin syringes with 29G × 1/2″ needles, at a dose of 2 mg/kg. For sample collection, blood was drawn from the femoral vein at predose (0 h) and postdose time points: 5 min, 30 min, 1, 2, 4, 6, 12, 24, 48, 72, 96, and 120 h. Samples were collected in EDTA tubes, centrifuged at ∼800 × *g* for 10 min, and plasma was stored at −80 °C. Details on the determination of Cp60-KK plasma concentration and PK modeling can be found in the Supporting Information.

All in vivo studies followed ethical guidelines and were reviewed and approved by the Institutional Animal Care and Use Committee (Philippines; approval number: 2019-09). SICONBREC is accredited by the International Association for Assessment and Accreditation of Laboratory Animal Care (AAALAC) for providing and maintaining a high-quality program of animal care and use (animal research permit: AR-2019-357).

### Structure Determination by Cryogenic Electron Microscopy (Cryo-EM)

#### Complex Preparation

Plasma-purified C3b and factor B (both Complement Tech), and compstatin analog Cp60-KK were mixed at a 1:1:2 molar ratio for a final complex concentration of 3 μM in buffer containing 20 mM Tris–HCl, pH 7.5, 140 mM NaCl, and 10 mM NiCl_2_ for 1 h at 4 °C. For the resulting C3bB-Cp60-KK Inhibitor complex, 3 μL of sample was applied to Quantifoil R1.2/1.3 300 Mesh Copper grids (Quantifoil, Germany) that were negatively glow-discharged at 15 mA for 60 s using a PELCO easiGlow Glow Discharge Cleaning System at the Electron Microscopy Resource Lab (EMRL) of the University of Pennsylvania. The grids were blotted with PELCO qualitative cellulose filter paper (Grade 595 55/20 mm) for 3 s, vitrified with a Vitrobot Mark IV (Thermo, USA) at 4 °C and 95–100% chamber humidity using a blot force of 2, and immediately plunged into liquid ethane cooled by liquid nitrogen. The sample quality and particle distribution on the grids were assessed using EPU (Thermo) operating a 200 kV Glacios 2 cryo-TEM (Thermo) equipped with a Falcon 4i direct electron detector and a Selectris energy filter.

#### Single-Particle Data Acquisition and Image Processing

Cryo-EM data were collected at the Beckman Center for Cryo-Electron Microscopy (University of Pennsylvania) using a 200 kV Glacios 2 transmission electron microscope (Thermo), equipped with a Falcon 4i direct electron detector and a Selectris energy filter with a 20 eV slit width. Movies were acquired in counting mode using EPU at a nominal magnification of 130,000×, corresponding to a calibrated pixel size of 0.9343 Å (image dimensions: 4096 × 4096 pixels). Data collection was performed over a defocus range of −0.3 to −2.1 μm in 0.2 μm increments. The dose rate was 7.23 e^–^/Å^2^/s, resulting in a total accumulated dose of 43.36 e^–^/Å^2^ recorded as 1845 electron events. A total of 6993 micrographs were recorded and saved in electron-event representation (EER) format.

A workflow for image processing of the C3bB-Cp60-KK complex is found in Figure S33. The EER exposures were imported and processed in CryoSPARC^55^ with EER fractionation set to 40 and EER upsampling set to 1. Movies were motion corrected, gain-normalized, drift-corrected, summed, dose-weighted, and converted into image stacks using the Patch Motion Correction module (saved in 16-bit floating point). Contrast transfer function (CTF) values of the motion-corrected movies were estimated using the Patch CTF module. Micrographs with thick ice, ethane contamination, poor CTF fit resolution (>8 Å), and high astigmatism (≥1000 Å) were discarded. After micrograph curation, 6791 micrographs were denoised for particle picking using CryoSPARC blob picker with dimensions of 80–180 Å, a 0.2 minimum separation distance, and a local maxima of 2000 picks and subsequent particle extraction using a box size of 360 px, Fourier cropped to 96 px. Initially picked particles were aligned and sorted using 3 rounds of 2D classification (*n* = 80) to remove poorly aligned particles and contaminants. Particles from high-quality 2D classes were used to generate initial reference-free maps using ab initio reconstruction (*n* = 5), which generated 2 maps representing the pro-AP convertase complex (C3bB) and 3 “junk” particle classes. The particles from the C3bB classes were subjected to a final hard 2D classification to generate 2D templates (*n* = 40) for repicking of particles using CryoSPARC template picker (160 Å particle diameter), resulting in 1,475,831 particles. Template-picked particles were subjected to 3 iterative rounds of heterogeneous refinement (*n* = 5 classes), using the C3bB maps from ab initio reconstruction as reference and 3 “junk” classes as decoys to improve particle sorting. The best particles (833,935) were re-extracted at full resolution (360-pixel box size) and subjected to an additional round of heterogeneous refinement, followed by removal of duplicate particles (20 Å minimum separation distance), resulting in a final subset of 732,106 particles. At this stage, 2 distinct C3bB conformations were observed: one containing intact density for the FB serine protease (SP) domain and another lacking discernible SP-domain density. Particles corresponding to the latter class were excluded from further analysis. The remaining 501,139 particles representing the intact C3bB-Cp60-KK complex were subjected to nonuniform refinement for downstream analysis. Inspection of the resulting 3D reconstruction revealed incomplete and anisotropic density for the flexible thioester-containing domain (TED). To improve density quality in this region, a soft mask encompassing the TED was generated and used for focused 3D classification (simple mode, 5 Å filter resolution, *n* = 6 classes). This classification yielded 3 classes with partially resolved TED density, 1 class with largely unresolved TED density, and 2 classes with well-resolved TED density, adopting slightly distinct conformations, all of which were engaged by Cp60-KK. Particles belonging to classes with weak or absent TED density were excluded from further analysis. Particles from the 2 classes exhibiting fully resolved TED density were processed independently and subjected to iterative rounds of nonuniform refinement incorporating both global and local CTF refinement, followed by reference-based motion correction. These procedures resulted in reconstructions at 2.88 and 3.00 Å resolution for TED conformations 1 and 2, respectively. To maximize map quality for the Cp60-KK peptide, particles from both TED-resolved classes were combined and subjected to a final round of nonuniform refinement, yielding a reconstruction at an overall resolution of 2.82 Å.

#### Postprocessing of cryo-EM Maps

Postprocessing of all of the cryo-EM volumes was performed by EMReady using the default parameters.^56^ The reported resolutions of the cryo-EM maps are based on the Fourier shell correlation (FSC) 0.143 criterion.^57^ Local resolution calculations were generated using CryoSPARC Local Resolution Estimation, and color displayed in ChimeraX.^34^ The angular distribution of particle orientations, conical FSC (cFSC) and cFSC Area Ratio (cFAR) summary plots, and weighted distribution of viewing directions were calculated by the CryoSPARC Orientation Diagnostics module (Figure S34).

#### Model Building and Structure Refinement

An initial model for the AP pro-convertase, C3bB, was derived from the Protein Data Bank (PDB ID 2XWJ)^44^ and manually docked into the respective cryo-EM maps using USCF Chimera.^34^ Initial coordinates and model fitting for Cp60-KK were derived from the Cp40 coordinates in the C3b:Cp40 crystal structure (PDB ID 7BAG).^13^ Restraints for Cp60-KK, namely unnatural amino acids (^1‑Me^Trp (EXL), ^*N*‑Me^Ile (IML), and ^*N*‑Me^Gly (SAR)) were generated from phenix.elbow using PHENIX 1.21.2 ^58^ and manually refined within Coot v.0.9.8.96.^59^ Model building was further assisted by iterative rounds of real-space structure refinement using PHENIX 1.21.2 ^58^ with Ramachandran and secondary structure restraints to obtain the final structures.

Cryo-EM data collection, refinement, and validation statistics are listed in Table S2.

### Bead-Based C3b Detection Assay (Flow Cytometry)

To prepare site-specifically labeled C3b, native C3 was activated via limited proteolysis, and the exposed thioester moiety was conjugated with biotin. For this purpose, human C3 (1 mg/mL, CompTech) in PBS was incubated with 1.1 μg/mL trypsin in HCl (Promega) and 100 μg/mL hydrazide-PEG_4_-biotin (EZ-Link, Thermo). The mixture was incubated at 37 °C and 700 rpm for 13 min; subsequently, 10 μg/mL soybean trypsin inhibitor (SBTI; GIBCO) was added, and the mixture was incubated on ice for 30 min.

Magnetic streptavidin-coated beads (Dynabeads M-270, Thermo Fisher) were washed twice with PBS containing 1% (w/v) BSA (Sigma-Aldrich) and 0.005% (v/v) Tween-20 (PBS-BT). In a twin-tec PCR plate (Eppendorf), 8 μL of a 10-fold-diluted bead solution was incubated with 1 μM biotinylated C3b protein solution in PBS-BT for 30 min at RT. PBS-BT alone was used as a control. For the deposition of plasma-derived C3 opsonization fragments, beads were instead incubated with 50% NHS (v/v) in PBS-BT for 30 min at 37 °C. The beads were washed using magnetic plate separation 3 times with PBS-BT and incubated with sCy5-labeled Cp01, Cp01-V3I, Cp40, or Cp60 as serial dilutions (1 or 0.1–0 μM) in PBS-BT for 30 min at 37 °C while shaking (750 rpm). Beads were washed 5 times, resuspended in 100 μL of PBS-BT, transferred into 96-well plates (Agilent), and measured by flow cytometry using a CytoFLEX B4-R3-V0 instrument (Beckman Coulter). Signals were gated on the beads based on forward-scatter-height (FSC-H) and sideward scatter-height (SSC-H). Every experiment was performed at least two times independently with the same setup, and each sample was measured in duplicate.

For the “quench-and-stain” procedure, Cp60-sCy5 was added directly to the serum (5 μM final concentration) for 15 min without prior washing. 50% NHS (v/v) in PBS-BT supplemented with 10 mM EDTA served as an inactivated control. Detection with an anti-C3b/iC3b mAb solution (clone 3E7, mouse IgG1, APC-labeled; BioLegend, final concentration 200 ng/mL) upon 30 min incubation was used for comparison. All other steps remained as described above; however, using 3 wash cycles instead of 5.

### Cell-Based Opsonization Assay (Porcine Endothelial Cell Line)

Porcine iliac-artery endothelial cells (PIEC; Cytion) were maintained in standard T-75 flasks on M199 medium (GIBCO, Bleiswijk, The Netherlands) containing 10% (v/v) fetal bovine serum (GIBCO) and 1% penicillin-streptomycin solution (BioConcept) in a humidified 5% CO_2_ atmosphere at 37 °C. The same cell culture medium was used for incubation and some wash steps throughout experiments, if not mentioned otherwise. For in vitro experiments, PIEC culturing was performed in a manner to not exceed 12 passages starting from the first cryo-vial thawing.

PIEC were seeded onto a 96-well cell culture plate at a density of 30,000 cells per well in 200 μL of M199 growth media. After overnight incubation, cells were washed twice with cell culture medium and treated with serial dilutions of 10 to 2.5% (v/v) NHS in Hank’s balanced salt solution (HBSS; GIBCO) containing 0.15 mM CaCl_2_ and 0.5 mM MgCl_2_ (HBSS^+2^ buffer) or in HBSS^+2^ buffer alone for 1 h at 37 °C. After incubation, cells were washed 3 times with HBSS^+2^ buffer and incubated with sCy5-labeled Cp01 and Cp60 peptides (1 μM in HBSS^+2^) for 30 min at 37 °C. After removing the peptide solution, cells were washed 3 times with HBSS (without Ca^2+^ and Mg^2+^), and 50 μL of a 10 mM EDTA solution was added to each well for 40 min to detach cells. After this final step, the fluorescent signal from sCy5 of either peptide was measured by flow cytometry, as described for the bead assay. Signals were gated on the cells based on forward scatter-height (FSC-H) and sideward scatter-height (SSC-H). Every experiment was performed at least 2 times independently with the same setup, and each sample was measured in duplicates.

### Duplex ELISA System (C3 and TCC Detection)

Simultaneous monitoring of complement activation markers at the level of C3b-deposition and terminal complement complex (TCC) formation was assessed using biotinylated Cp60 and a mouse antihuman TCC mAb (clone aE11, Hycult), respectively. To evaluate classical pathway activity, flat-bottom MaxiSorp 96-well plates (Thermo) were coated overnight at room temperature with 33 μg/mL IgM (Sigma-Aldrich) diluted in 0.1 M sodium carbonate buffer. For lectin pathway activity, plates were coated overnight with 55 μg/mL mannan (from *Saccharomyces cerevisiae*; Sigma-Aldrich). Plates were washed 5 times with phosphate-buffered saline (PBS; Dulbecco’s, Bioconcept) supplemented with 0.05% Tween-20 (PBS-T). For proof-of-concept experiments, sutimlimab (anti-C1s), narsoplimab (anti-MASP-2), and eculizumab (anti-C5; all MedChemExpress) were prepared as 1:5 serial dilutions with a maximum concentration of 500 nM. Inhibitors were diluted in HBBT++ assay buffer (10 mM HEPES, 150 mM NaCl, pH 7.4, 0.5% BSA, 0.15 mM CaCl_2_, 0.5 mM MgCl_2_, 0.1% Tween-20) containing 1% (v/v) NHS. Negative controls included inhibitor in assay buffer alone, assay buffer only, and 1% (v/v) NHS supplemented with 10 mM EDTA. HBBT++ buffer containing 1% (v/v) pooled NHS served as the 100% activity control. Plates were incubated for 1 h at 37 °C with shaking at 700 rpm. Complement activation was quenched by adding 100 nM Cp60-biotin in PBS-T for 10 min, followed by 5 washes with PBS-T. For TCC detection, 100 μL of anti-TCC mAb (1:1000) was added to each well and incubated for 1 h at room temperature with shaking. After washing 5 times with PBS-T, HRP-conjugated NeutrAvidin (1:10,000, Invitrogen) was mixed with alkaline phosphatase-conjugated goat anti-mouse IgG (1:10,000, Novex) and added to the plates for 20 min at room temperature with shaking. Alkaline phosphatase yellow pNPP substrate (Sigma-Aldrich) was added, and the colorimetric reaction was monitored at 405 nm by using a plate reader (Synergy HT, BioTek) at 37 °C for 15 min. Following TCC quantification, plates were washed 5 times with PBS-T, and 1-Step Turbo TMB-ELISA solution (Thermo Scientific) was added for C3b quantification. The reaction was allowed to develop until a light blue color appeared and was stopped by adding 100 μL of 2 M sulfuric acid; the absorbance was measured at 450 nm.

All assays were normalized to 100% activity (1% (v/v) serum in buffer without an inhibitor) and 0% activity (1% (v/v) serum in an EDTA buffer). Inhibitory dose–response curves were fitted using nonlinear regression with a normalized variable-slope model in GraphPad Prism (versions 9-10).

## Supplementary Material











## Data Availability

Cryo-EM maps and atomic coordinates were deposited in the Electron Microscopy Data Bank (EMDB) under accession codes EMD-75834 and EMD-75835, and in the Protein Data Bank (PDB) under accession codes 11MG and 11MH, for C3bB-Cp60 TED conformation 1 and C3bB-Cp60 TED conformation 2, respectively.

## Notes

Supplementary figures and methods (PDF)
Structural coordinates for predicted model of Cp01-Val3Ile bound to C3b (PDB)
Structural coordinates for predicted model of Cp01-Val3aIle bound to C3b (PDB)
Structural coordinates for predicted model of Cp01-Val3Dea bound to C3b (PDB)
Table of molecular strings and associated biophysical data for tested compstatin analogs (CSV)

## ∥

Société des Produits Nestlé, Nestlé Research, Lausanne, Switzerland

## Abbreviations

AP: alternative pathway
C3G: C3 glomerulopathy
CP: classical pathway
cryo-EM: cryogenic electron microscopy
ELISA: enzyme-linked immunosorbent assay
FB: factor B
FD: factor D
HBA: hydrogen bond acceptor
HBD: hydrogen bond donor
HPLC: high-performance liquid chromatography
Ig: immunoglobulin
LP: lectin pathway
MAC: membrane attack complex
MFI: median fluorescence intensity
MG: macroglobulin
NMR: nuclear magnetic resonance
PD: pharmacodynamics
PDB: protein data bank
PEG: polyethylene glycol
PIEC: porcine iliac artery endothelial cell
PK: pharmacokinetics
PNH: paroxysmal nocturnal hemoglobinuria
PPI: protein–protein interaction
SAR: structure–activity relationship
SARS-CoV-2: severe acute respiratory syndrome coronavirus 2
sCy5: sulfo-cyanine 5
SPPS: solid-phase peptide synthesis
SPR: surface plasmon resonance
TCC: terminal complement complex
