# Supplementary material for: New Analogs of the Compstatin Family of Clinical Complement Inhibitors with Low Picomolar Target Affinity
Source: J Med Chem. 2026 May 1;69(9):11592–609. doi: 10.1021/acs.jmedchem.6c00832 (PMC13181761; doi:10.1021/acs.jmedchem.6c00832)
Supplement: Supplementary file 1 [file jm6c00832_si_001.pdf]

Supporting Information

New analogs of the compstatin family of clinical complement inhibitors with low picomolar target affinity

Stephanie A. Vogt[‡a], Alexander J. Lander[‡a], Karl Herbine[b], Ekaterina Umnyakova[a], Jannes Felsch[a], Roman Aschwanden[a], Sarah E. Hughes[b], Oliver Schwardt[a], Markus A. Lill[a], Martin Smieško[a], John D. Lambris*[b], Christina Lamers*[a,c] & Daniel Ricklin*[a]

[a]Department of Pharmaceutical Sciences, University of Basel, 4056 Basel, Switzerland; [b]Department of Pathology & Laboratory Medicine, Perelman School of Medicine, University of Pennsylvania, 401 Stellar Chance, 422 Curie Blvd, Philadelphia, 19104, PA, USA; [c]Institute for Drug Discovery, Faculty of Medicine, Leipzig University, Brüderstrasse 34, 04103 Leipzig, Germany

Correspondence

E-mail: d.ricklin@unibas.ch
E-mail: christina.lamers@medizin.uni-leipzig.de
E-mail: lambris@pennmedicine.upenn.edu

Table of Contents

I. Screening of Cp01 analogs against C3b by SPR S4

Figure S1. SPR sensorgrams from two-concentration screening of Cp01 analogs against C3b by SPR. S4

II. Multi-cycle kinetic binding assays of selected Cp01 analogs against C3b by SPR S9

Figure S2. Cp01 ...... S9

Figure S3. Cp01 Val3Leu S9

Figure S4. Cp01 Val3Tbg S9

Figure S5. Cp01 Val3Ile S9

Figure S6. Cp01 Val3Abu S10

Figure S7. Cp01 Val3Nva S10

Figure S8. Cp01 Asp6Ser S10

Figure S9. Cp01 Asp6Asn S10

Figure S10. Cp01 Asp6Glu S11

Figure S11. Cp01 Asp6Asu S11

Figure S12. Cp01 Trp7Bta S11

Figure S13. Cp01 G8dAla S11

Figure S14. Cp01 Arg11Lys S12

Figure S15. Cp01 Arg11Ser S12

III. Structural analysis of Cp01 Trp4Bta substitution and the role of Trp H-bond donation S13

Figure S16. Measured H-bond donor angle ($\alpha_{C\text{-}N\cdots O}$ = 122.6°) and distance ($d_{N\cdots O}$ = 2.6 Å) of the Cp01 Trp4 interaction with C3b Met457 C=O in the crystal structure (PDB: 2QKI). S13

Figure S17. Comparative search in the Cambridge structural database (CSD) for H-bond donor interactions of 1H-pyrrole (Trp4 representative) and chalcogen bond interactions of thiophene (Bta4 representative) with carbonyl groups. Scatter plots indicate the distributions of interaction distances ($d_{N\cdots O}$ and $d_{S\cdots O}$; 2···1) and angles ($\alpha_{C\text{-}N\cdots O}$, $\alpha_{C\text{-}S\cdots O}$; 3-2···1). S13

IV. Combinatorial modifications of compstatin analogs S14

Figure S18. Representative multi-cycle kinetic SPR experiment of Cp05 against immobilized C3b, with serial two-fold dilutions of concentration (15.6-1000 nM). S14

Figure S19. Representative single-cycle kinetic SPR experiment of Cp05 V3I against immobilized C3b, with serial two-fold dilutions of concentration (0.5-40 nM). S14

Figure S20. Two representative single cycle kinetic SPR experiments of Cp40 against immobilized C3b, with serial two-fold dilutions of concentration (0.5-40 nM). S15

Figure S21. Two representative single cycle kinetic SPR experiment of Cp60 against immobilized C3b, with serial two-fold dilutions of concentration (0.5-40 nM). S15

Figure S22. Inhibition curves of Cp40 and Cp60 in classical pathway complement activation ELISA with 1% normal human serum. S15

Figure S23. Correlation plot of the apparent potency (IC_{50}) determined by CP ELISA in 1% serum, and the C3b affinity (SPR K_D) by activity profiling of ten compstatin analogs of varying affinities (K_D 0.08-552 nM). S16

V. In-vivo NHP studies: Quantification of Cp60-KK in SGI.011 NHP Plasma S17

Figure S24. Quantification of peptide concentrations in the plasma of cynomolgus monkeys. S18

Table S1. Experimental PK parameters of Cp60-KK S18

VI. Aliphatic amino acid analogs at Cp01 and Cp40 position 3 ...... S19

Figure S25. Representative single cycle kinetic SPR experiment of Cp01 3Dea against immobilized C3b, with serial two-fold dilutions of concentration (0.03-2 µM). S19

Figure S26. Representative single cycle kinetic SPR experiment of Cp01 3aIle against immobilized C3b, with serial two-fold dilutions of concentration (0.03-2 µM). S19

Figure S27. Representative single cycle kinetic SPR experiment of Cp40 3Dea against immobilized C3b, with serial two-fold dilutions of concentration (0.5-40 nM). S19

Figure S28. Representative single cycle kinetic SPR experiment of Cp40 3aIle against immobilized C3b, with serial two-fold dilutions of concentration (0.5-40 nM). S20

Figure S29. Inhibition curves of Cp40 3Dea and Cp40 3aIle in classical pathway complement activation ELISA with 1% normal human serum. S20

Figure S30. Inhibition curves of Cp40 3Dea and Cp40 3aIle in alternative pathway complement activation ELISA with 10% normal human serum. S20

Figure S31. Structural visualization of Cp01-Val3 interaction with C3b Leu454, derived from the X-ray crystal structure (PDB: 2QKI). S21

Figure S32. Structural visualization of Cp01-Val3Ile interaction with C3b Leu454, derived from the predicted model in silico. S21

VII. Cryo-EM analysis of Cp60-KK-C3bB complex S22

Figure S33. Cryo-EM data processing workflow for C3bB-Cp60-KK S22

Figure S34. Cryo-EM quality assessment of C3bB-Cp60-KK TED Conformation 1 S23

Table S2. Cryo-EM data collection, refinement, and validation statistics of C3bB-Cp60-KK S24

VIII. Application of biotinylated Cp60 for C3 detection in ELISA S25

Figure S35. Biotinylated Cp60 as detection tool in lectin pathway ELISA. S25

Figure S36. Biotinylated Cp60 as detection tool in classical pathway ELISA. S25

IX. Comparison of experimental C3bB structures S26

Figure S37. Overlay of the C3bB:Cp60-KK structure resolved in this work with previously reported C3bB structures. S26

X. Supplementary materials and methods S27

XI. Peptide HPLC chromatograms and ESI+ mass spectra S29

I. Screening of Cp01 analogs against C3b by SPR

Figure S1. SPR sensorgrams from two-concentration screening of Cp01 analogs against C3b by SPR.

Analogs that failed to reach an Rmax of >5 RU at 2 μM in both experiments were characterized as non-binders (N.B.) (2 μM, green; 1 μM, red). Repeated experiments are located adjacently.

Cp01

Cp01 Val3Ala

Cp01 Val3Leu

Cp01 Val3Aib

Cp01 Val3Tbg

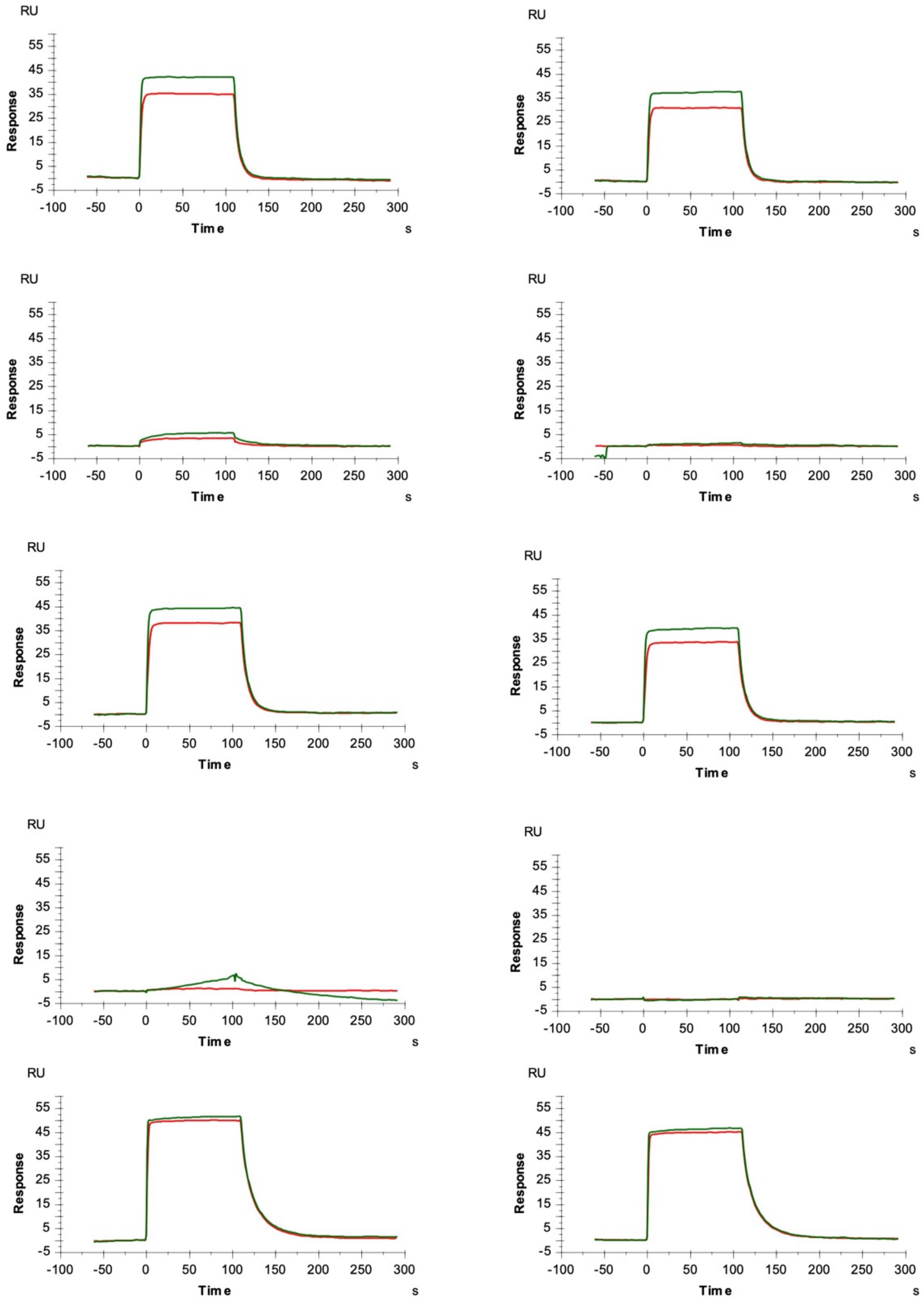

Cp01 Val3Trp

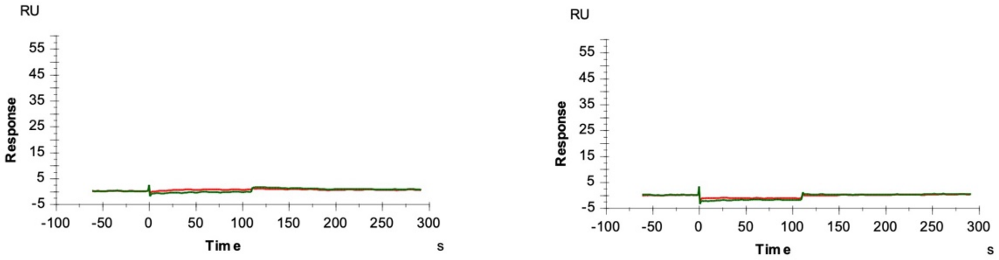

Cp01 Val3Phe

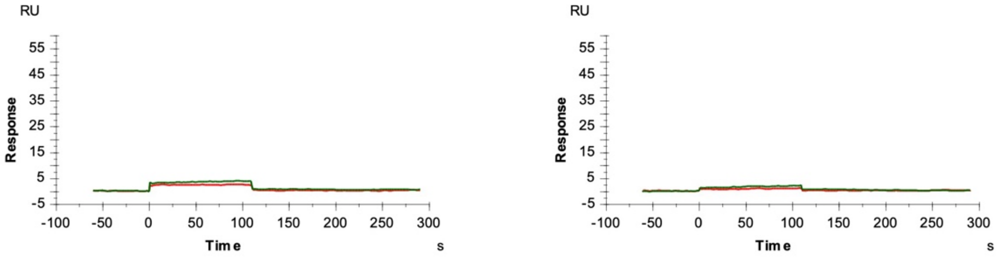

Cp01 Val3Tyr

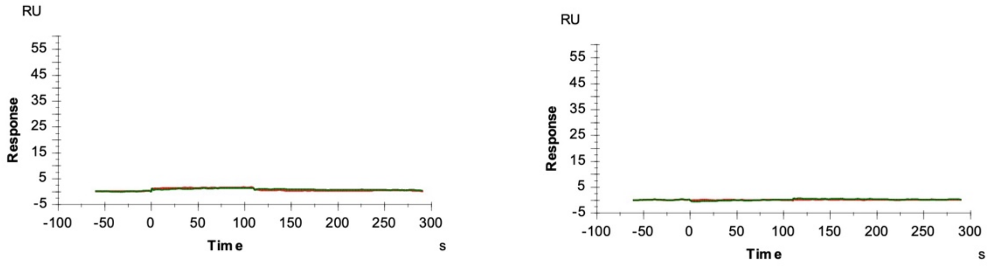

Cp01 Val3Ile

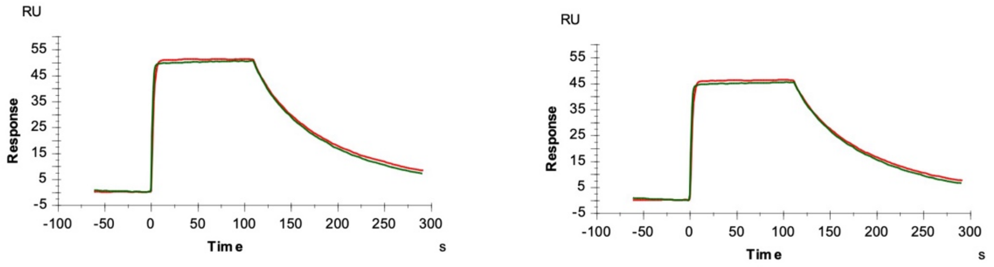

Cp01 Val3Abu

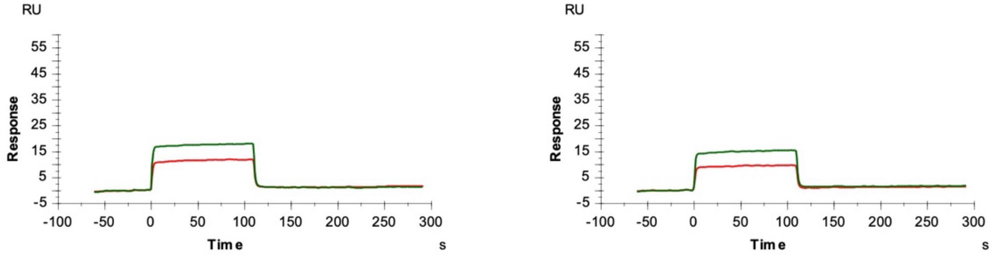

Cp01 Val3Nva

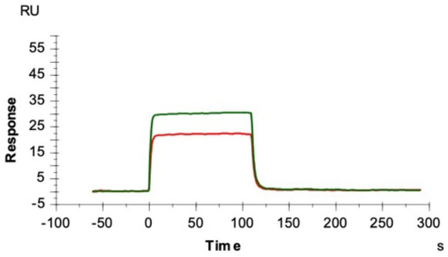

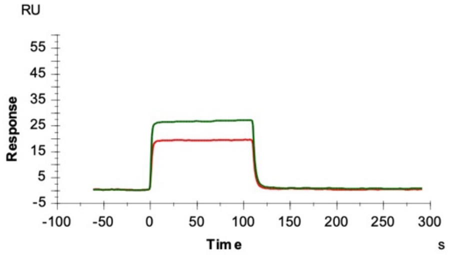

Cp01 Gln5Lys

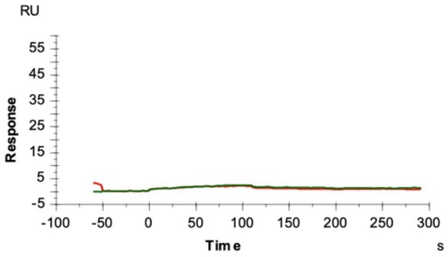

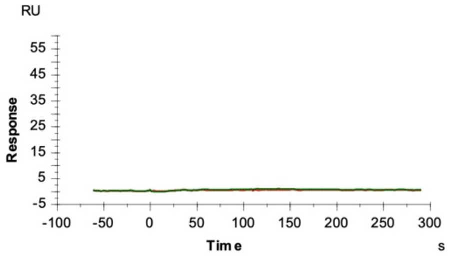

Cp01 Gln5Orn

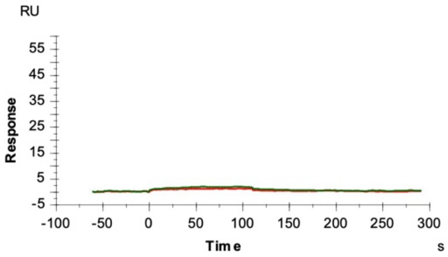

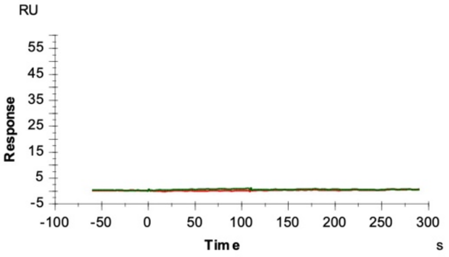

Cp01 Asp6Ser

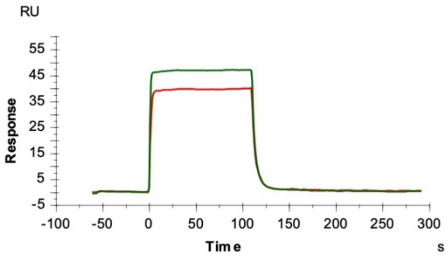

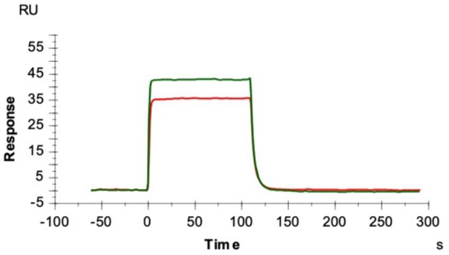

Cp01 Asp6Asn

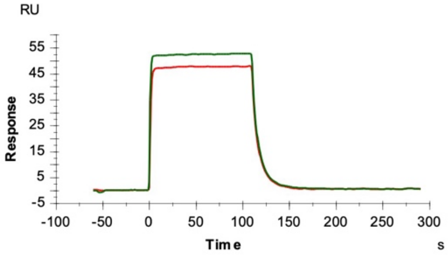

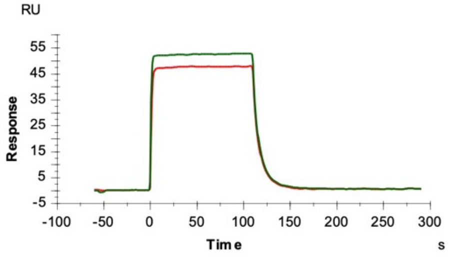

Cp01 Asp6Glu

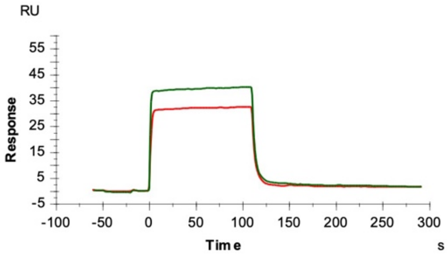

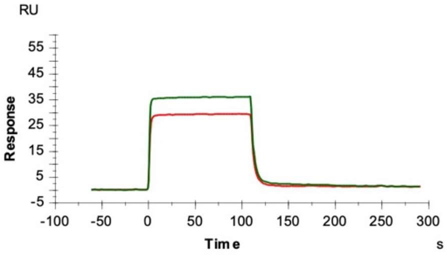

Cp01 Asp6Asu

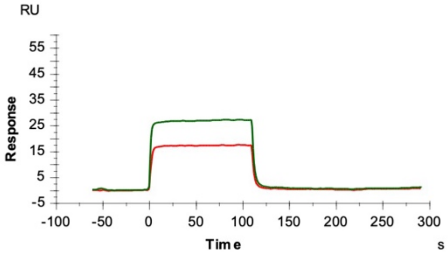

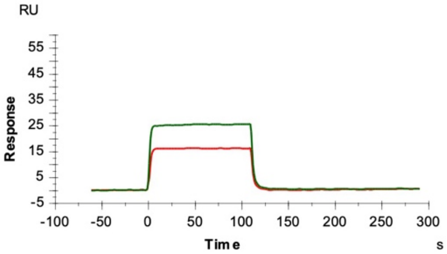

Cp01 Trp7Bta

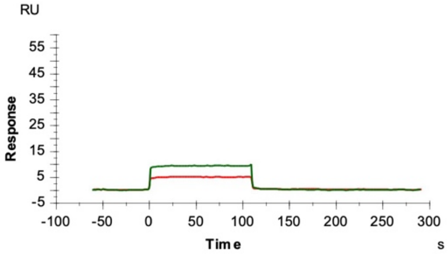

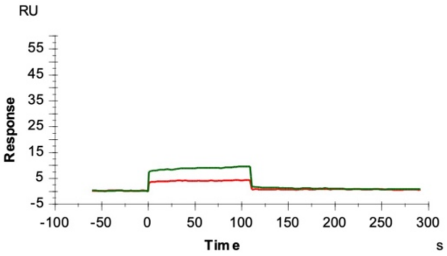

Cp01 Gly8Ala

Cp01 Gly8DAla

Cp01 Arg11Lys

Cp01 Arg11Ser

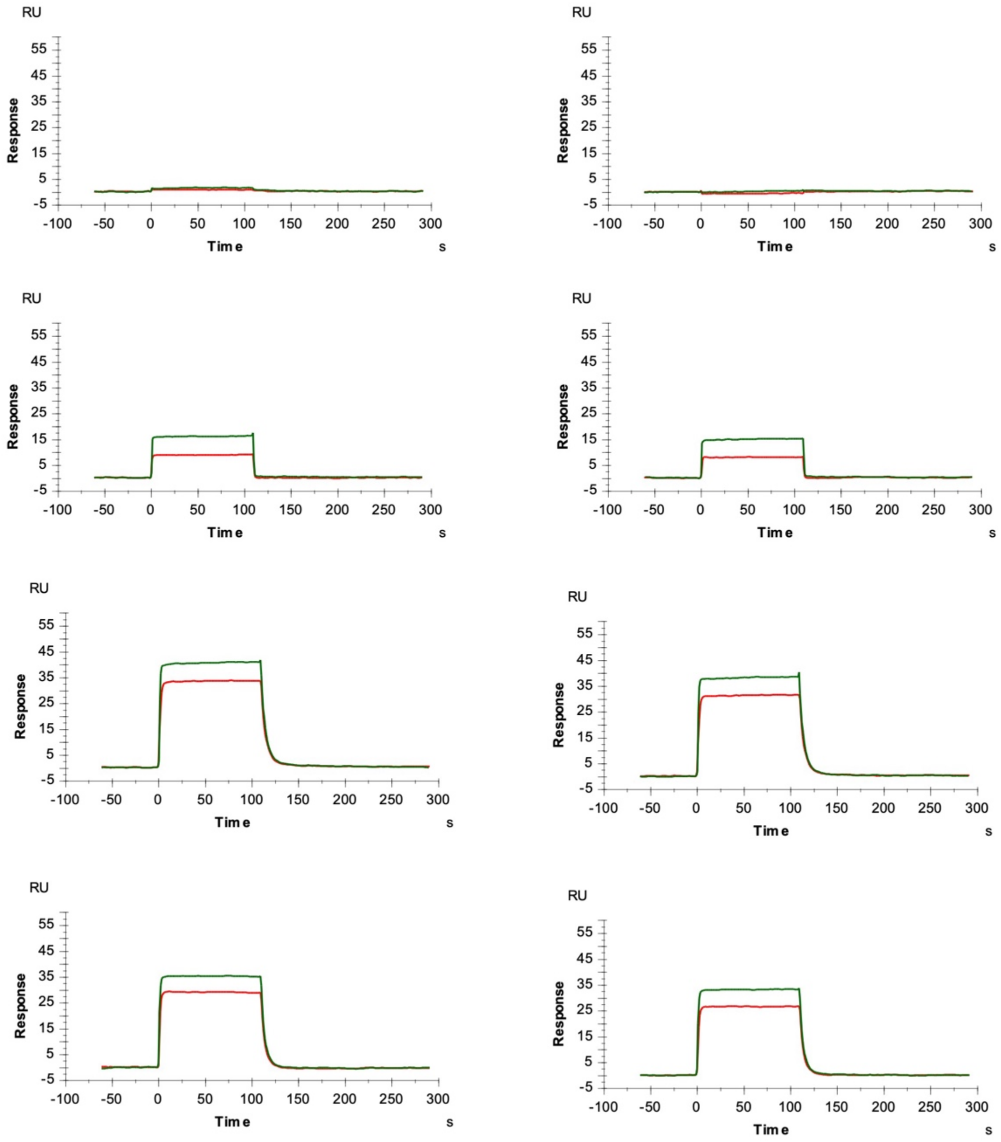

II. Multi-cycle kinetic binding assays of selected Cp01 analogs against C3b by SPR

Figure S2. Cp01

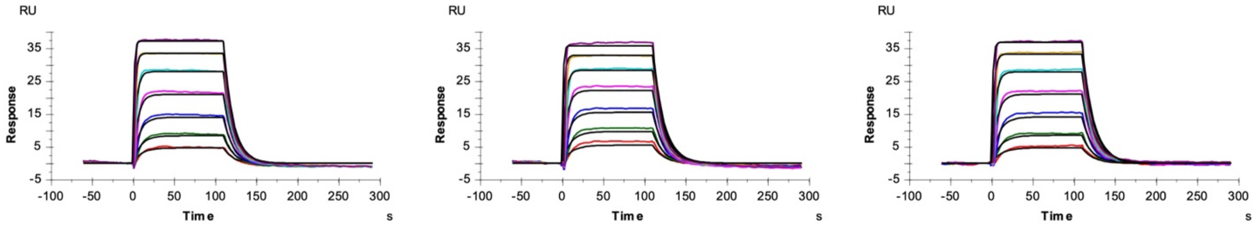

Three independent multi-cycle kinetic SPR experiments of **Cp01** against immobilized C3b, with serial two-fold dilutions of concentration (0.03-2 μM).

Figure S3. Cp01 Val3Leu

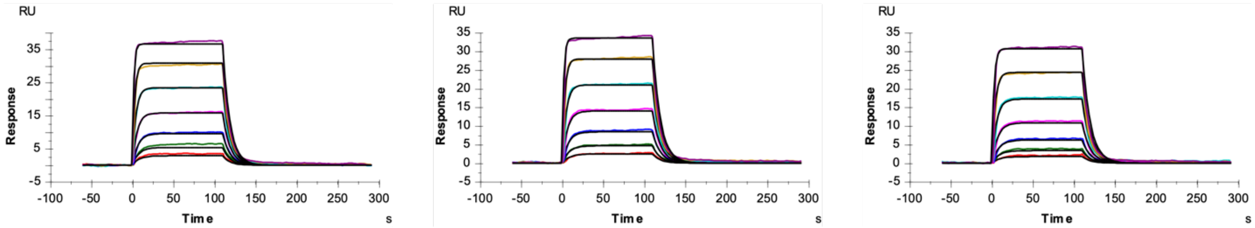

Three independent multi-cycle kinetic SPR experiments of **Cp01 Val3Leu** against immobilized C3b, with serial two-fold dilutions of concentration (0.03-2 μM).

Figure S4. Cp01 Val3Tbg

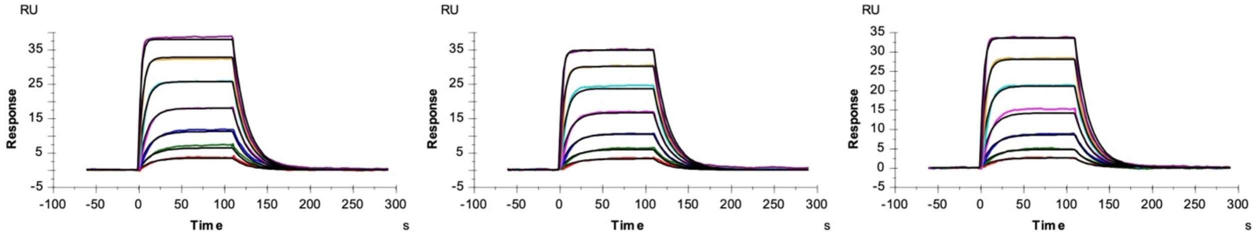

Three independent multi-cycle kinetic SPR experiments of **Cp01 Val3Tbg** against immobilized C3b, with serial two-fold dilutions of concentration (0.03-2 μM).

Figure S5. Cp01 Val3Ile

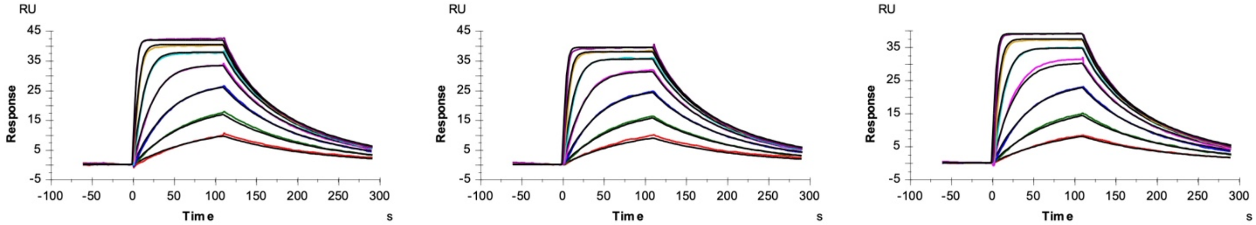

Three independent multi-cycle kinetic SPR experiments of **Cp01 Val3Ile** against immobilized C3b, with serial two-fold dilutions of concentration (0.008-0.5 μM).

Figure S6. Cp01 Val3Abu

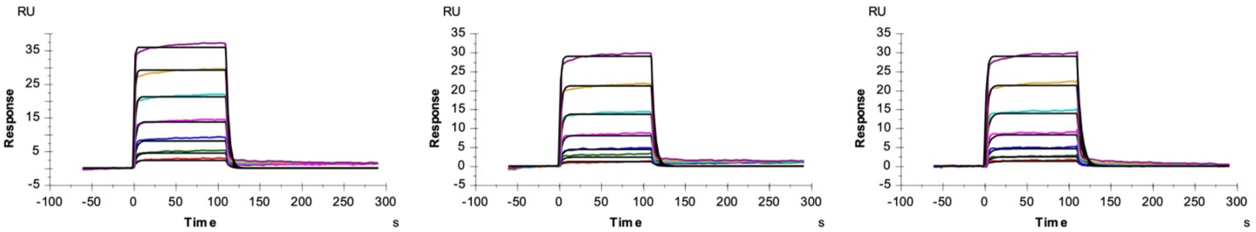

Three independent multi-cycle kinetic SPR experiments of **Cp01 Val3Abu** against immobilized C3b, with serial two-fold dilutions of concentration (0.25-16 μM).

Figure S7. Cp01 Val3Nva

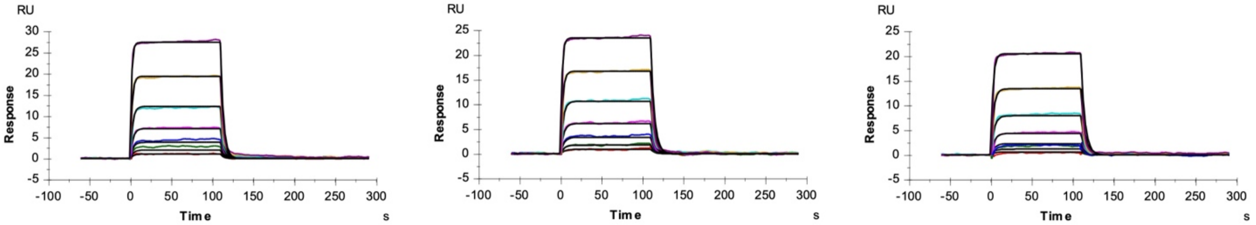

Three independent multi-cycle kinetic SPR experiments of **Cp01 Val3Nva** against immobilized C3b, with serial two-fold dilutions of concentration (0.03-2 μM).

Figure S8. Cp01 Asp6Ser

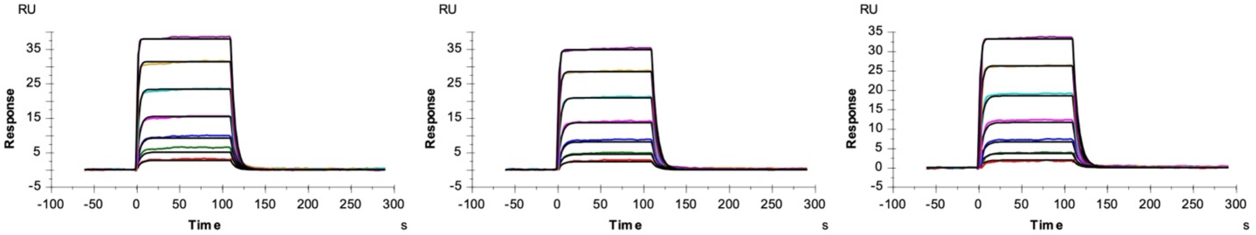

Three independent multi-cycle kinetic SPR experiments of **Cp01 Asp6Ser** against immobilized C3b, with serial two-fold dilutions of concentration (0.03-2 μM).

Figure S9. Cp01 Asp6Asn

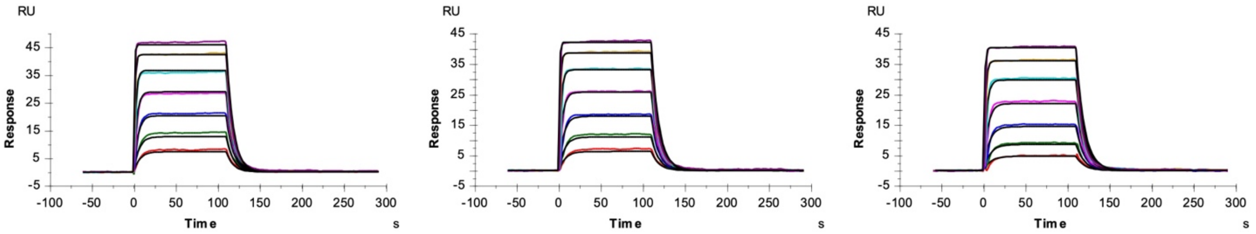

Three independent multi-cycle kinetic SPR experiments of **Cp01 Asp6Asn** against immobilized C3b, with serial two-fold dilutions of concentration (0.03-2 μM).

Figure S10. Cp01 Asp6Glu

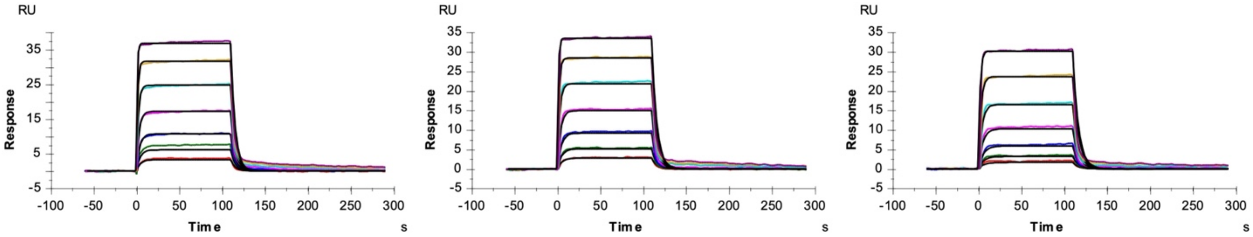

Three independent multi-cycle kinetic SPR experiments of **Cp01 Asp6Glu** against immobilized C3b, with serial two-fold dilutions of concentration (0.03-2 μM).

Figure S11. Cp01 Asp6Asu

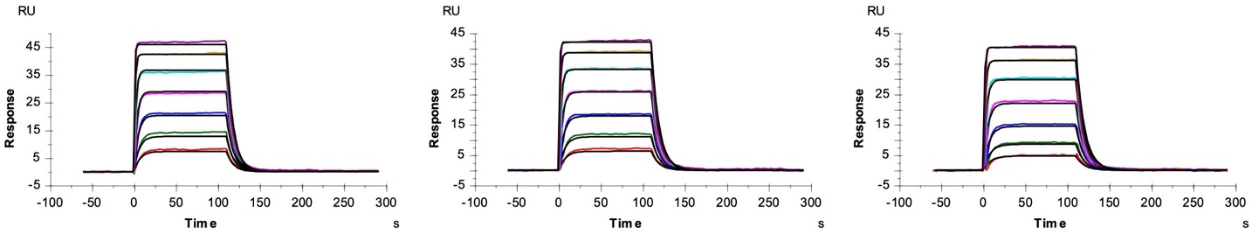

Three independent multi-cycle kinetic SPR experiments of **Cp01 Asp6Asu** against immobilized C3b, with serial two-fold dilutions of concentration (0.06-4 μM).

Figure S12. Cp01 Trp7Bta

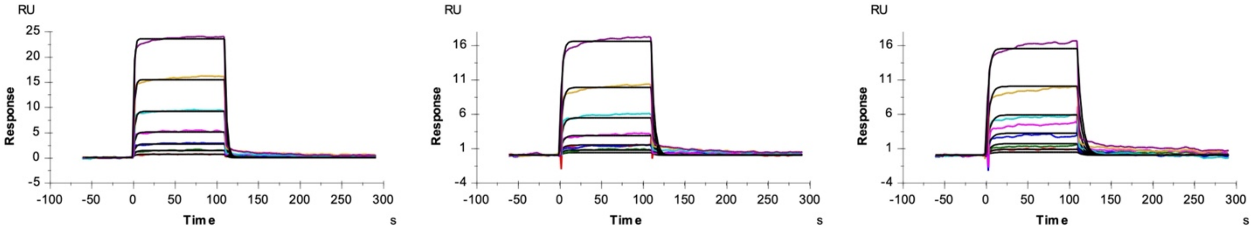

Three independent multi-cycle kinetic SPR experiments of **Cp01 Trp7Bta** against immobilized C3b, with serial two-fold dilutions of concentration (0.25-16 μM).

Figure S13. Cp01 G8dAla

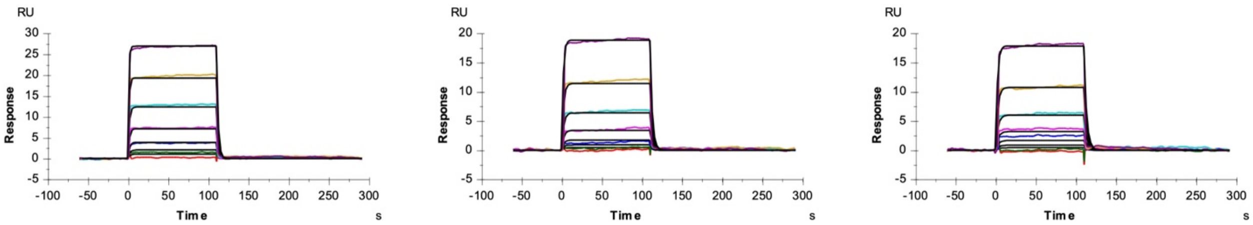

Three independent multi-cycle kinetic SPR experiments of **Cp01 G8dAla** against immobilized C3b, with serial two-fold dilutions of concentration (0.25-16 μM).

Figure S14. Cp01 Arg11Lys

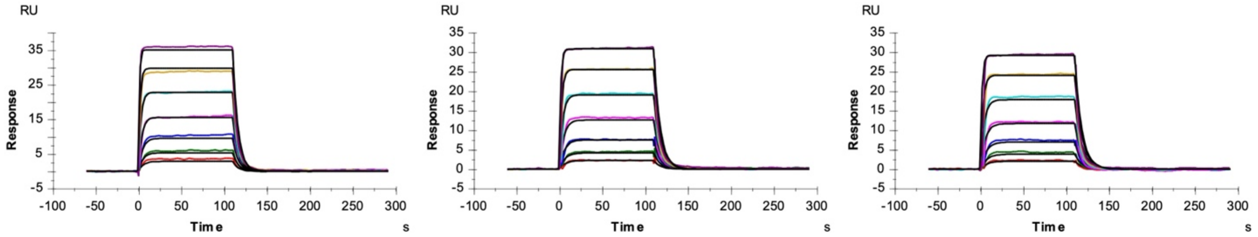

Three independent multi-cycle kinetic SPR experiments of **Cp01 Arg11Lys** against immobilized C3b, with serial two-fold dilutions of concentration (0.03-2 μM).

Figure S15. Cp01 Arg11Ser

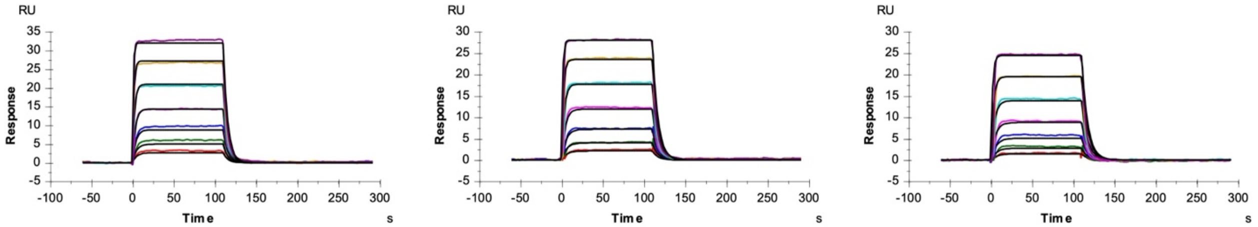

Three independent multi-cycle kinetic SPR experiments of **Cp01 Arg11Ser** against immobilized C3b, with serial two-fold dilutions of concentration (0.03-2 μM).

III. Structural analysis of Cp01 Trp4Bta substitution and the role of Trp H-bond donation

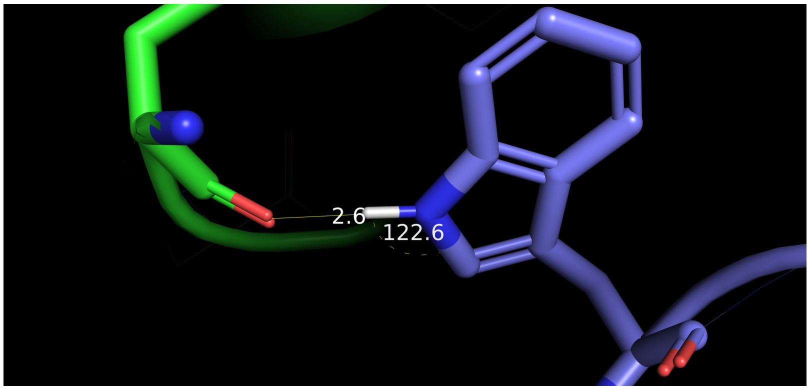

Figure S16. Measured H-bond donor angle ($\alpha_{C\text{-}N\cdots O}$ = 122.6°) and distance ($d_{N\cdots O}$ = 2.6 Å) of the Cp01 Trp4 interaction with C3b Met457 C=O in the crystal structure (PDB: 2QKI).

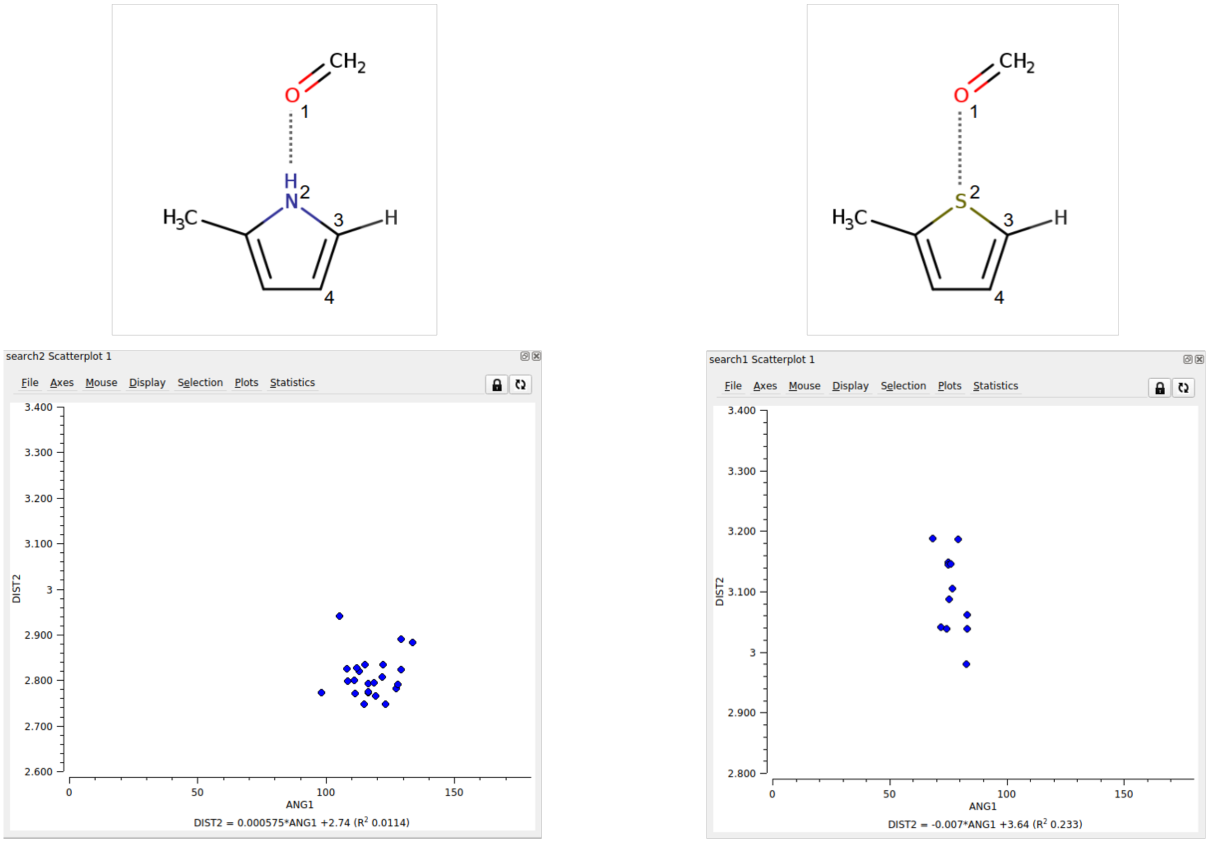

Figure S17. Comparative search in the Cambridge structural database (CSD) for H-bond donor interactions of 1H-pyrrole (Trp4 representative) and chalcogen bond interactions of thiophene (Bta4 representative) with carbonyl groups. Scatter plots indicate the distributions of interaction distances ($d_{N\cdots O}$ and $d_{S\cdots O}$; 2•••1) and angles ($\alpha_{C\text{-}N\cdots O}$, $\alpha_{C\text{-}S\cdots O}$; 3-2•••1).

IV. Combinatorial modifications of compstatin analogs

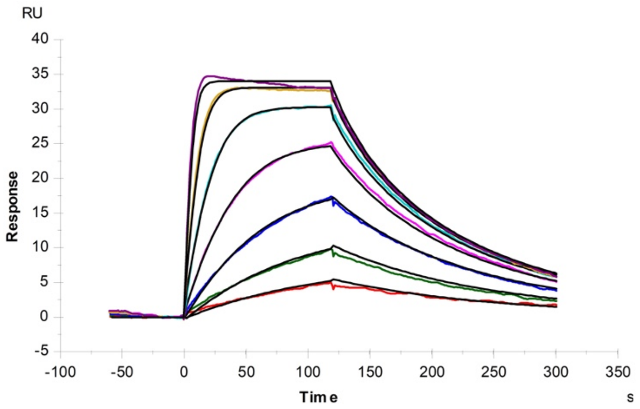

Figure S18. Representative multi-cycle kinetic SPR experiment of Cp05 against immobilized C3b, with serial two-fold dilutions of concentration (15.6-1000 nM).

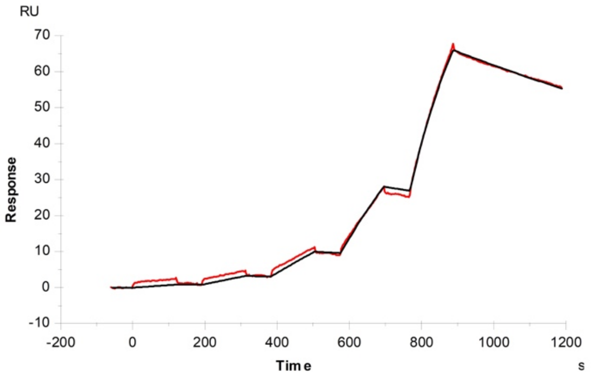

Figure S19. Representative single-cycle kinetic SPR experiment of Cp05 V3I against immobilized C3b, with serial two-fold dilutions of concentration (0.5-40 nM).

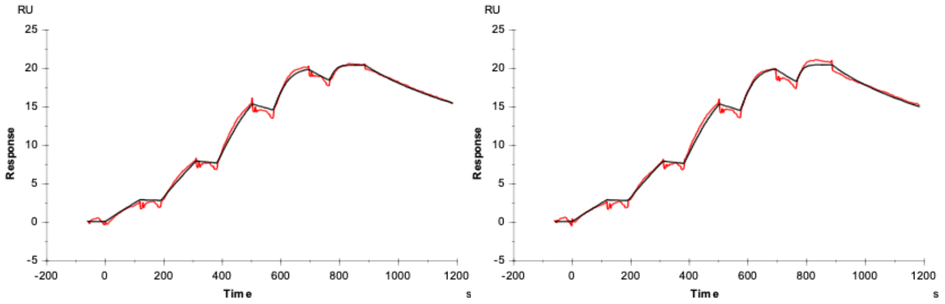

Figure S20. Two representative single cycle kinetic SPR experiments of Cp40 against immobilized C3b, with serial two-fold dilutions of concentration (0.5-40 nM).

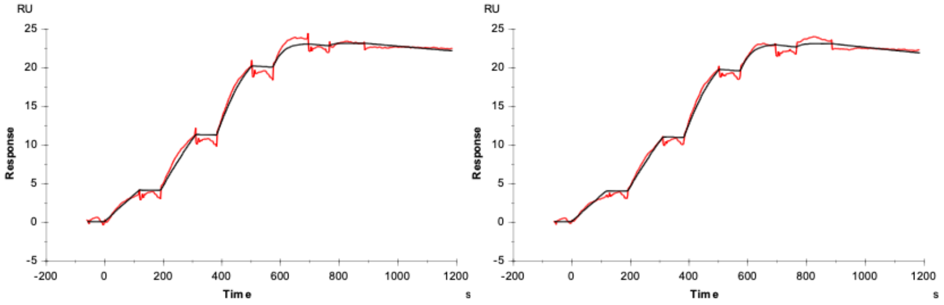

Figure S21. Two representative single cycle kinetic SPR experiment of Cp60 against immobilized C3b, with serial two-fold dilutions of concentration (0.5-40 nM).

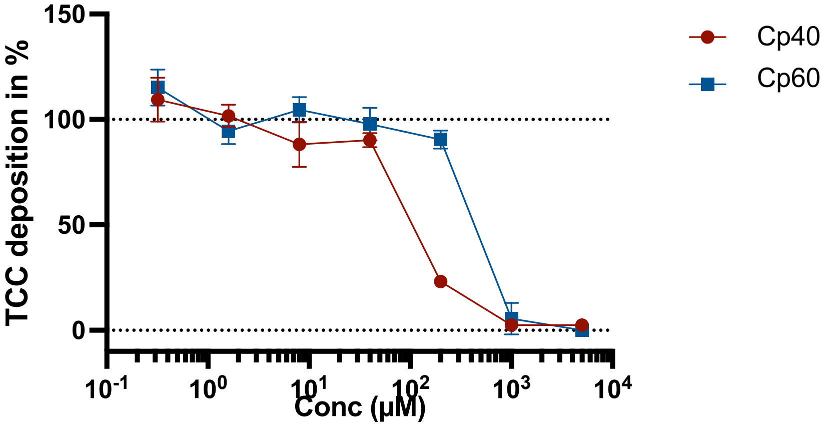

Figure S22. Inhibition curves of Cp40 and Cp60 in classical pathway complement activation ELISA with 1% normal human serum.

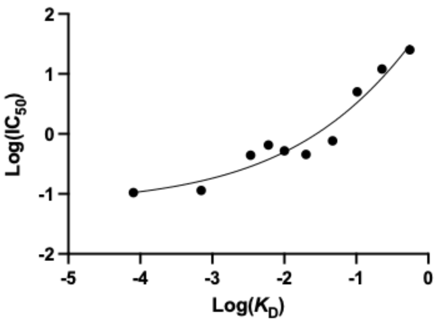

Compstatin analog	C3b K_D (nM)	CP IC_{50} (μM)
Cp01 3L	552	25.3
Cp01	228	12.1
Cp01 3Tbg	103	5.03
Cp05	47	0.766
Cp01 3I	20	0.456
Cp40 12S	10	0.524
Cp40 7S	6	0.651
Cp20	3.4	0.440
Cp40	0.7	0.114
Cp60	0.08	0.106

Figure S23. Correlation plot of the apparent potency (IC_{50}) determined by CP ELISA in 1% serum, and the C3b affinity (SPR K_D) by activity profiling of ten compstatin analogs of varying affinities (K_D 0.08-552 nM).

V. *In-vivo* NHP studies: Quantification of Cp60-KK in SGI.011 NHP Plasma

Pharmacokinetic Analysis in Non-Human Primates (NHPs)

Study Design and Animal Handling

Pharmacokinetic studies of Cp60-KK were conducted at the Simian Conservation Breeding and Research Center (SICONBREC) in Makati, Philippines. Two male cynomolgus monkeys (*Macaca fascicularis*), aged 6–7 years and weighing approximately 4 kg, were used. Animals were acclimatized for two weeks in sterilized ILAR type 3 stainless steel cages under controlled environmental conditions (26 ± 4 °C, 60 ± 25% humidity, natural light cycle, and proper ventilation). They were fed 100 g/day of a standard monkey grower pellet (Jetstar Milling Corp.) and had *ad libitum* access to water. Bananas were provided daily as a dietary supplement. On dosing day, food was offered post-administration.

The peptide was administered via subcutaneous (s.c) injection at a dose of 2 mg/kg. Cp60-KK (8 mg each) were dissolved in sterile saline, injections were delivered using 3/10 mL insulin syringes with 29G × ½" needles, at a dose of 2 mg/kg.

Sample Collection

Blood was drawn from the femoral vein at pre-dose (0 h) and post-dose time points: 5 min, 30 min, 1 h, 2 h, 4 h, 6 h, 12 h, 24 h, 48 h, 72 h, 96 h, and 120 h. Samples were collected in EDTA tubes, centrifuged at ~800 × g for 10 min, and plasma stored at –80 °C.

All procedures followed ethical guidelines and were approved by the Institutional Animal Care and Use Committee (Philippines). SICONBREC is accredited by AAALAC.

Determination of Cp60-KK plasma concentrations

Plasma concentrations of Cp60-KK were determined using a surface plasmon resonance (SPR)-based competition assay on a Biacore 3000 instrument (Cytiva) as described before for Cp40-KK (Hughes et al., Clin. Immunol. 214, 2020, 108391; https://doi.org/10.1016/j.clim.2020.108391). Briefly, plasma samples containing Cp60-KK samples were diluted, heat-inactivated to release C3-bound compound, and mixed with buffer containing pooled NHP plasma (time point 0) as constant source of C3. Standard curve samples containing defined concentrations of Cp60-KK were prepared in parallel. For detection, analog Cp40-KKK was immobilized on a single flow cell of a CM5 sensor chip (Cytiva) and treatment samples and standards were injected; an empty flow cell served as reference surface. Cp60-KK concentrations were determined by measuring the residual binding of free C3 to immobilized Cp40-KKK. For the analysis, buffers, standards and samples were prepares as follows:

50 mM Na_2PO_4, 100 mM NaCl, 1 mg/mL dextran sulfate, 0.02% NaN_3, 10 mM EDTA, 0.05% Tween-20, pH 7.4 was used as running buffer throughout the analysis. Buffer A was prepared by diluting 300 µL of 0-hour pooled NHP plasma with 14.7 mL running buffer (1:50 dilution) and stored on ice. Buffer B consisted of 66.7 µL of EDTA-treated human plasma diluted with 4,933 µL running buffer (1:75 dilution), also kept on ice.

For the Cp60-KK standard curve, eleven 2-mL low-bind tubes were filled with 0.5 mL Buffer A, and a twelfth with 1.5 mL. Cp60-KK (stock: 345 µM) was diluted 1:10 to 34.5 µM, then 29.3 µL was spiked into 1,471 µL Buffer A. Standards were serially diluted 1:1.5 to yield concentrations of 675 to 8 nM.

Cp60-KK standards at 89 nM and 40 nM were prepared from an 894-µM stock, diluted 1:1000 to 894 nM. For 89 nM, 49.8 µL was added to 450.2 µL Buffer A; for 40 nM, 22.4 µL was added to 477.6 µL.

Samples from animal #12422B (SGI-011; animal 1) were prepared by mixing 10 µL plasma with 490 µL running buffer. Timepoints included 5 min to 120 h. All samples were heat-inactivated at 95 °C for 5 min, cooled, and centrifuged at 14,000 rpm for 10 min. Then, 100 µL supernatant was mixed with 100 µL Buffer B for a final 1:100 dilution. Remaining samples were stored at –20 °C or lower.

Calculation of Pharmacokinetic Parameters

Plasma concentrations of Cp60-KK as determined above were plotted against time. The elimination rate constant (k_{el}) and plasma half-life ($t_{½}$) were calculated from the terminal phase (72–120 h) using:

$$\ln[C(t)] = \ln[C_0] - k_{el}\cdot t$$
$$t_{½} = \ln(2)/k_{el}$$

C_{max} and t_{max} were manually extracted from concentration-time data. The area under the curve ($AUC_{0\text{–}t}$ and $AUC_{0\text{–}\infty}$), apparent volume of distribution (Vz/F), and clearance (CL/F) were calculated using noncompartmental analysis in Phoenix WinNonlin 8.0. A linear trapezoidal rule with extravascular dosing model was applied.

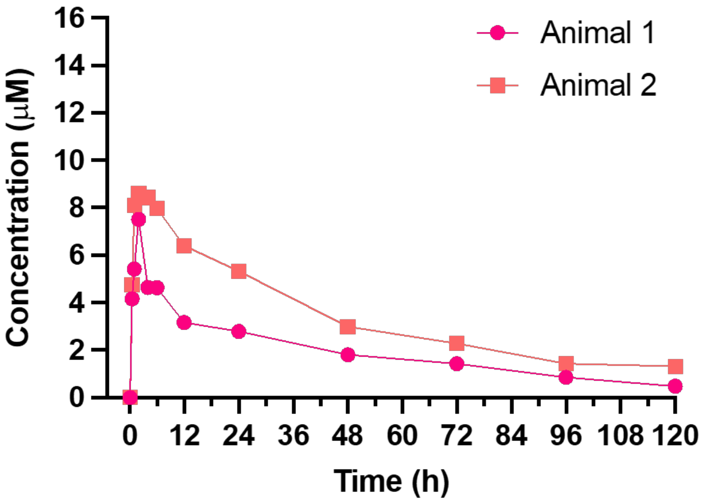

Figure S24. Quantification of peptide concentrations in the plasma of cynomolgus monkeys. Animals (n = 2) were injected subcutaneously with 2 mg/kg Cp60-KK. Peptide concentrations were determined using a SPR-based competition assay. Timepoints: 5 min, 30 min, 1 h, 2 h, 4 h, 6 h, 12 h, 24 h, 48 h, 72 h, 96 h, 120 h.

Table S1. Experimental PK parameters of Cp60-KK

Parameter	Animal 1	Animal 2
$t_{½}$ (h)	30.8	40.8
t_{max} (h)	2	2
C_{max} (μM)	7.52	8.59
$AUC_{0\text{-}120h}$ (μM h)	228	401
$AUC_{0\text{-}\infty}$ (μM h)	250	479
Vz/F (mg μM^{-1})	1.43	0.98
CL/F (mg μM^{-1} h^{-1})	0.032	0.017

VI. Aliphatic amino acid analogs at Cp01 and Cp40 position 3

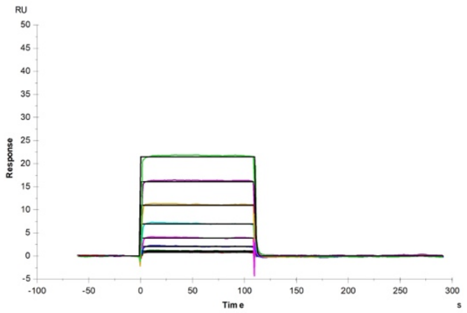

Figure S25. Representative single cycle kinetic SPR experiment of Cp01 3Dea against immobilized C3b, with serial two-fold dilutions of concentration (0.03-2 μM).

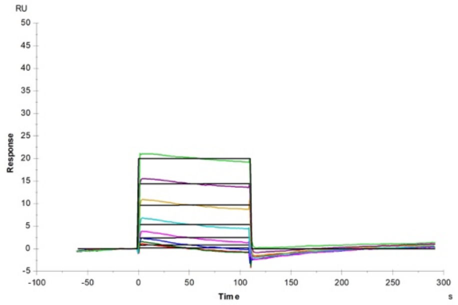

Figure S26. Representative single cycle kinetic SPR experiment of Cp01 3aIle against immobilized C3b, with serial two-fold dilutions of concentration (0.03-2 μM).

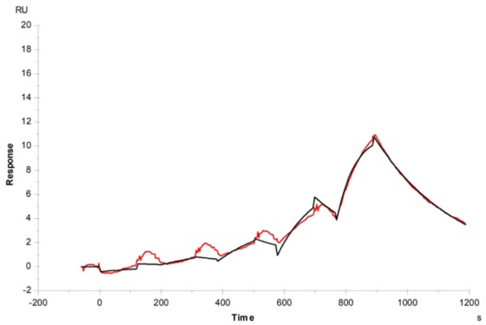

Figure S27. Representative single cycle kinetic SPR experiment of Cp40 3Dea against immobilized C3b, with serial two-fold dilutions of concentration (0.5-40 nM).

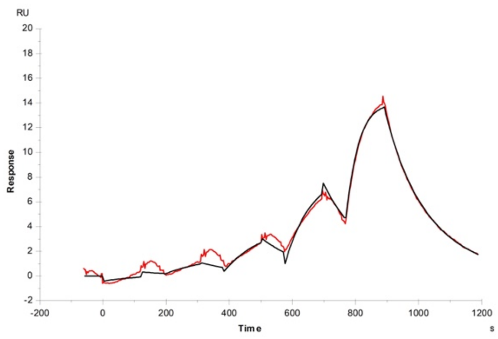

Figure S28. Representative single cycle kinetic SPR experiment of Cp40 3alle against immobilized C3b, with serial two-fold dilutions of concentration (0.5-40 nM).

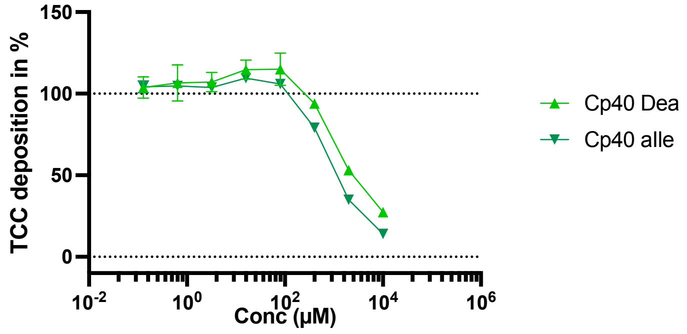

Figure S29. Inhibition curves of Cp40 3Dea and Cp40 3alle in classical pathway complement activation ELISA with 1% normal human serum.

Alternative pathway

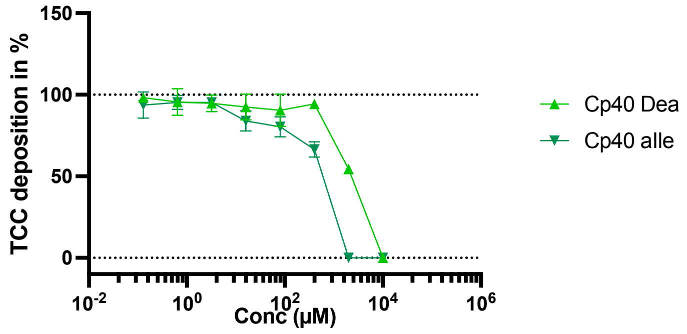

Figure S30. Inhibition curves of Cp40 3Dea and Cp40 3alle in alternative pathway complement activation ELISA with 10% normal human serum.

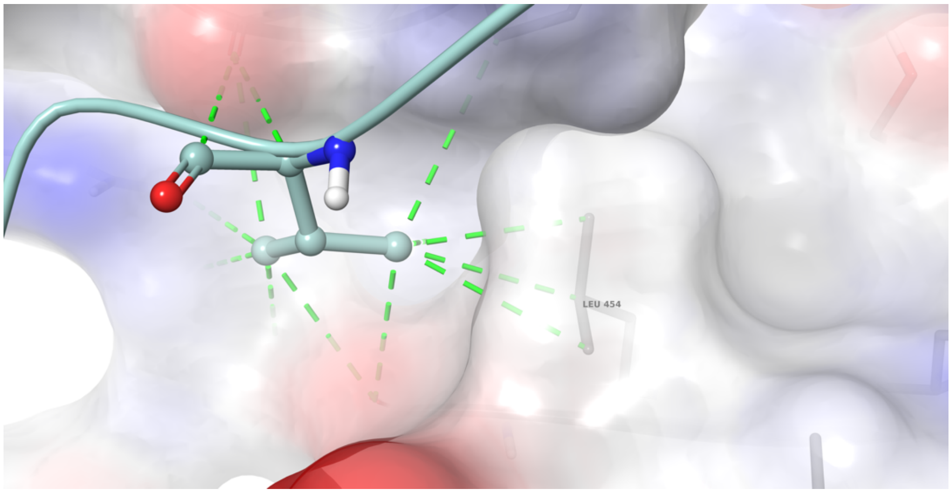

Figure S31. Structural visualization of Cp01-Val3 interaction with C3b Leu454, derived from the X-ray crystal structure (PDB: 2QKI).

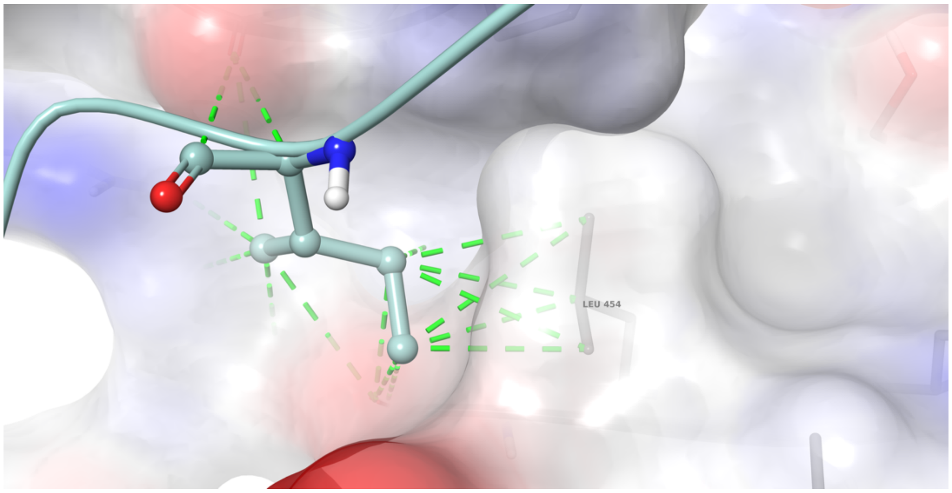

Figure S32. Structural visualization of Cp01-Val3Ile interaction with C3b Leu454, derived from the predicted model *in silico*.

VII. Cryo-EM analysis of Cp60-KK-C3bB complex

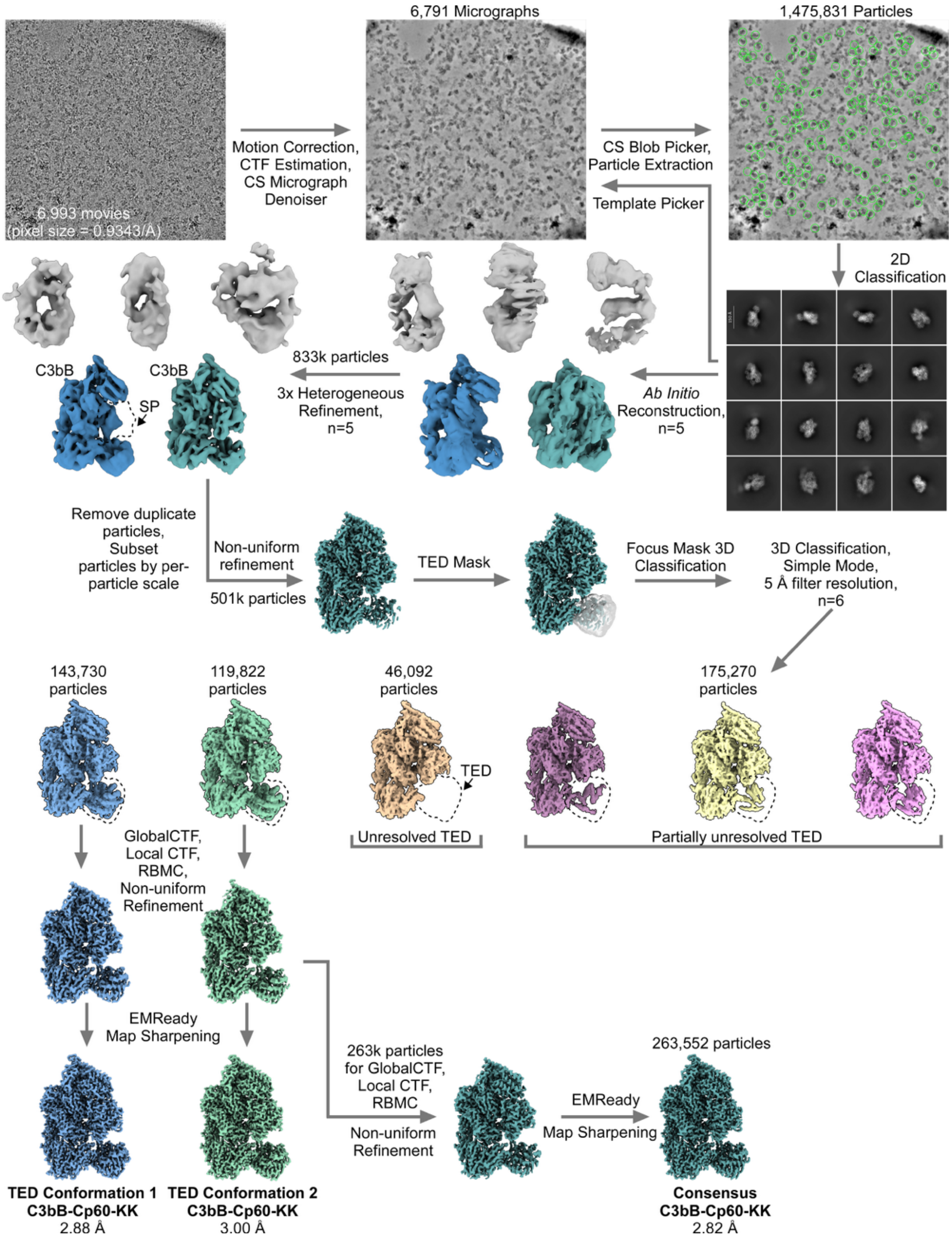

Figure S33. Cryo-EM data processing workflow for C3bB-Cp60-KK
Cryo-EM micrograph preprocessing pipeline with representative micrographs, 2D classes, and 3D classes of inhibitor bound C3bB particles. Maps colored in gray represent “junk” particles and were discarded from downstream analysis. Final cryo-EM density maps obtained by masked 3D classification and non-uniform refinement revealed two major conformations of the pro-convertase inhibited by Cp60-KK differing only by movement of the TED with resolutions of 2.88 Å and 3.01 Å, respectively. Combining these particles resulted in a high-resolution structure of C3bB-Cp60-KK reaching 2.82 Å

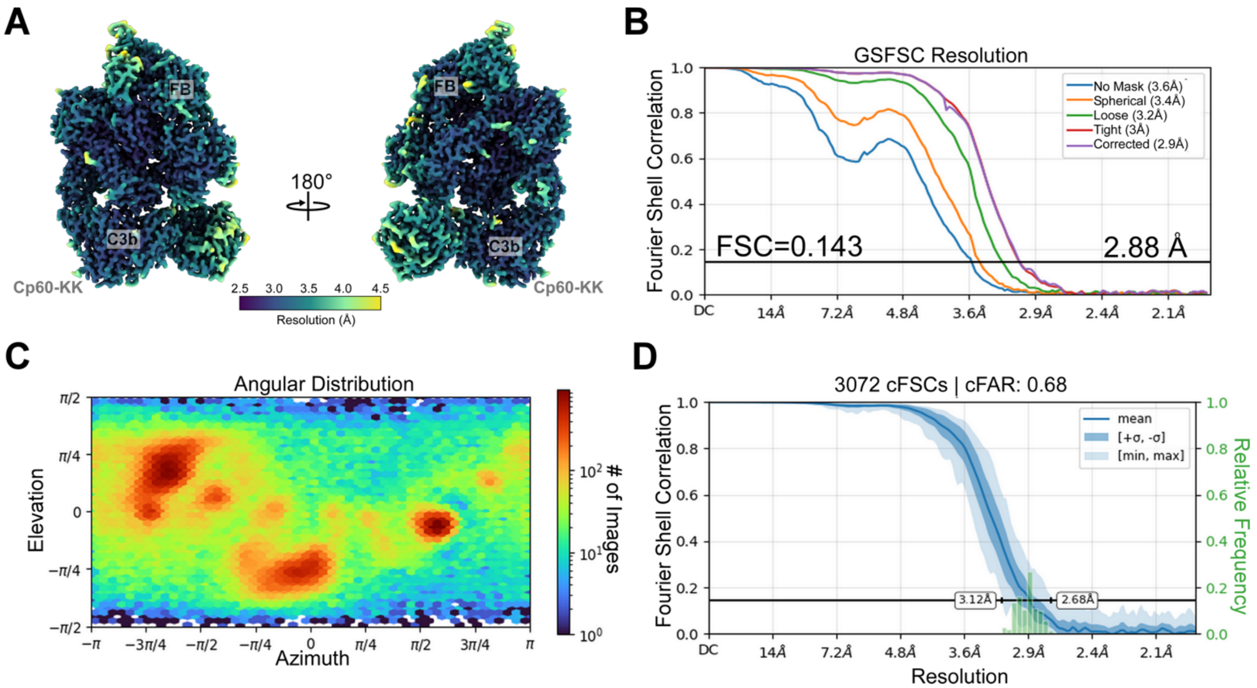

Figure S34. Cryo-EM quality assessment of C3bB-Cp60-KK TED Conformation 1

(A) Local resolution estimation of C3bB-Cp60-KK. **(B)** GS-FSC curves from CryoSPARC using the gold standard FSC threshold (FSC threshold 0.143). **(C)** Distribution of viewing angles and orientations for the C3bB-Cp60-KK cryo-EM density map. **(D)** Orientation diagnostics of C3bB-Cp60-KK using the conical FSC area ratio (cFAR).

Table S2. Cryo-EM data collection, refinement, and validation statistics of C3bB-Cp60-KK

	C3bB-Cp60-KK Complex	
Data Collection		
Electron Microscope	Glacios 2	
Voltage (kV)	200	
Detector	Falcon 4i	
Camera mode	Counting	
Magnification	130,000x	
Total electron dose (e- Å^{-2})	43.36	
Defocus range (- μm)	0.3-2.1	
Pixel Size (Å)	0.9343	
Grid type	Quantifoil® R1.2/1.3 300 Mesh, Cu	
Software	EPU	
Dose rate (e- Å^{-2} s^{-1})	7.23	
Frames (no.)	40 (1845 EER fractions)	
Energy Filter	Selectris 20 eV	
Micrographs collected (no.) & curated (no.)	6,993 (6,791)	
Image Processing		
SPA Software	CryoSPARC 4.7.1	
Symmetry imposed	C1	
Initial particles picked (no.)	2,440,308	
Particles after 2D classification (no.)	1,475,831	
Particles after ab initio reconstruction (no.)	833,935	
Particles after Remove Duplicates (no.)	732,106	
Models	**C3bB-Cp60-KK TED 1**	**C3bB-Cp60-KK TED 2**
Particles after heterogeneous refinement (no.)	501,139	
Particles after 3D Classification	263,552	
Final particles picked (no.)	143,730	119,822
Map Resolution (Å)	2.88	3.00
FSC Threshold	0.143	0.143
Refinement		
Initial model used (PDB)	2XWJ	2XWJ
Model composition		
Nonhydrogen atoms	18107	18083
Protein residues	2275	2274
Water	0	0
Ligands	DTY: 1<br>EXL:1<br>IML:1<br>SAR:1<br>NAG: 7<br>Ni:2	DTY: 1<br>EXL:1<br>IML:1<br>SAR:1<br>NAG: 7<br>Ni:2
B factors (Å^2) (min/max/mean)		
Protein	28.15/159.49/50.95	24.44/167.37/51.51
Ligands	37.58/168.37/88.46	40.97/130.53/84.93
R.m.s. deviations		
Bond lengths (Å) (# > 4σ)	0.004 (0)	0.004 (0)
Bond angles (º) (# > 4σ)	0.800 (1)	0.806 (8)
Validation		
MolProbity score	1.14	1.40
Clash Score	2.83	3.78
Poor rotamers (%)	0.55	1.5
Ramachandran Plot		
Preferred (%)	97.69	97.51
Allowed (%)	2.31	2.49
Outliers (%)	0.00	0.00
Map-to-Model fit (CC_{Mask})	0.91	0.90

VIII. Application of biotinylated Cp60 for C3 detection in ELISA

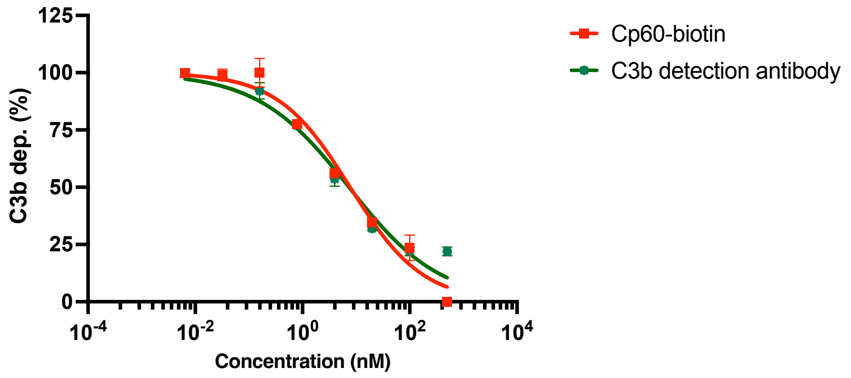

Figure S35. Biotinylated Cp60 as detection tool in lectin pathway ELISA.
Comparative ELISA showing dose-dependent inhibition of the lectin pathway by an anti-MASP-2 mAb (narsoplimab surrogate; MedChemExpress); C3-fragment deposition was either detected with biotinylated Cp60, followed by HRP-NeutrAvidin, or an anti-C3b/iC3b mAb (clone 3E7, Biolegend), followed by an alkaline phosphatase-conjugated secondary antibody.

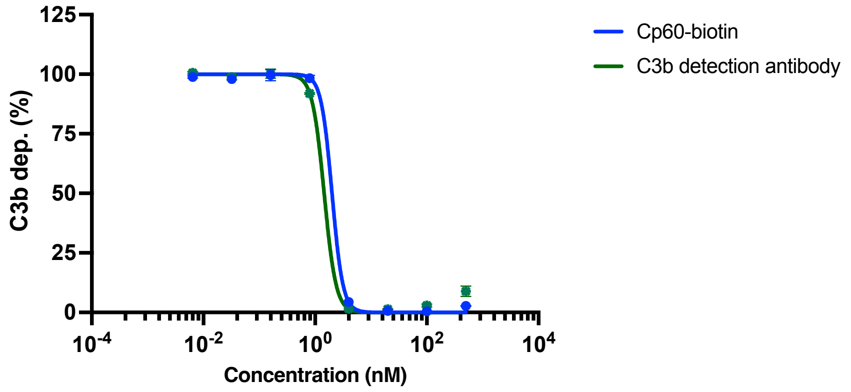

Figure S36. Biotinylated Cp60 as detection tool in classical pathway ELISA.
Comparative ELISA showing dose-dependent inhibition of the classical pathway by an anti-C1s mAb (sutimlimab surrogate; MedChemExpress); C3-fragment deposition was either detected with biotinylated Cp60, followed by HRP-NeutrAvidin, or an anti-C3b/iC3b mAb (clone 3E7, Biolegend), followed by an alkaline phosphatase-conjugated secondary antibody.

IX. Comparison of experimental C3bB structures

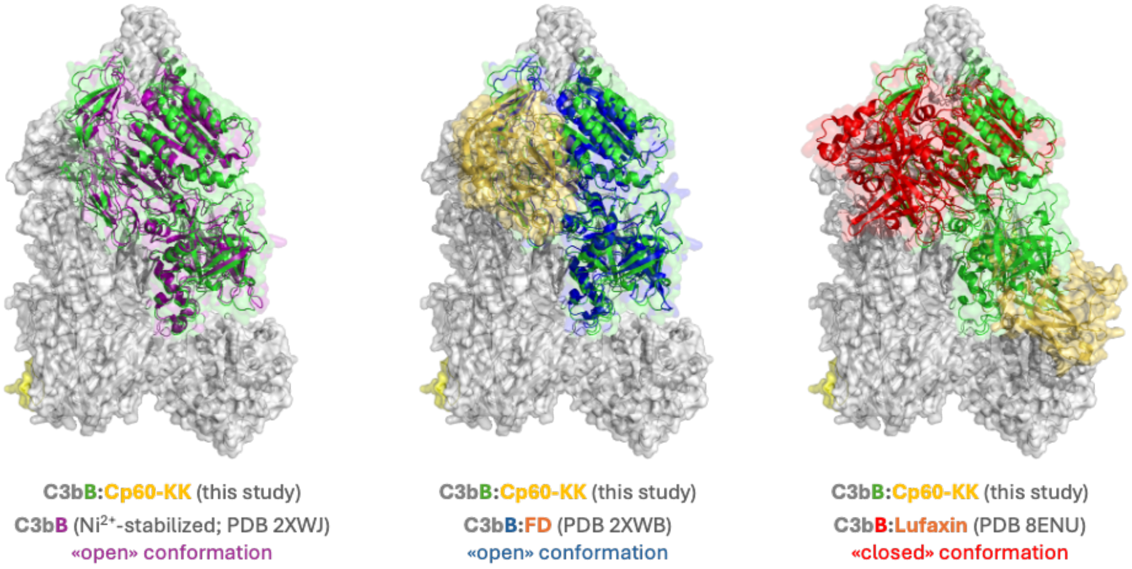

Figure S37. Overlay of the C3bB:Cp60-KK structure resolved in this work with previously reported C3bB structures.
Differential conformations of bound factor B "**B**" corresponding to "open" or "closed" states. C3b chain is indicated as grey surface, whereases the domains corresponding to factor B are colored accordingly. Structural overlay and visualization was conducted in PyMOL (Schrödinger, v3.1.6.1).

X. Supplementary materials and methods

Materials and instruments

Unless otherwise stated, chemicals and solvents were purchased from commercial suppliers (Sigma Aldrich, Fluorochem, ChemImpex, VWR, CombiBlocks, Roth and Fisher Scientific) and used without further purification. HPLC grade (>99.8%) dimethylformamide (DMF) was used for peptide synthesis. Preparative HPLC-MS was carried out using an Agilent 1260 Infinity II preparative HPLC system coupled to an on-line Agilent LC/MSD XT ESI+ module. Analytical HPLC was conducted using an Agilent 1100 system. All HPLC systems used UV analyte detection at 214 nm and 280 nm. Automated SPPS was performed using a Liberty Blue microwave peptide synthesizer (CEM corp.). Peptide lyophilization was carried out by flash freezing the sample in liquid N_2 and drying on a Christ Alpha 2-4 LDplus freeze-dryer. SPR experiments were conducted on a Biacore T200 instrument. ELISAs were analyzed by a Synergy HT plate reader. Flow cytometry experiments were conducted on a CytoFlex B4-R3-V0 (Beckman Coulter).

Peptide synthesis

Automated solid-phase peptide synthesis

Automated SPPS was utilized in the assembly of the compstatin analogs. Synthesis was conducted at 0.1 mmol scale using rink amide MBHA resin (0.71 mmol/g, Novabiochem). Fmoc-amino acid stock solutions were used at 0.2 M concentration, Oxyma at 1.0 M concentration and DIC at 0.5 M concentration. Reactions were stirred by N_2 bubbling for 2 seconds on, 3 seconds off.

Fmoc deprotection: 3 mL of piperazine (10% w/v) in NMP:EtOH (1:9) was delivered to the reaction vessel. Microwave heating proceeded as follows: 0 W 20 ± 5 °C for 5 s, 100 W 78 ± 2 °C for 20 s, 60 W 88 ± 2 °C for 10 s, 20 W 90 ± 1 °C for 60 s. The resin was then washed with DMF (4 x 4 mL).

Coupling cycle: Fmoc-amino acid (2.5 mL, 5 equiv.), Oxyma (0.25 mL, 5 equiv.) and DIC (0.5 mL, 5 equiv.) were delivered to the reaction vessel (final concentrations: Amino acid 125 mM, Oxyma 125 mM, DIC 125 mM). Microwave heating proceeded as follows: 15 W at 75 ± 2 °C for 15 s, 30 W at 90 ± 1 °C for 225 s. The resin was then washed once with DMF (4 mL).

Following assembly of the respective compstatin sequence (with the exception of Cp40 derivatives), the N-terminus of the peptide was acetylated in the peptide synthesizer by the addition of 6 mL of acetic anhydride (10% v/v) in DMF, followed by microwave heating: 40 W at 65 ± 5 °C for 35 s, 0 W at 65 ± 5 °C for 35 s, 40 W at 65 ± 5 °C for 35 s, 0 W at 65 ± 5 °C for 35 s. The resin was then washed five times with DMF (4 mL).

Peptide cleavage

Peptide resins were washed with DCM (4 x 4 mL) and allowed to air dry for 15 min. Cleavage of the peptide from solid support and deprotection of remaining side chain-protected groups was achieved by addition of a peptide cleavage cocktail (TFA:H_2O:DODT:TIS, 90:5:2.5:2.5; 1 mL per 20 μmol resin) and allowed to react for 1 h at room temperature. The TFA-cocktail containing the peptide was drained into a 50-mL centrifuge tube and concentrated under a stream of inert gas. The peptide was subsequently precipitated by addition of ice-cold Et_2O (40 mL), and pelleted by centrifugation for 5 min at 3500 RCF. The peptide was washed twice with Et_2O, collected by centrifugation, dissolved in water/acetonitrile +0.1% TFA, and lyophilized to yield the crude linear peptide.

Peptide cyclization

Crude compstatin peptide (approx. 0.1 mmol) was dissolved in 30% aqueous acetonitrile (100 mL) in a 250-mL round-bottomed flask equipped with a magnetic stirrer bar. The pH was adjusted to approx. 8 using 5% (v/v) ammonium hydroxide, followed by addition of hydrogen peroxide (0.3 mmol). The solution was stirred for 30 min at room temperature, and reaction completion was confirmed by positive electrospray ionization mass spectrometry (ESI+ MS). Then, the reaction was quenched by adjusting to pH 2-4 with TFA and the solution was lyophilized to yield the crude cyclic peptide.

Synthesis of sulfo-cyanine 5 labelled compstatins

Compstatin analogs were prepared by automated SPPS as indicated above, containing an additional Fmoc-Lys(ivDde)-OH amino acid (Combi-blocks) at the *C*-terminus. For Cp01 and Cp01 V3I, the *N*-terminus of the peptide was acetylated as described. For Cp40 and Cp60, the *N*-terminal D-tyrosine was installed using the Boc-D-Tyr(*t*Bu)-OH (Fluorochem) building block. The resin (20 μmol equivalent) was transferred to a 5-mL peptide synthesis vessel fitted with a PTFE frit (Roth). The ivDde protecting group was removed by the addition of hydrazine (2 mL, 5% v/v in DMF) for 30 min. The solution was drained and the deprotection step repeated once. The resin was washed with DMF (5 x 3 mL). Two equivalents (40 μmol) of sulfo-cyanine 5 carboxylic acid (sCy5; MedChemExpress, HY-D0821) were dissolved in DMF (0.5 mL). HATU (38 μmol) and DIPEA (80 μmol) was added to this solution and vortexed to dissolve. The solution was transferred to the resin and allowed to react at 50 °C for 2 h in the dark. The resin was then drained and washed 5 times with DMF and 5 times with DCM, and subjected to cleavage and cyclization (using 30 μmol H_2O_2) as described above.

Preparative HPLC-MS

Crude lyophilized cyclic peptides were dissolved in a minimum volume of water (+0.1% TFA) and up to 30% v/v MeCN (+0.1% TFA). The samples were then passed through a 0.2-μM filter. 0.5-3.6 mL of sample was eluted with reversed mobile phase A (water + 0.1% TFA) and B (acetonitrile + 0.1% TFA) at 15 mL/min over a RP-C18 column (Waters Xselect CSH OBD, 19 x 250 mm, 130 Å, 5 μm) at room temperature. A gradient of A/B was applied over 24 min (generally, 25-45%); analytes were detected by UV at 214 and 280 nm, and identities confirmed by online ESI+ MS. Sample fractions were collected using an automated fraction collector, and fractions containing the target peptide were combined and lyophilized. The purity of isolated peptides was determined by analytical HPLC as described in the experimental section.

XI. Peptide HPLC chromatograms and ESI+ mass spectra

Cp01

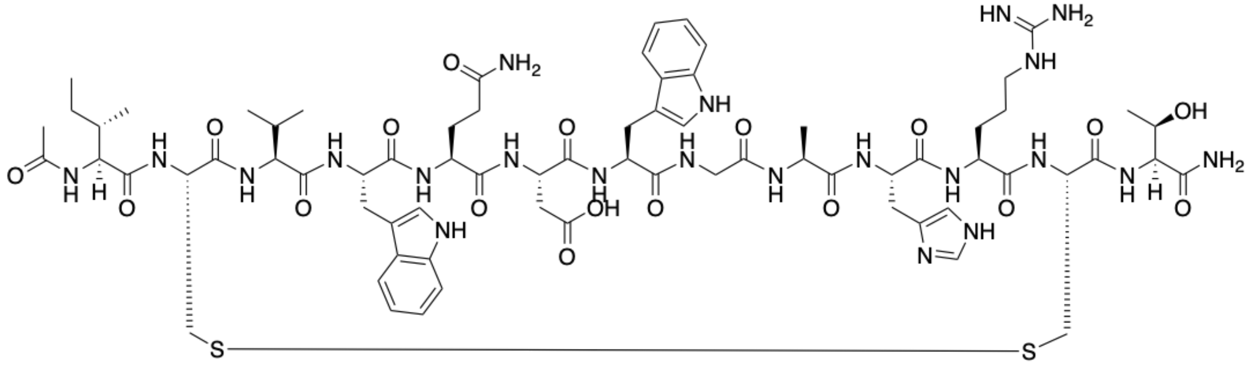

Exact Mass: 1612.70
Molecular Weight: 1613.84

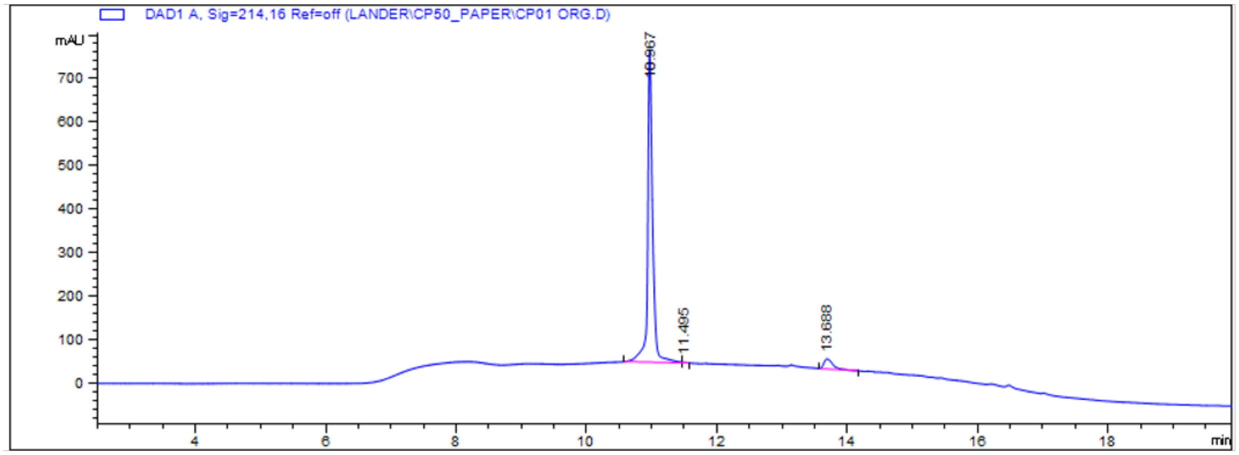

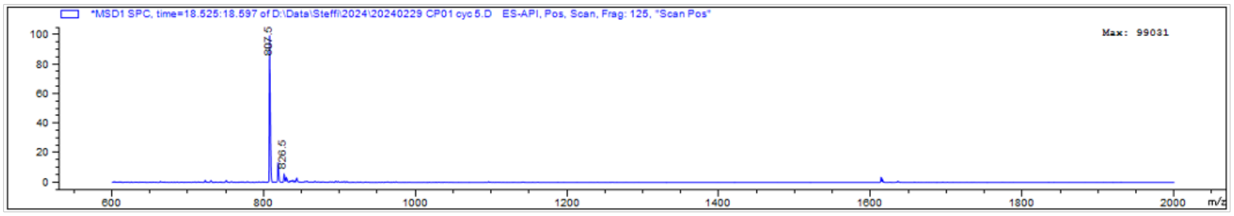

Figure S38. Chemical structure, calculated masses, HPLC chromatogram (214 nm) and ESI+ mass spectrum of isolated **Cp01**. Analytical HPLC purity: 95%, ESI+ MS (m/z): calculated 807.4 $[M+2H]^{2+}$, observed 807.5 $[M+2H]^{2+}$.

Cp01 V3A

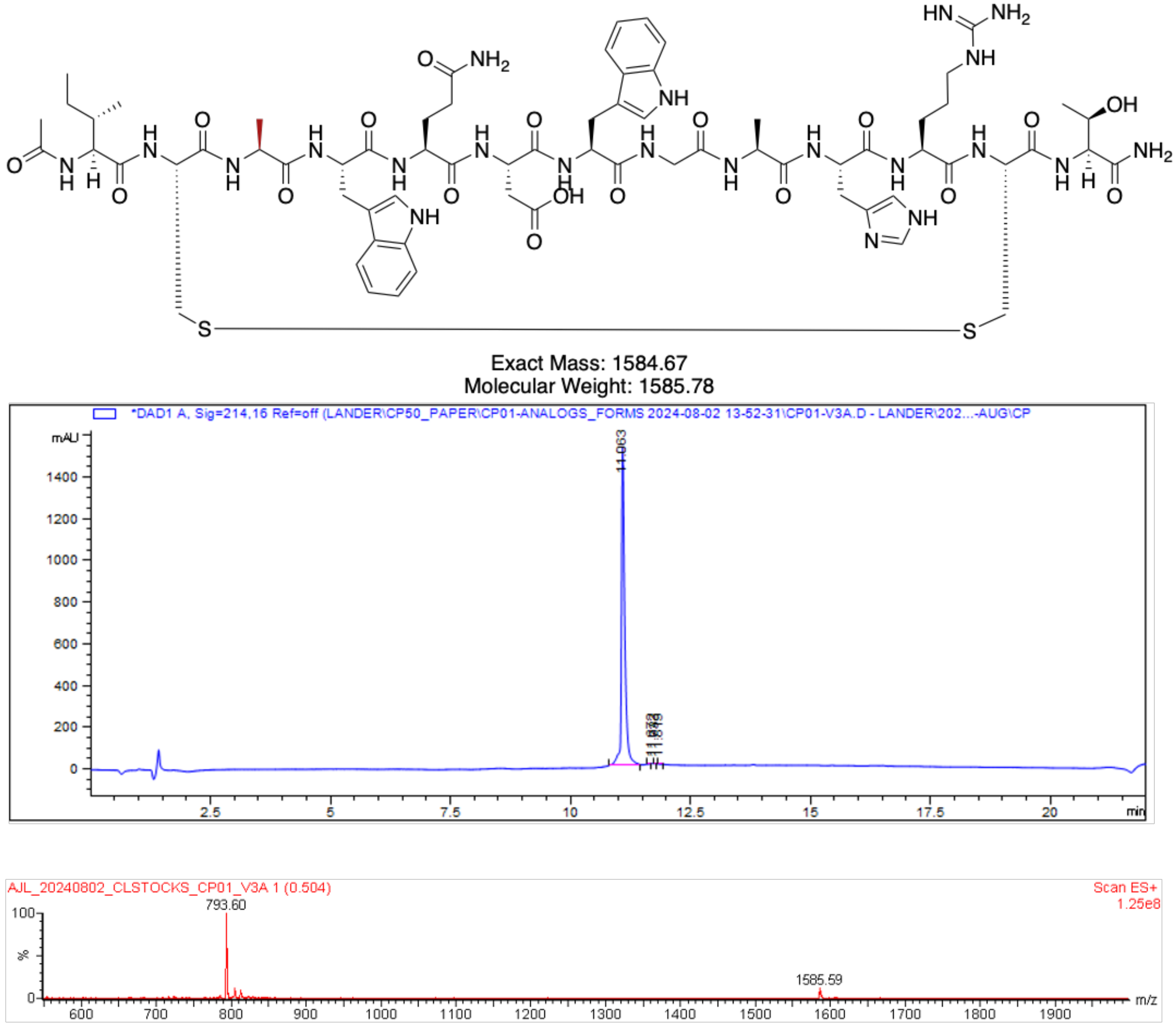

Figure S39. Chemical structure, calculated masses, HPLC chromatogram (214 nm) and ESI+ mass spectrum of isolated **Cp01 V3A**. Analytical HPLC purity: >98%, ESI+ MS (m/z): calculated 793.4 $[M+2H]^{2+}$, 1585.7 $[M+H]^{+}$, observed 793.6 $[M+2H]^{2+}$, 1585.6 $[M+H]^{+}$.

Cp01 V3L

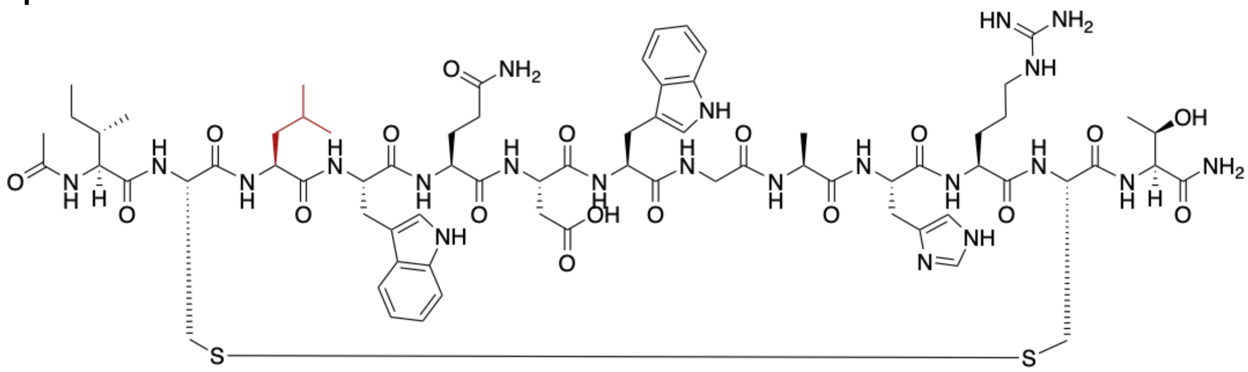

Exact Mass: 1626.72
Molecular Weight: 1627.86

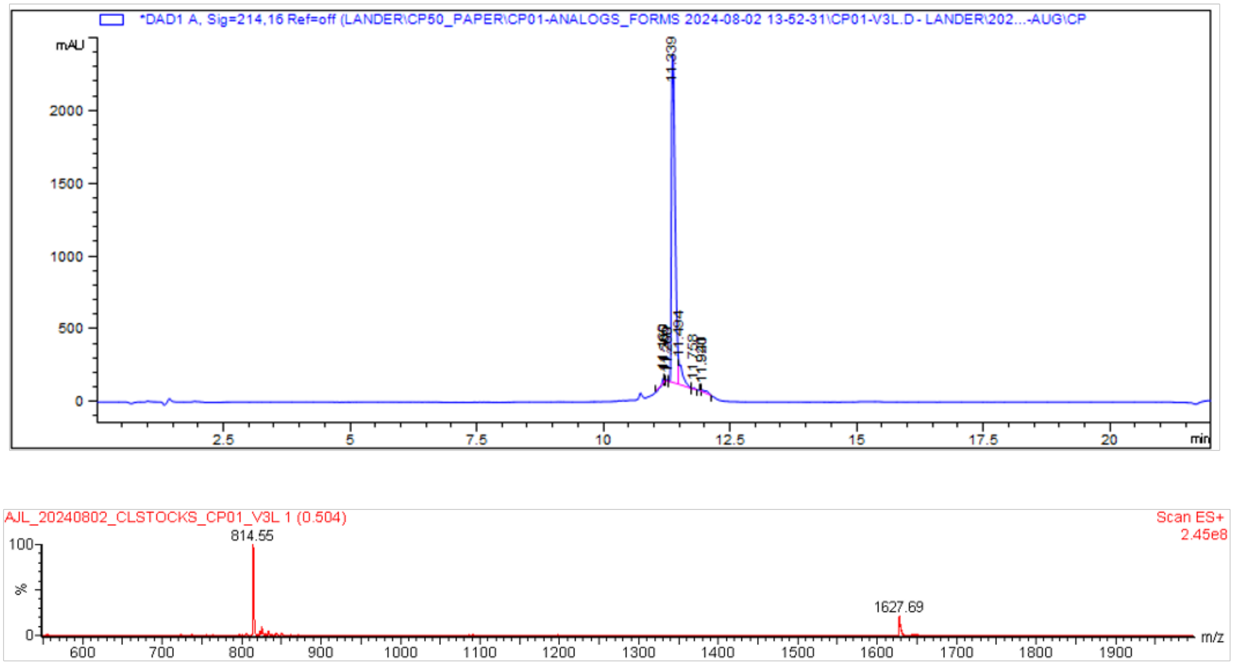

Figure S40. Chemical structure, calculated masses, HPLC chromatogram (214 nm) and ESI+ mass spectrum of isolated **Cp01 V3L.** Analytical HPLC purity: 92%, ESI+ MS (m/z): calculated 814.4 $[M+2H]^{2+}$, 1627.7 $[M+H]^{+}$, observed 814.6 $[M+2H]^{2+}$, 1627.7 $[M+H]^{+}$.

Cp01 V3Aib

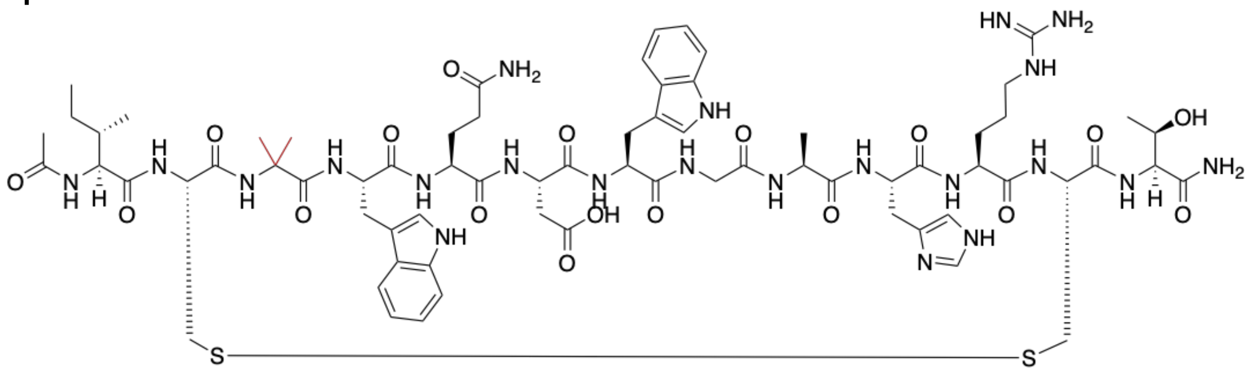

Exact Mass: 1598.69
Molecular Weight: 1599.81

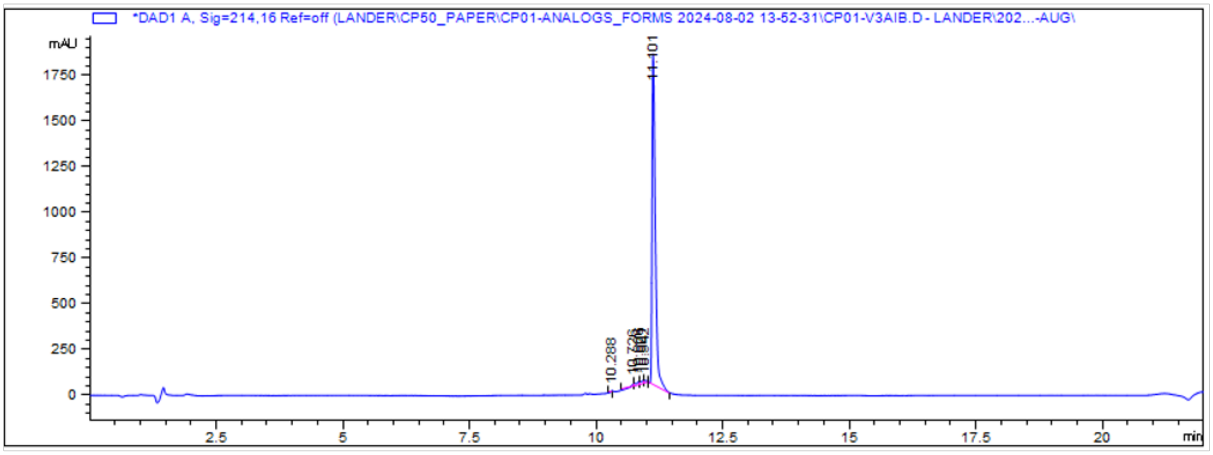

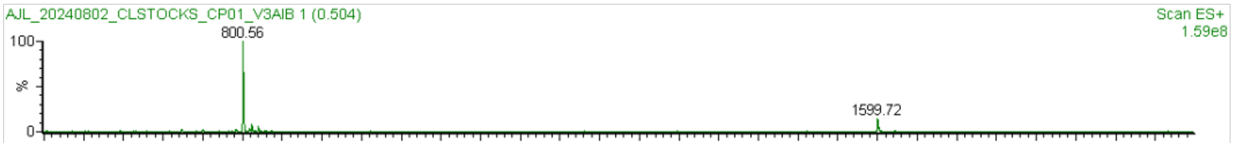

Figure S41. Chemical structure, calculated masses, HPLC chromatogram (214 nm) and ESI+ mass spectrum of isolated **Cp01 V3Aib**. Analytical HPLC purity: 95%, ESI+ MS (m/z): calculated 800.4 $[M+2H]^{2+}$, 1599.7 $[M+H]^{+}$, observed 800.6 $[M+2H]^{2+}$, 1599.7 $[M+H]^{+}$.

Cp01 V3Tbg

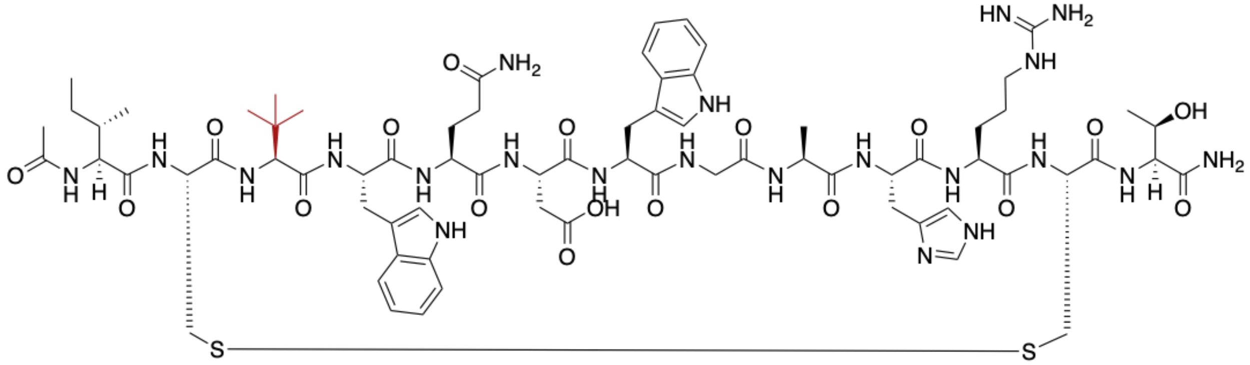

Exact Mass: 1626.72
Molecular Weight: 1627.86

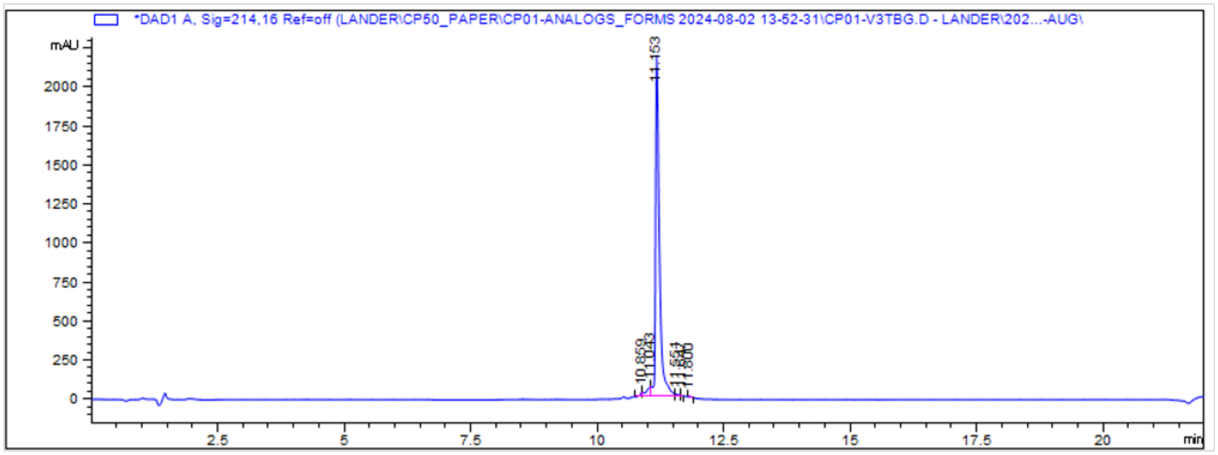

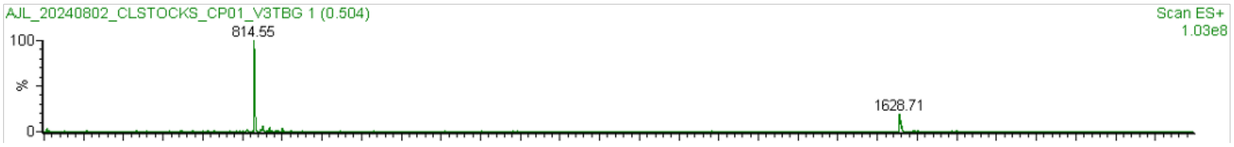

Figure S42. Chemical structure, calculated masses, HPLC chromatogram (214 nm) and ESI+ mass spectrum of isolated **Cp01 V3Tbg**. Analytical HPLC purity: 95%, ESI+ MS (m/z): calculated 814.4 $[M+2H]^{2+}$, 1627.7 $[M+H]^{+}$, observed 814.6 $[M+2H]^{2+}$, 1628.7 $[M+H]^{+}$.

Cp01 V3W

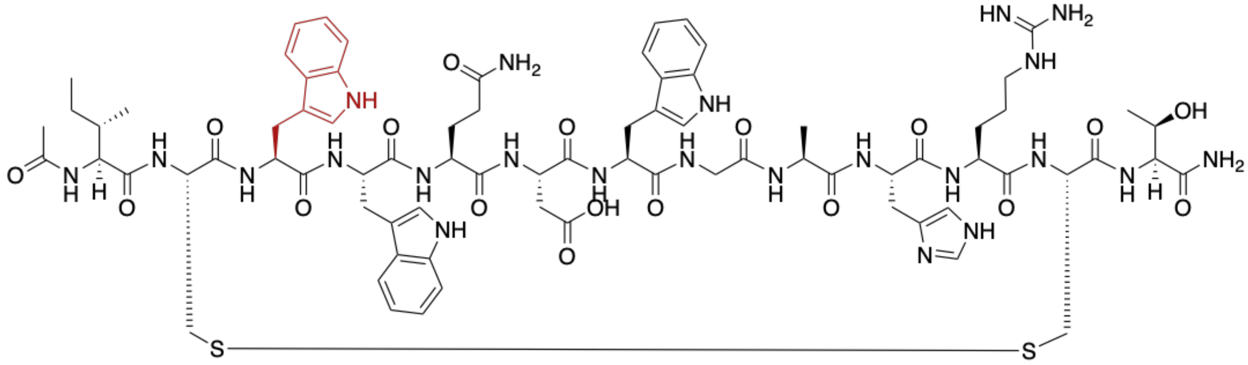

Exact Mass: 1699.71
Molecular Weight: 1700.92

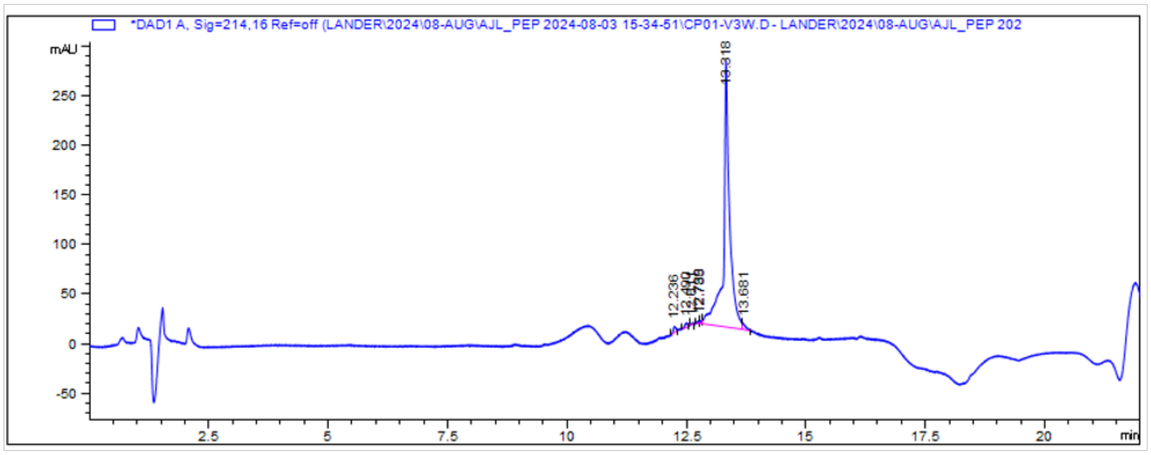

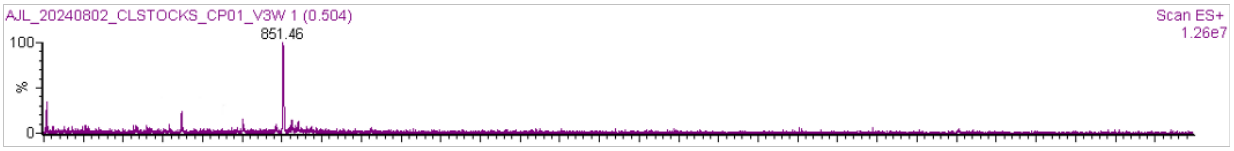

Figure S43. Chemical structure, calculated masses, HPLC chromatogram (214 nm) and ESI+ mass spectrum of isolated **Cp01 V3W**. Analytical HPLC purity: >90%, ESI+ MS (m/z): calculated 850.9 $[M+2H]^{2+}$, observed 851.5 $[M+2H]^{2+}$.

Cp01 V3F

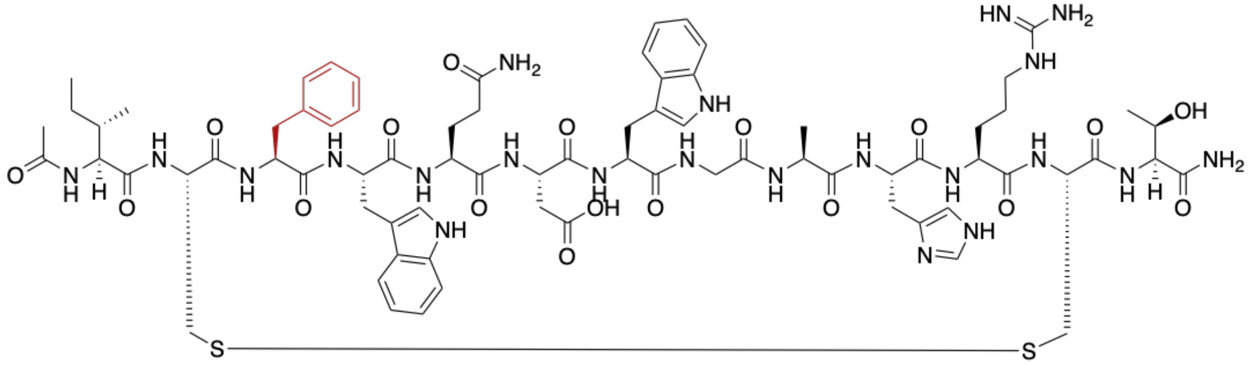

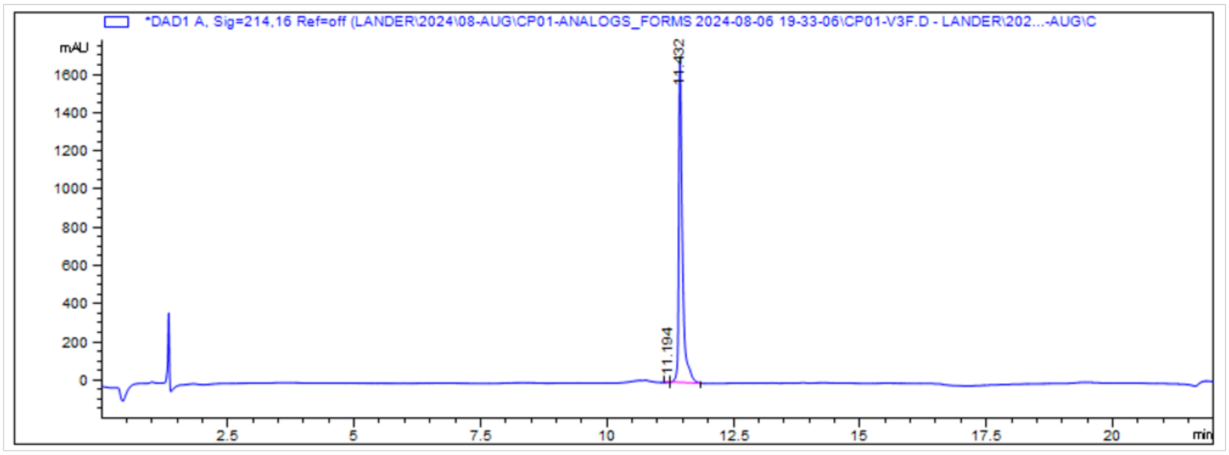

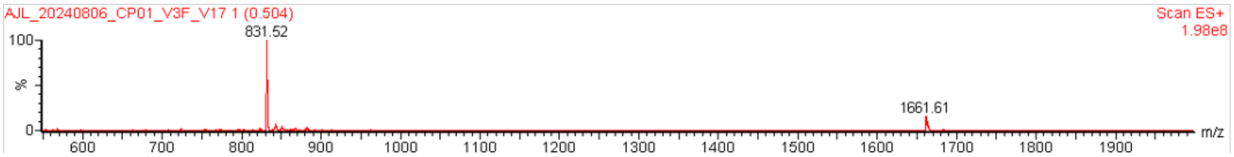

Figure S44. Chemical structure, calculated masses, HPLC chromatogram (214 nm) and ESI+ mass spectrum of isolated **Cp01 V3F**. Analytical HPLC purity: >98%, ESI+ MS (m/z): calculated 831.4 $[M+2H]^{2+}$, 1661.7 $[M+H]^{+}$, observed 831.5 $[M+2H]^{2+}$, 1661.6 $[M+H]^{+}$.

Cp01 V3Y

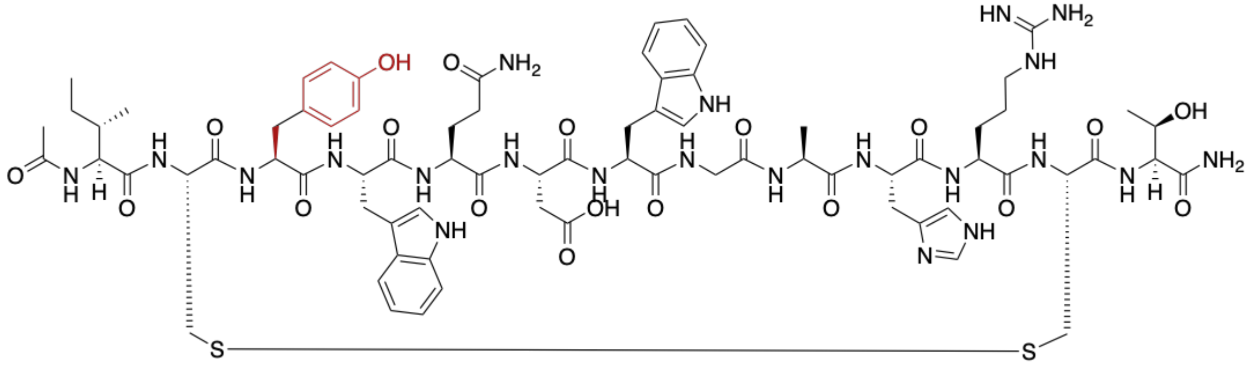

Exact Mass: 1676.70
Molecular Weight: 1677.88

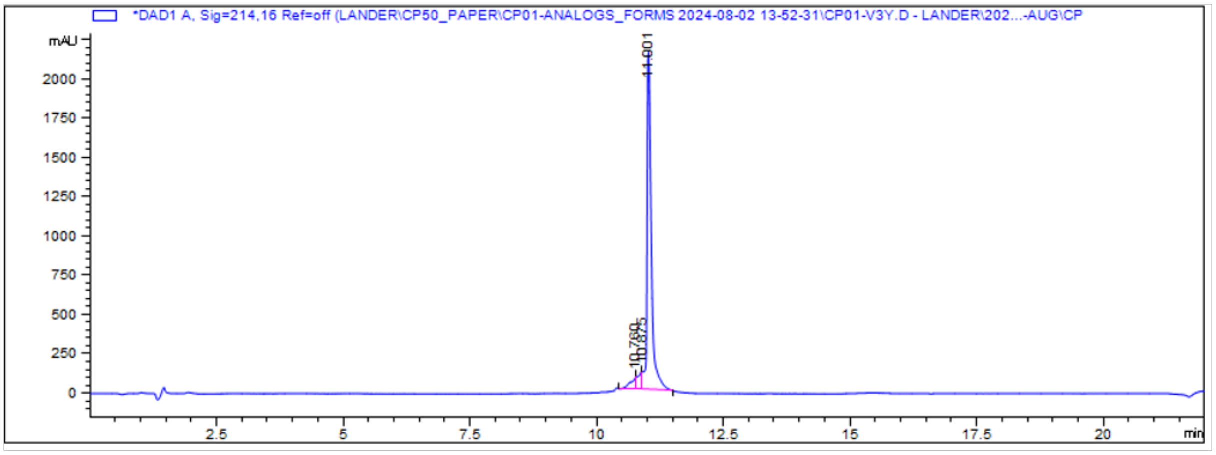

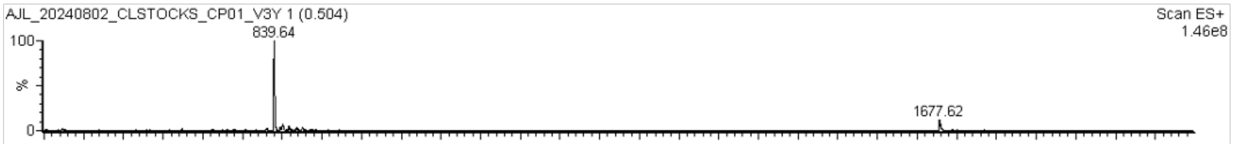

Figure S45. Chemical structure, calculated masses, HPLC chromatogram (214 nm) and ESI+ mass spectrum of isolated **Cp01 V3Y**. Analytical HPLC purity: 91%, ESI+ MS (m/z): calculated 839.4 $[M+2H]^{2+}$, 1677.7 $[M+H]^{+}$, observed 839.6 $[M+2H]^{2+}$, 1677.6 $[M+H]^{+}$.

Cp01 V3I

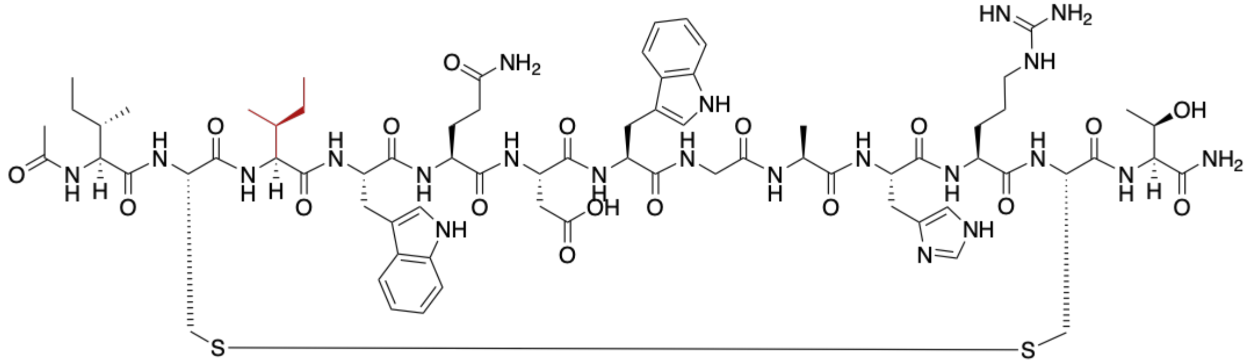

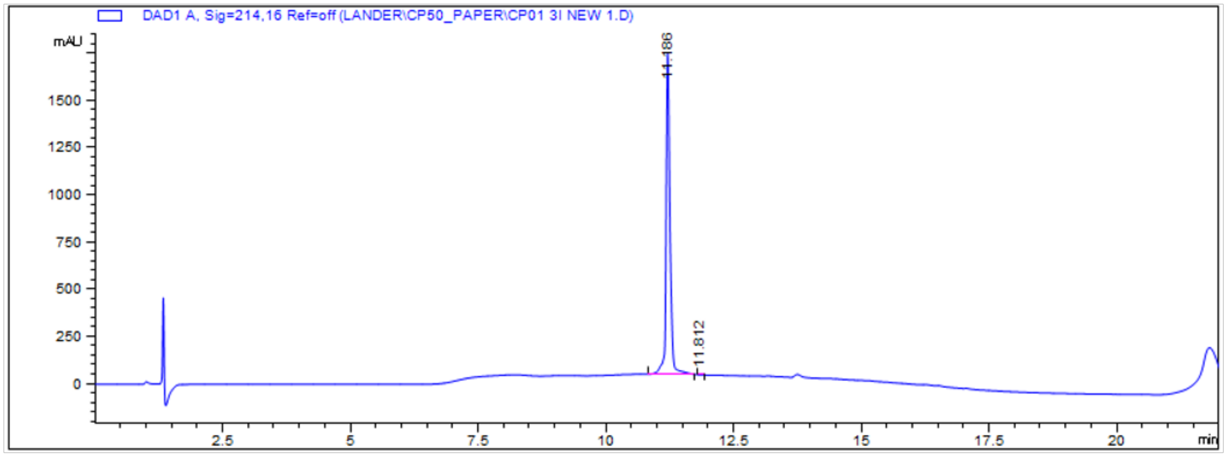

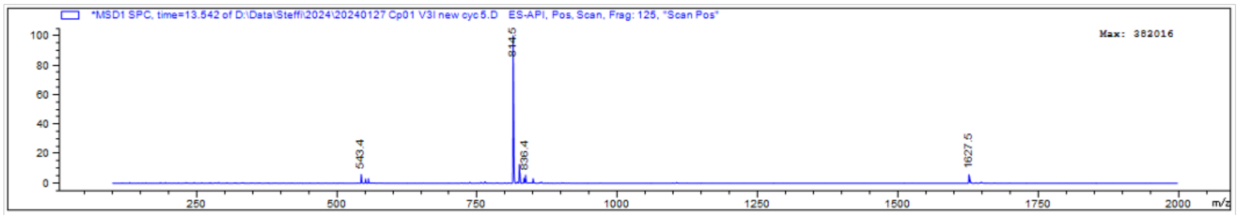

Figure S46. Chemical structure, calculated masses, HPLC chromatogram (214 nm) and ESI+ mass spectrum of isolated **Cp01 V3I**. Analytical HPLC purity: >98%, ESI+ MS (m/z): calculated 543.2 $[M+3H]^{3+}$, 814.4 $[M+2H]^{2+}$, 1627.7 $[M+H]^{+}$, observed 543.4 $[M+3H]^{3+}$, 814.5 $[M+2H]^{2+}$, 1627.5 $[M+H]^{+}$.

Cp01 V3Abu

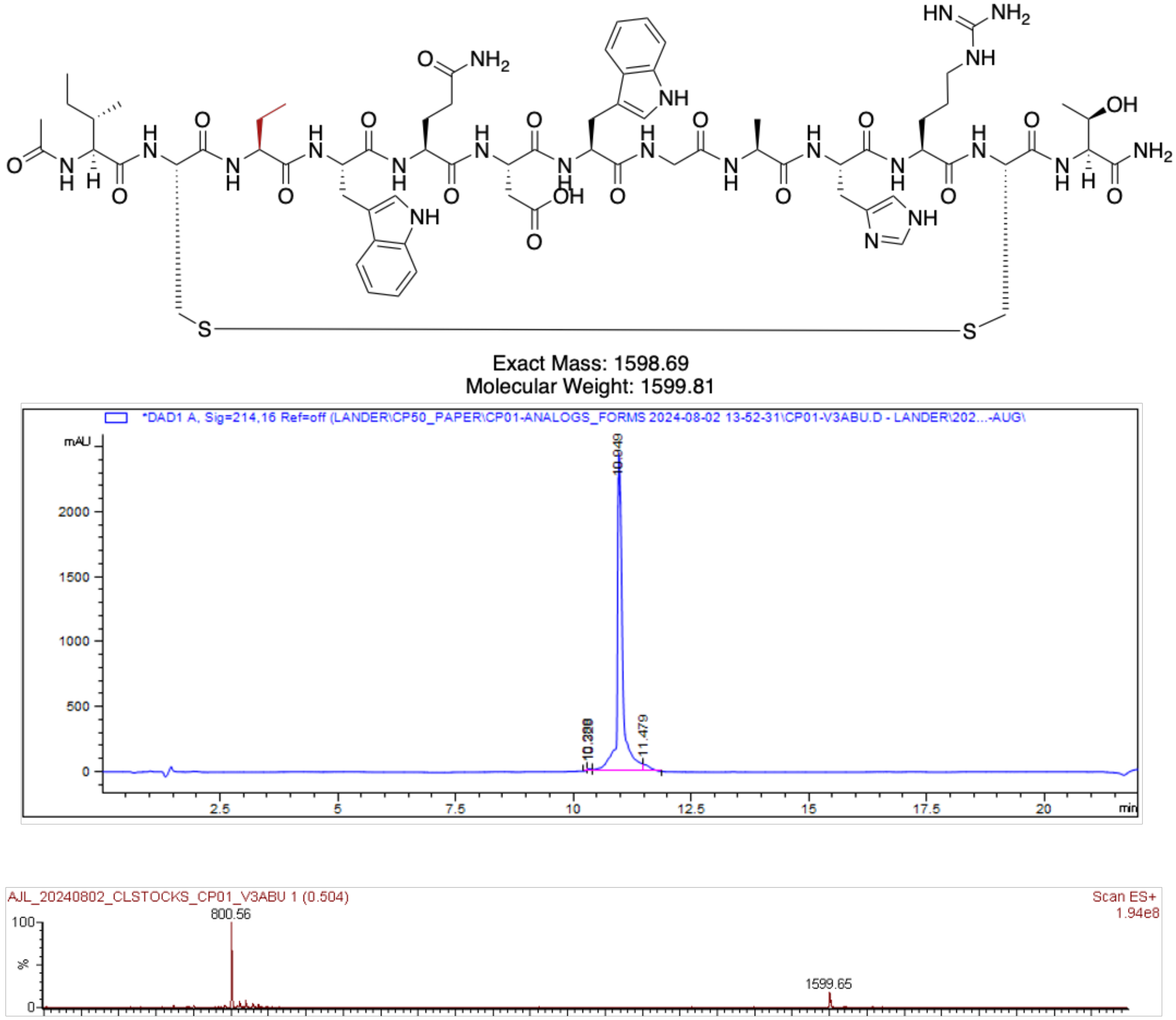

Figure S47. Chemical structure, calculated masses, HPLC chromatogram (214 nm) and ESI+ mass spectrum of isolated **Cp01 V3Abu**. Analytical HPLC purity: 96%, ESI+ MS (m/z): calculated 800.4 $[M+2H]^{2+}$, 1599.7 $[M+H]^{+}$, observed 800.6 $[M+2H]^{2+}$, 1599.7 $[M+H]^{+}$.

Cp01 V3Nva

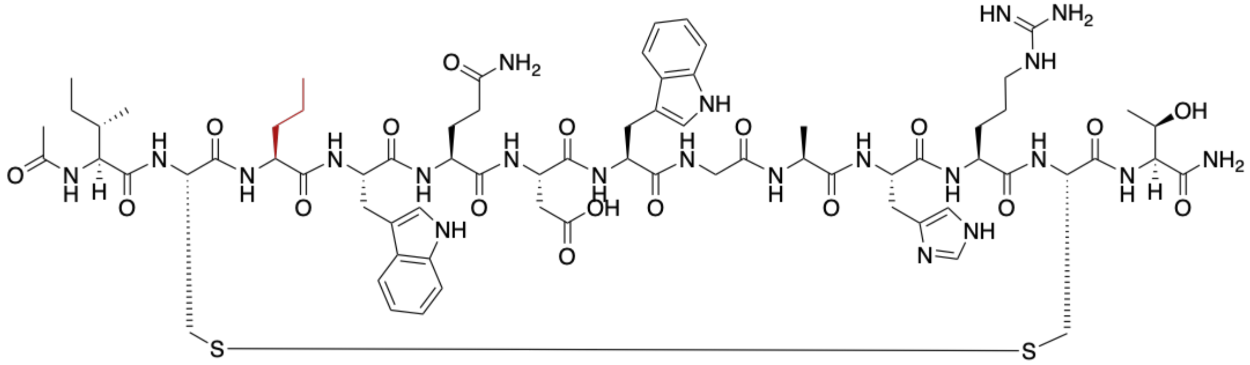

Exact Mass: 1612.70
Molecular Weight: 1613.84

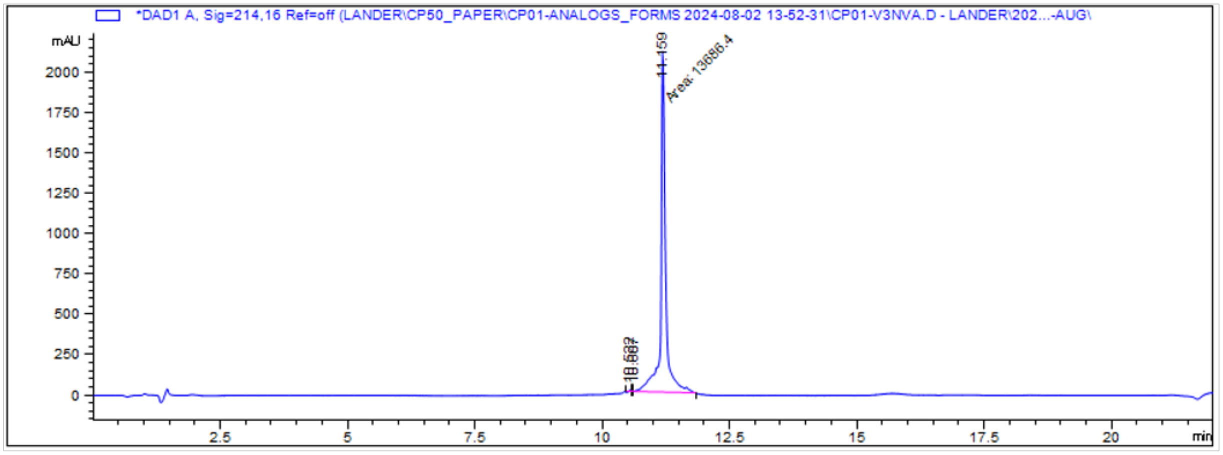

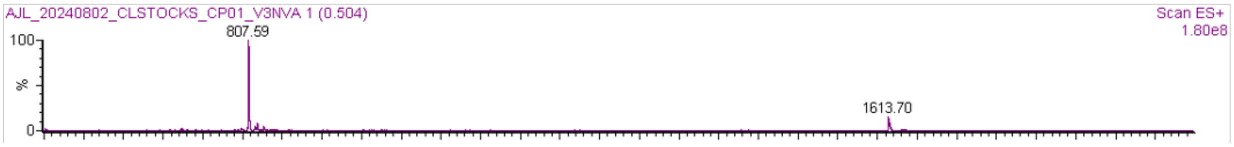

Figure S48. Chemical structure, calculated masses, HPLC chromatogram (214 nm) and ESI+ mass spectrum of isolated **Cp01 V3Nva**. Analytical HPLC purity: >98%, ESI+ MS (m/z): calculated 807.4 $[M+2H]^{2+}$, 1613.7 $[M+H]^{+}$, observed 807.6 $[M+2H]^{2+}$, 1613.7 $[M+H]^{+}$.

Cp01 Q5K

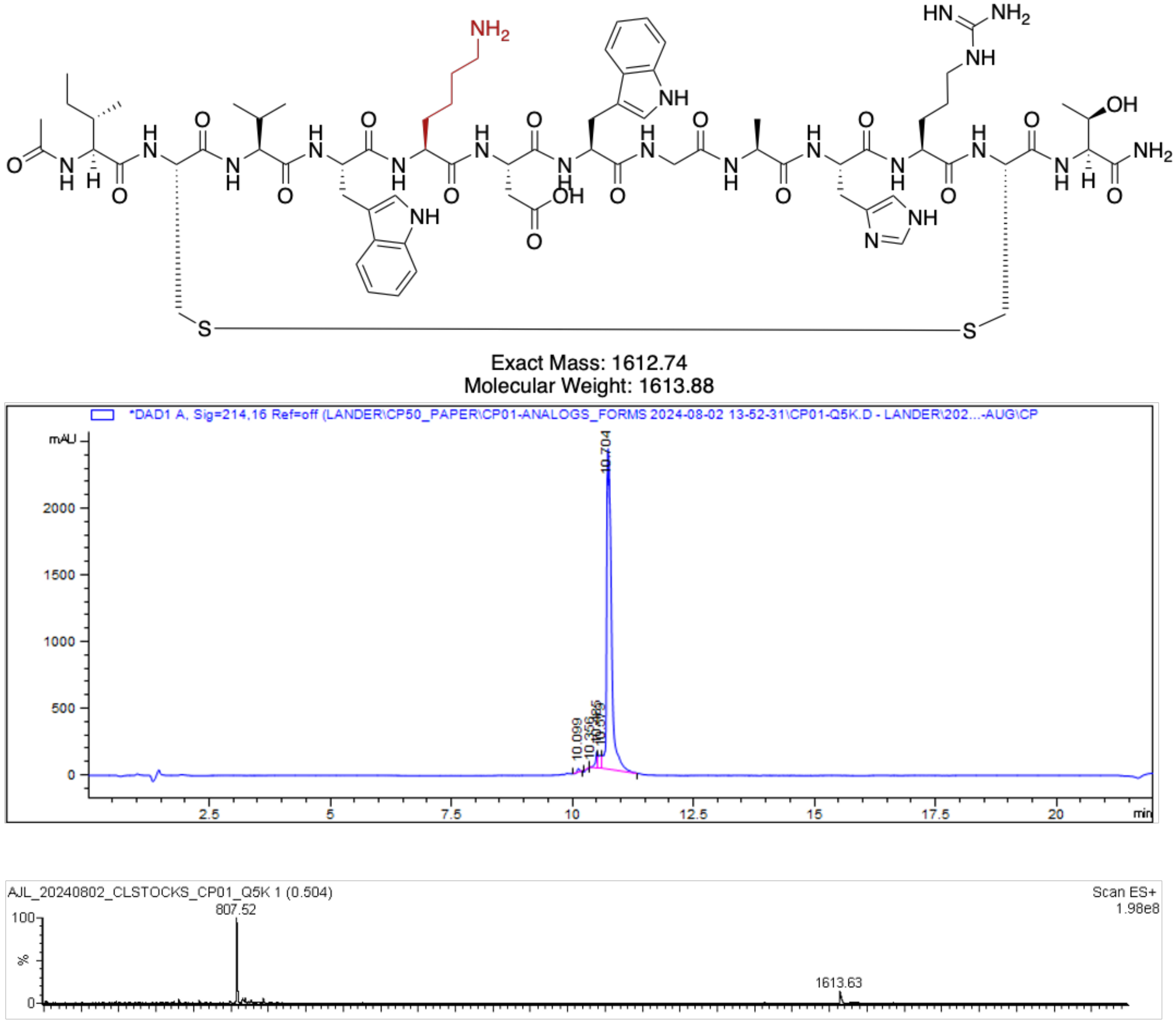

Figure S49. Chemical structure, calculated masses, HPLC chromatogram (214 nm) and ESI+ mass spectrum of isolated **Cp01 Q5K**. Analytical HPLC purity: 94%, ESI+ MS (m/z): calculated 807.4 $[M+2H]^{2+}$, 1613.7 $[M+H]^{+}$, observed 807.5 $[M+2H]^{2+}$, 1613.6 $[M+H]^{+}$.

Cp01 Q5Orn

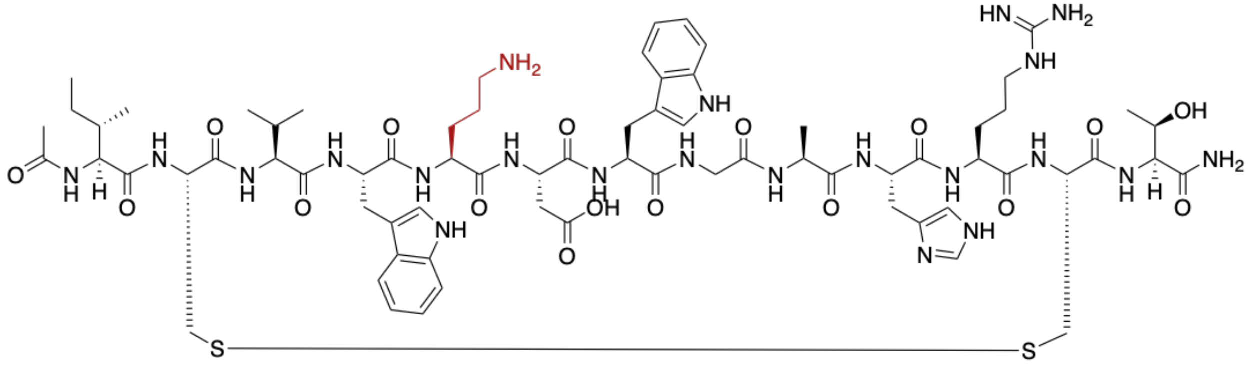

Exact Mass: 1598.72
Molecular Weight: 1599.85

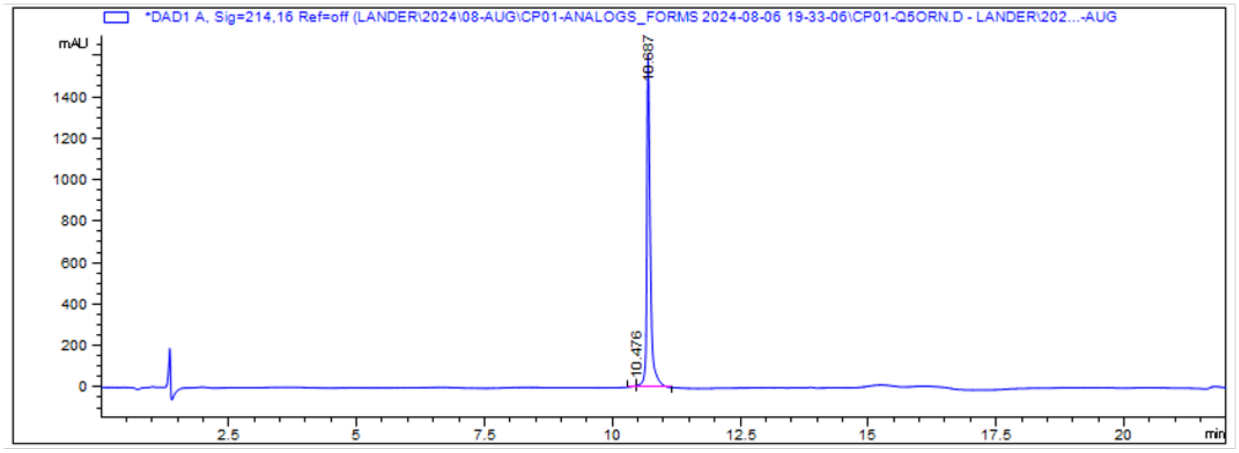

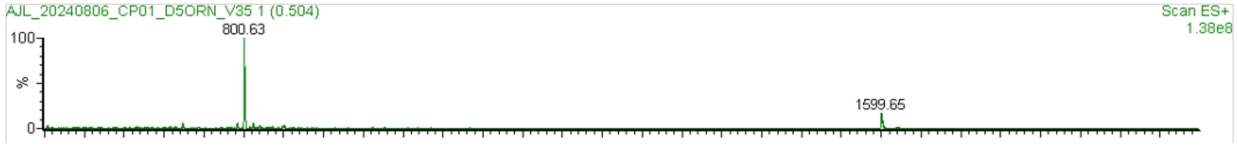

Figure S50. Chemical structure, calculated masses, HPLC chromatogram (214 nm) and ESI+ mass spectrum of isolated **Cp01 Q5Orn**. Analytical HPLC purity: >98%, ESI+ MS (m/z): calculated 800.4 $[M+2H]^{2+}$, 1599.7 $[M+H]^{+}$, observed 800.6 $[M+2H]^{2+}$, 1599.7 $[M+H]^{+}$.

Cp01 D6S

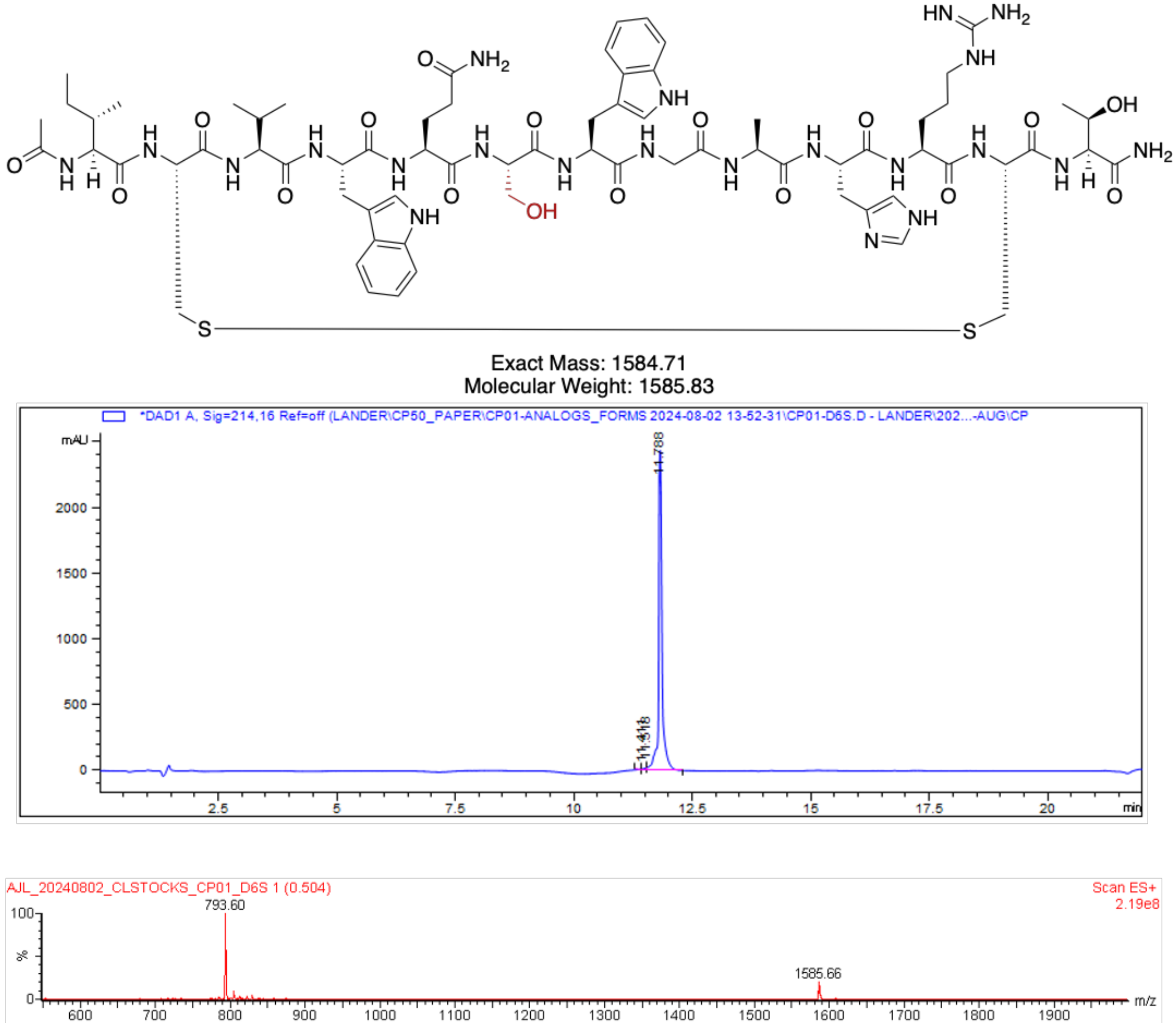

Figure S51. Chemical structure, calculated masses, HPLC chromatogram (214 nm) and ESI+ mass spectrum of isolated **Cp01 D6S**. Analytical HPLC purity: >98%, ESI+ MS (m/z): calculated 793.4 $[M+2H]^{2+}$, 1585.7 $[M+H]^{+}$, observed 793.6 $[M+2H]^{2+}$, 1585.7 $[M+H]^{+}$.

Cp01 D6N

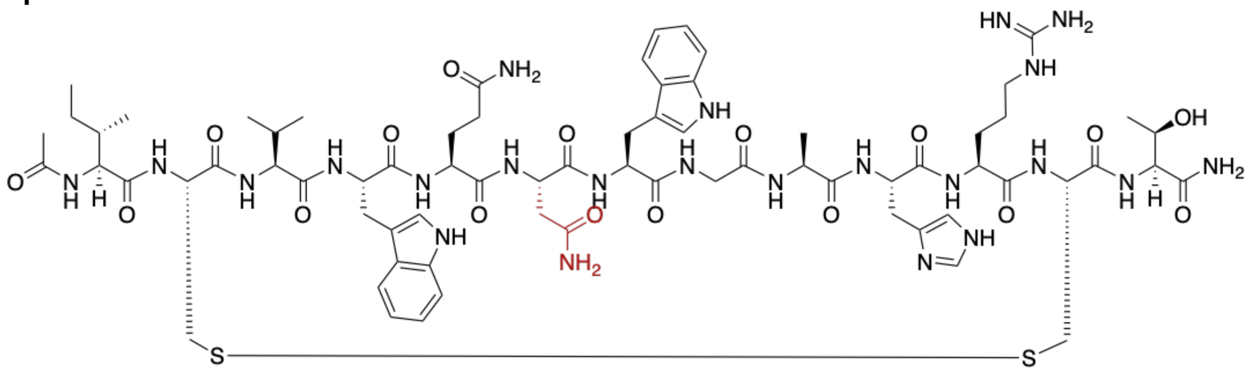

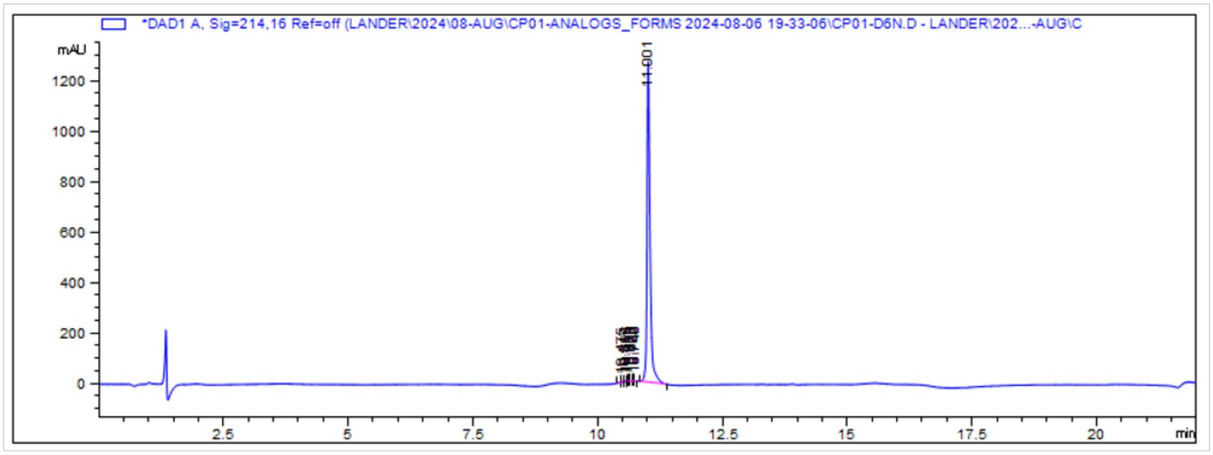

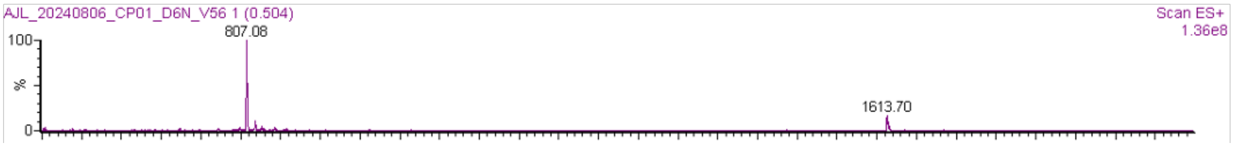

Figure S52. Chemical structure, calculated masses, HPLC chromatogram (214 nm) and ESI+ mass spectrum of isolated **Cp01 D6N**. Analytical HPLC purity: >98%, ESI+ MS (m/z): calculated 806.9 $[M+2H]^{2+}$, 1612.7 $[M+H]^{+}$, observed 807.1 $[M+2H]^{2+}$, 1613.7 $[M+H]^{+}$.

Cp01 D6E

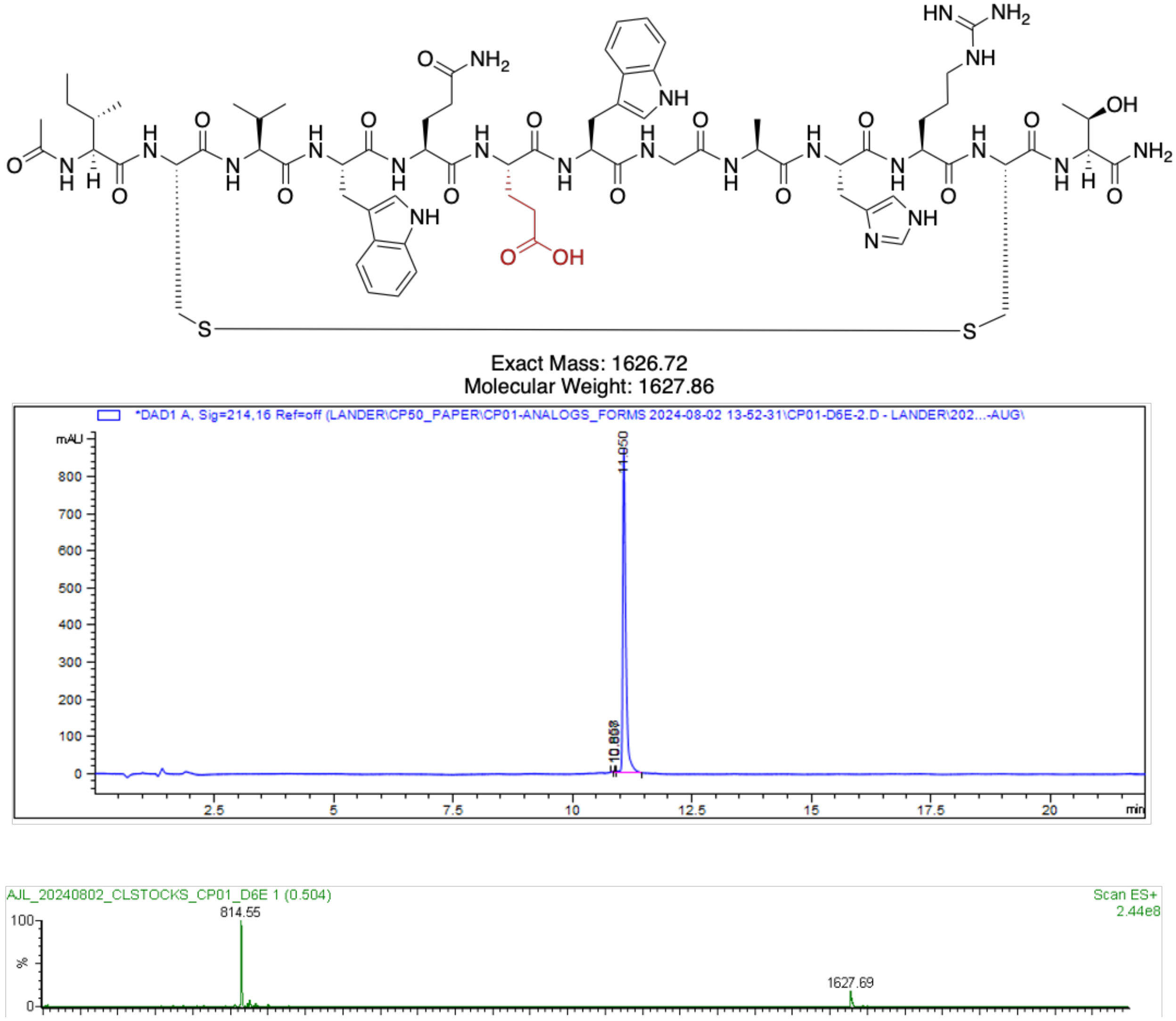

Figure S53. Chemical structure, calculated masses, HPLC chromatogram (214 nm) and ESI+ mass spectrum of isolated **Cp01 D6E**. Analytical HPLC purity: >98%, ESI+ MS (m/z): calculated 814.6 $[M+2H]^{2+}$, 1627.7 $[M+H]^{+}$, observed 814.6 $[M+2H]^{2+}$, 1627.7 $[M+H]^{+}$.

Cp01 D6Asu

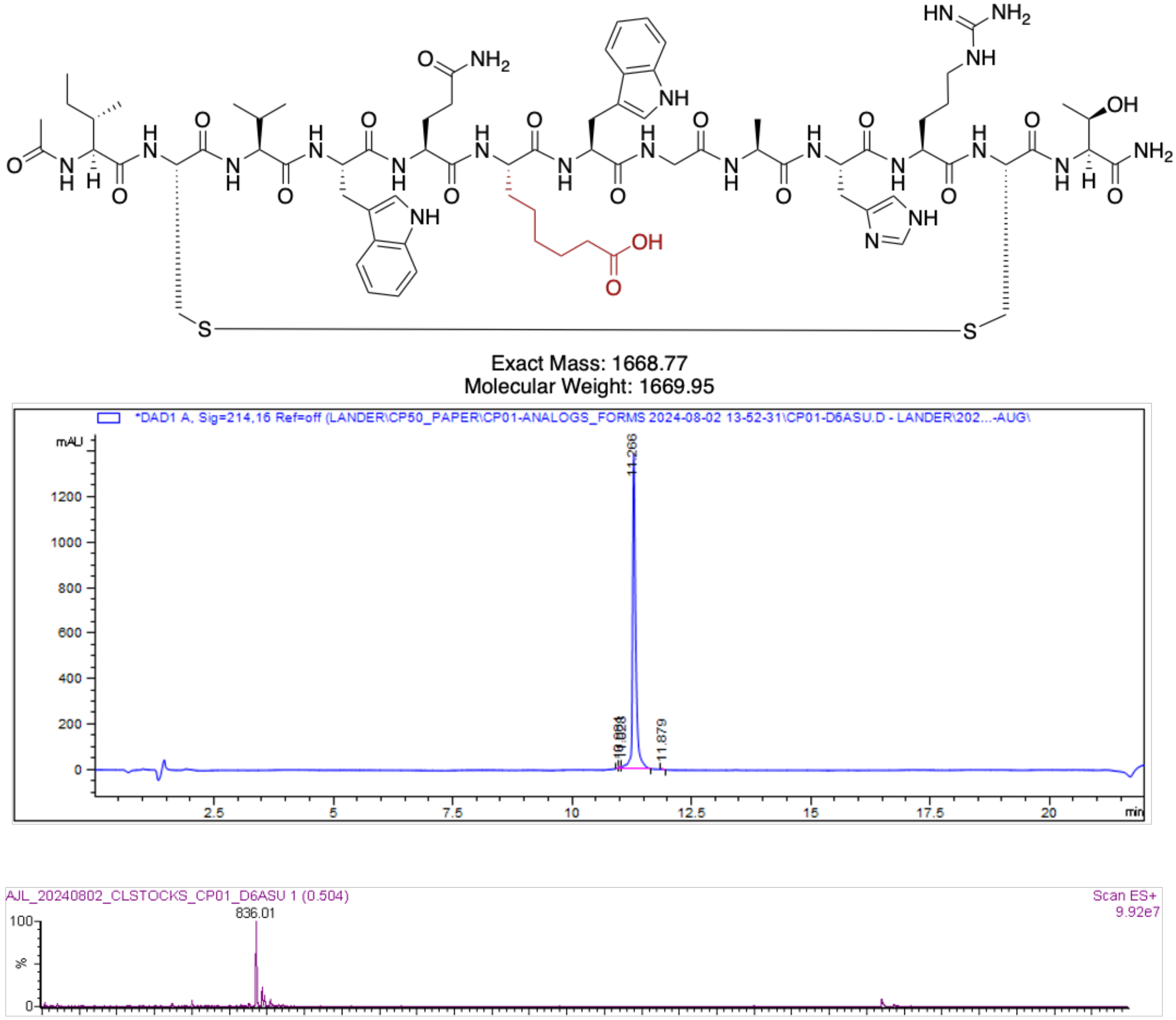

Figure S54. Chemical structure, calculated masses, HPLC chromatogram (214 nm) and ESI+ mass spectrum of isolated **Cp01 D6Asu**. Analytical HPLC purity: >98%, ESI+ MS (m/z): calculated 835.4 $[M+2H]^{2+}$, 1669.8 $[M+H]^{+}$, observed 835.4 $[M+2H]^{2+}$, 1669.8 $[M+H]^{+}$.

Cp01 W7Bta

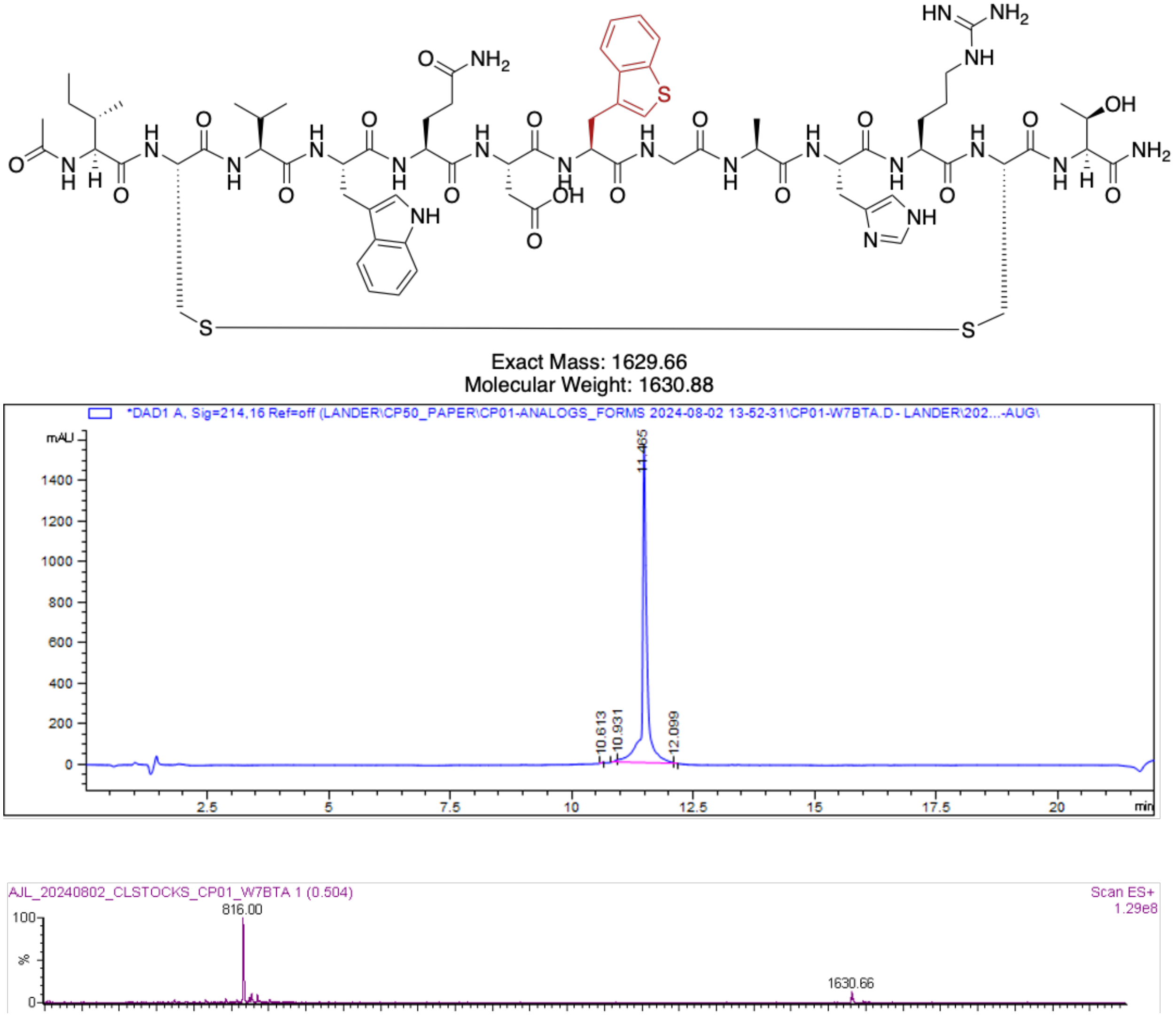

Figure S55. Chemical structure, calculated masses, HPLC chromatogram (214 nm) and ESI+ mass spectrum of isolated **Cp01 W7Bta**. Analytical HPLC purity: >90%, ESI+ MS (m/z): calculated 815.9 $[M+2H]^{2+}$, 1630.7 $[M+H]^{+}$, observed 816.0 $[M+2H]^{2+}$, 1630.7 $[M+H]^{+}$.

Cp01 G8A

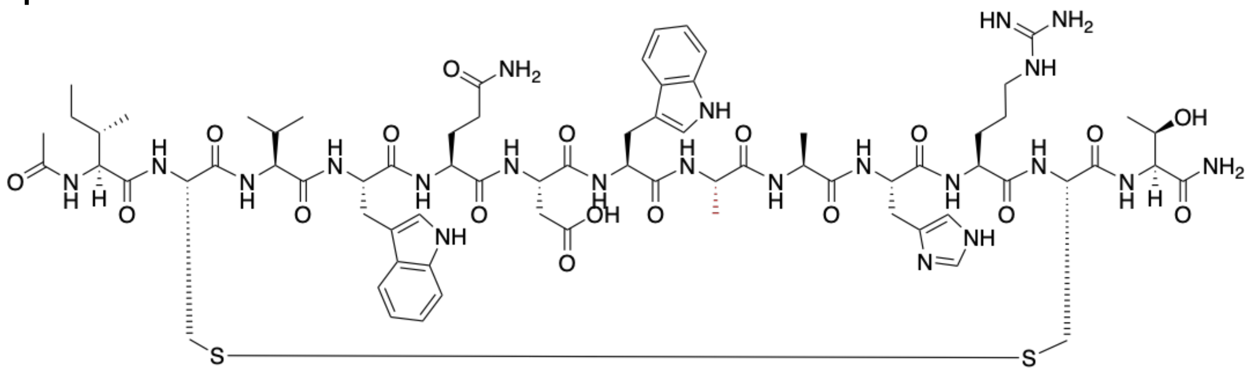

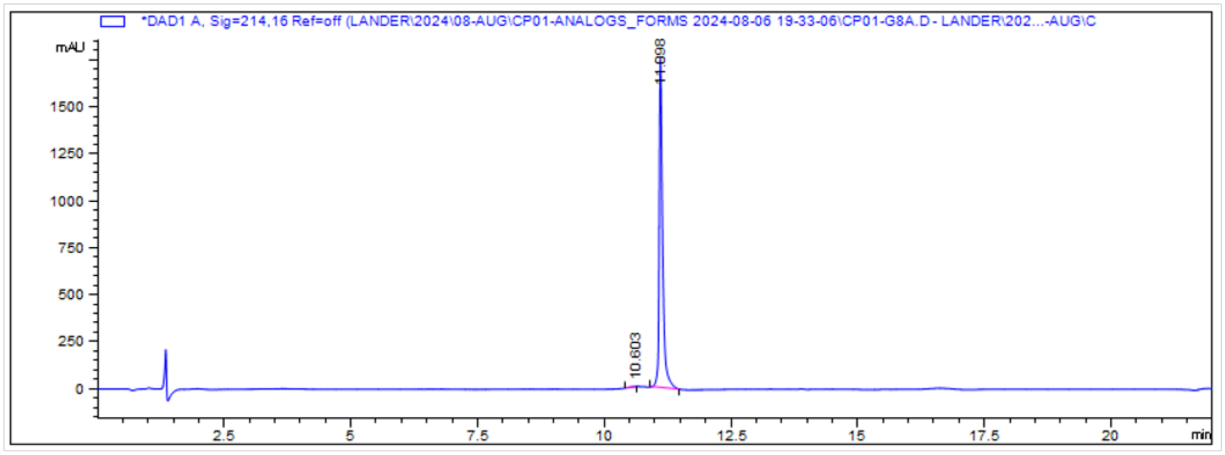

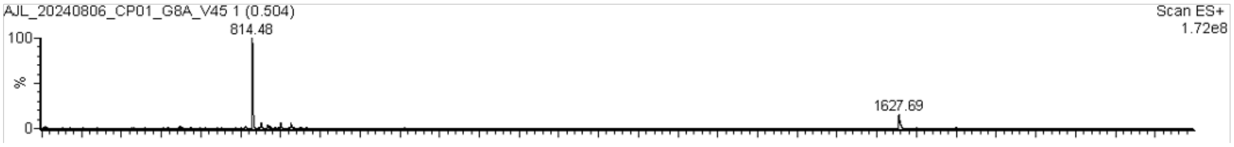

Figure S56. Chemical structure, calculated masses, HPLC chromatogram (214 nm) and ESI+ mass spectrum of isolated **Cp01 G8A**. Analytical HPLC purity: >98%, ESI+ MS (m/z): calculated 814.4 $[M+2H]^{2+}$, 1627.7 $[M+H]^{+}$, observed 814.5 $[M+2H]^{2+}$, 1627.7 $[M+H]^{+}$.

Cp01 G8dAla

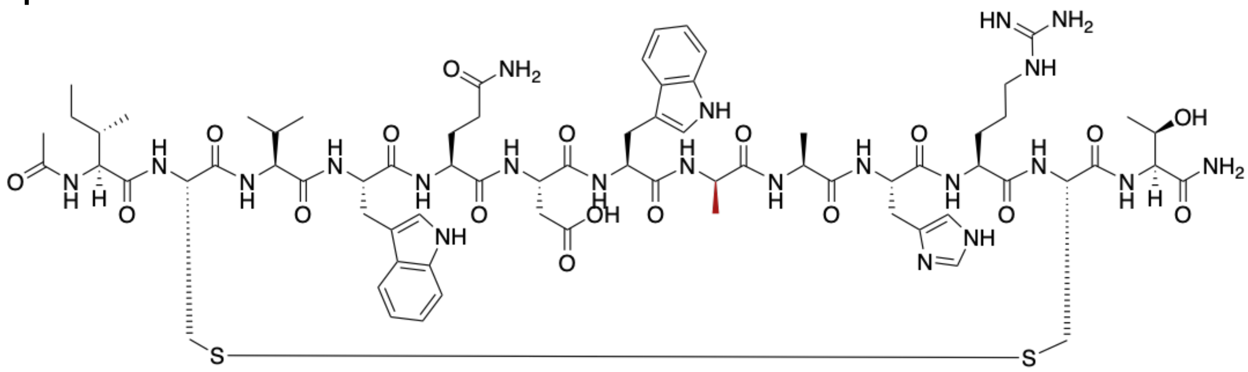

Exact Mass: 1626.72
Molecular Weight: 1627.86

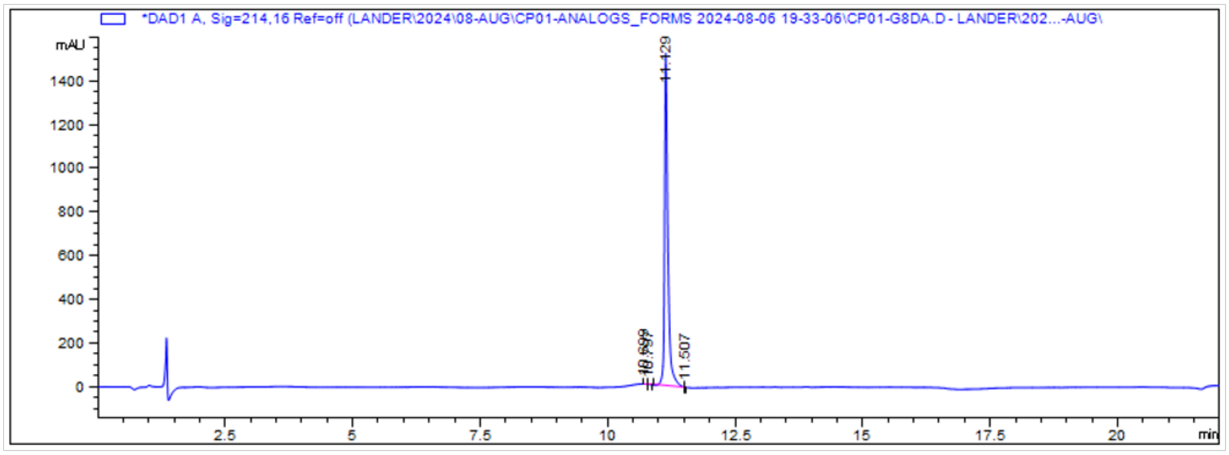

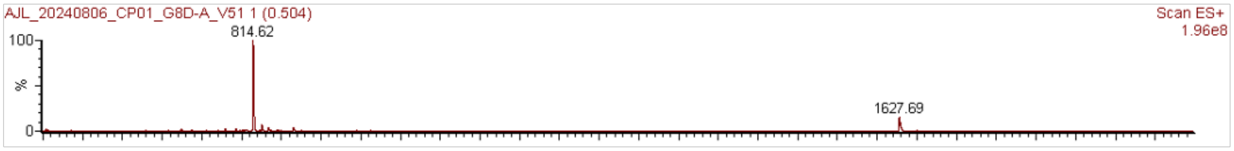

Figure S57. Chemical structure, calculated masses, HPLC chromatogram (214 nm) and ESI+ mass spectrum of isolated **Cp01 G8dAla**. Analytical HPLC purity: >98%, ESI+ MS (m/z): calculated 814.4 $[M+2H]^{2+}$, 1627.7 $[M+H]^{+}$, observed 814.6 $[M+2H]^{2+}$, 1627.7 $[M+H]^{+}$.

Cp01 R11K

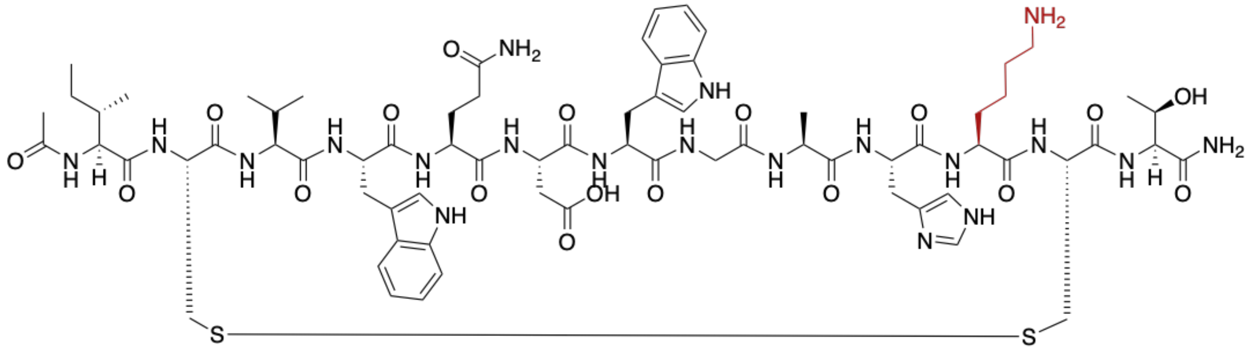

Exact Mass: 1584.70
Molecular Weight: 1585.82

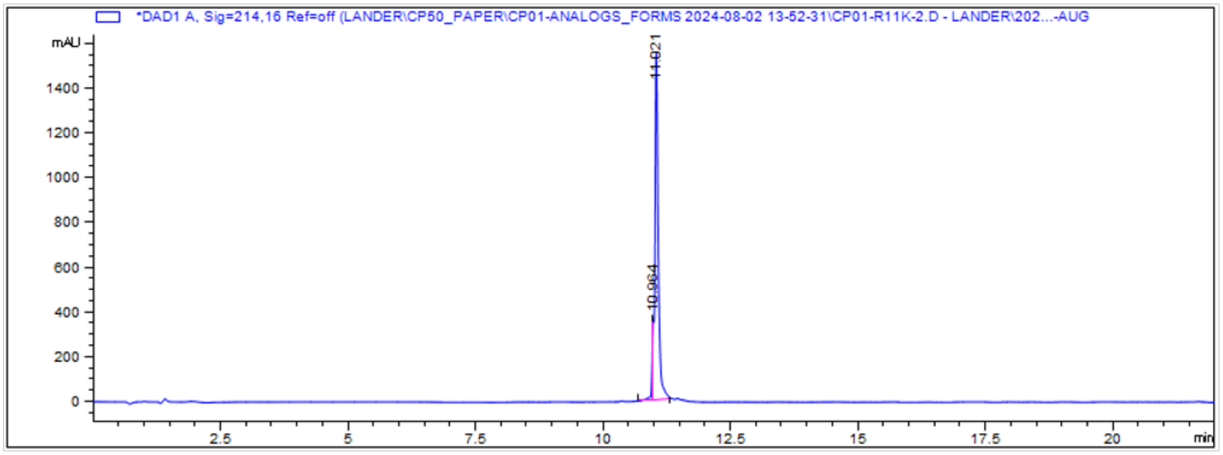

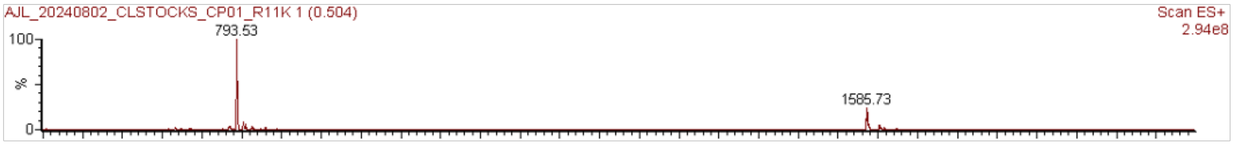

Figure S58. Chemical structure, calculated masses, HPLC chromatogram (214 nm) and ESI+ mass spectrum of isolated **Cp01 R11K**. Analytical HPLC purity: 90%, ESI+ MS (m/z): calculated 793.4 $[M+2H]^{2+}$, 1585.7 $[M+H]^{+}$, observed 793.5 $[M+2H]^{2+}$, 1585.7 $[M+H]^{+}$.

Cp01 R11S

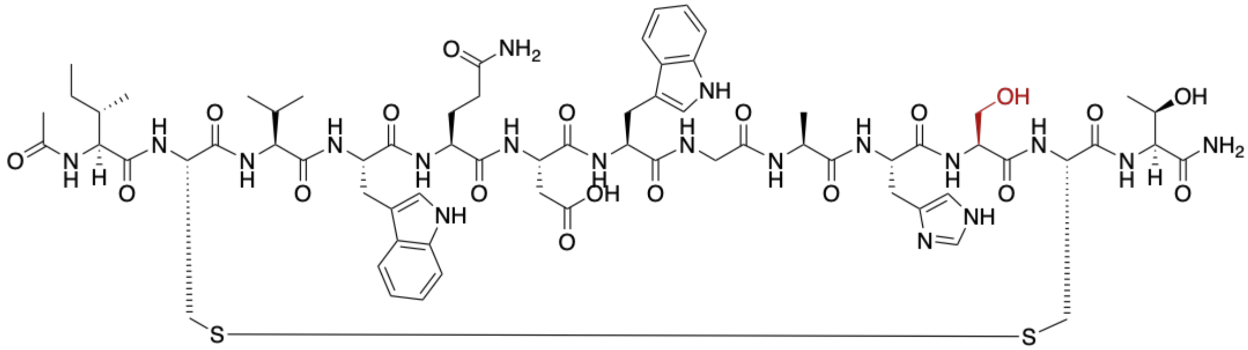

Exact Mass: 1543.63
Molecular Weight: 1544.73

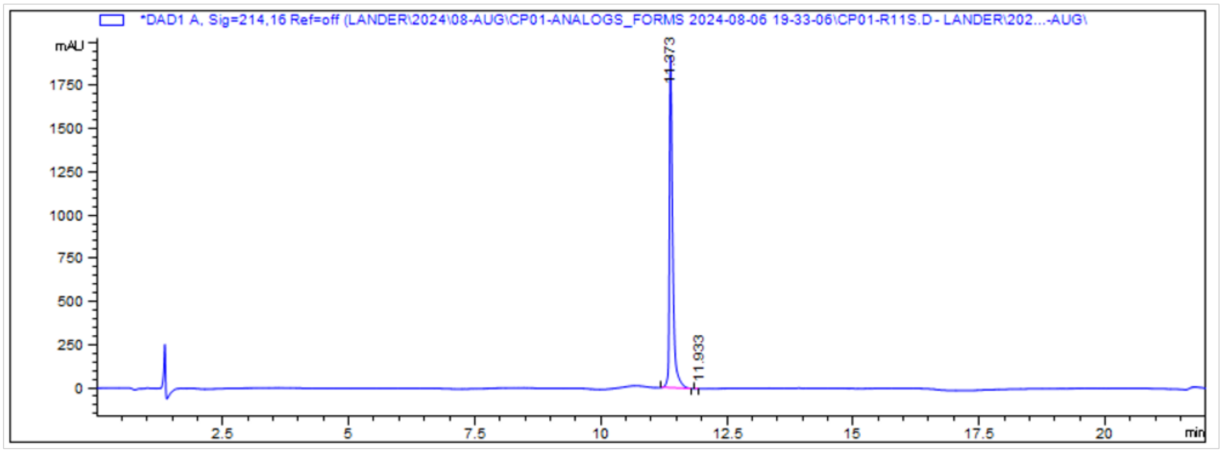

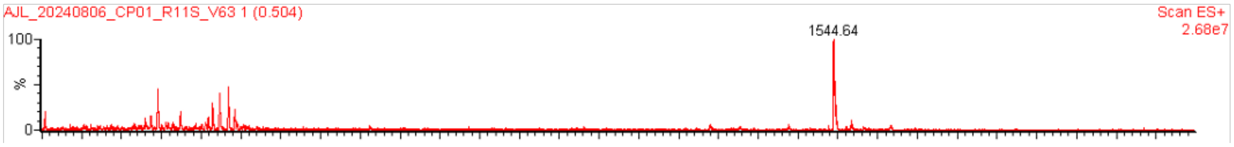

Figure S59. Chemical structure, calculated masses, HPLC chromatogram (214 nm) and ESI+ mass spectrum of isolated **Cp01 R11S**. Analytical HPLC purity: >98%, ESI+ MS (m/z): calculated 1544.6 $[M+H]^+$, observed 1544.6 $[M+H]^+$.

Cp05

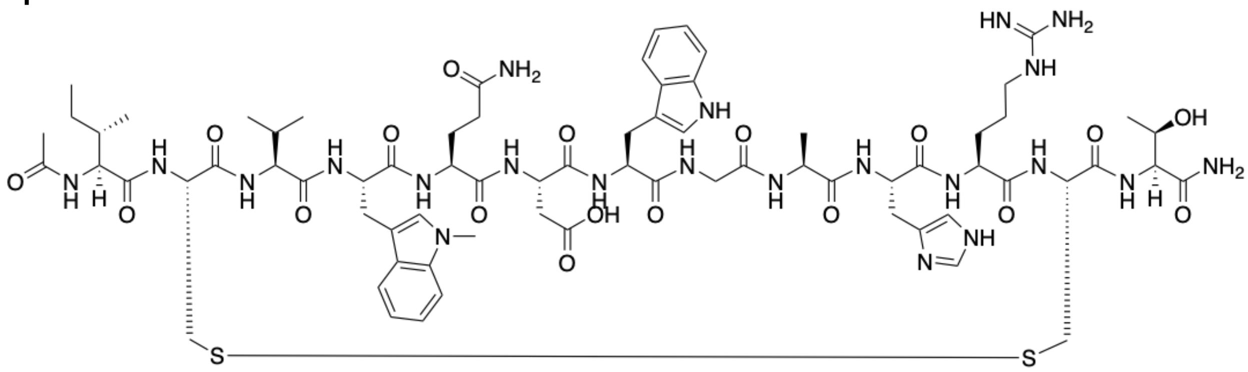

Exact Mass: 1626.72
Molecular Weight: 1627.86

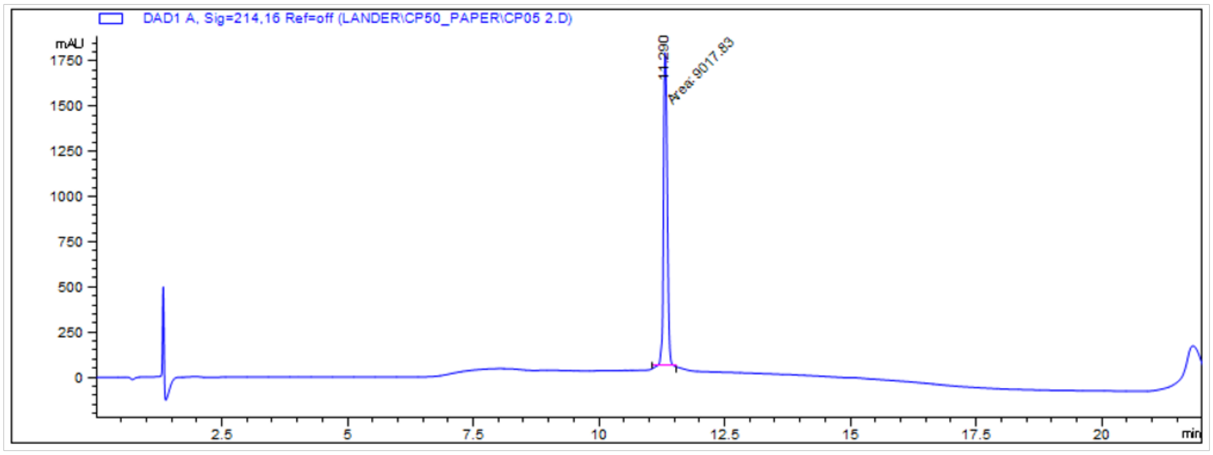

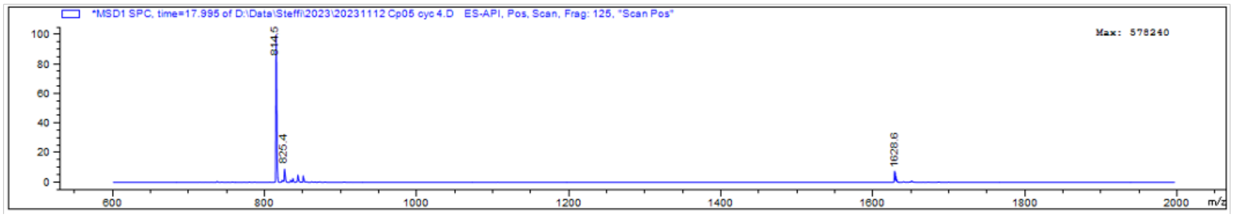

Figure S60. Chemical structure, calculated masses, HPLC chromatogram (214 nm) and ESI+ mass spectrum of isolated **Cp05**. Analytical HPLC purity: >98%, ESI+ MS (m/z): calculated 814.4 $[M+2H]^{2+}$, 1627.7 $[M+H]^{+}$, observed 814.5 $[M+2H]^{2+}$, 1628.6 $[M+H]^{+}$.

Cp05 V3I

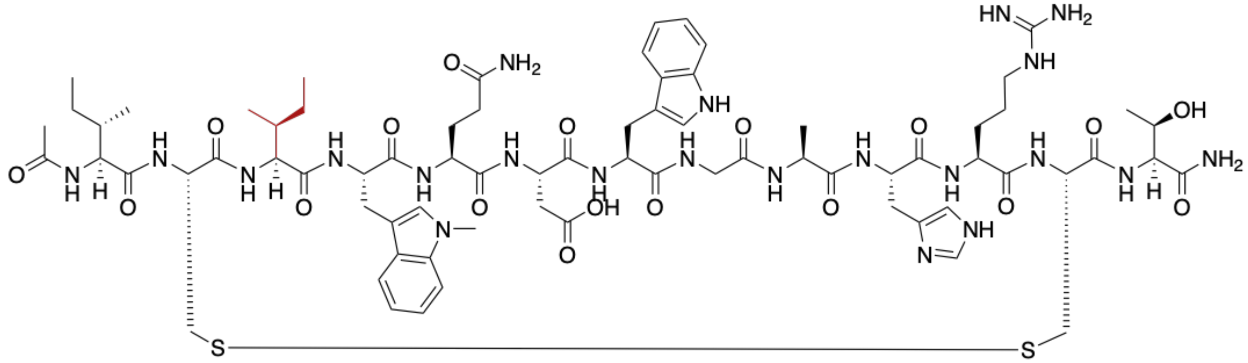

Exact Mass: 1640.73
Molecular Weight: 1641.89

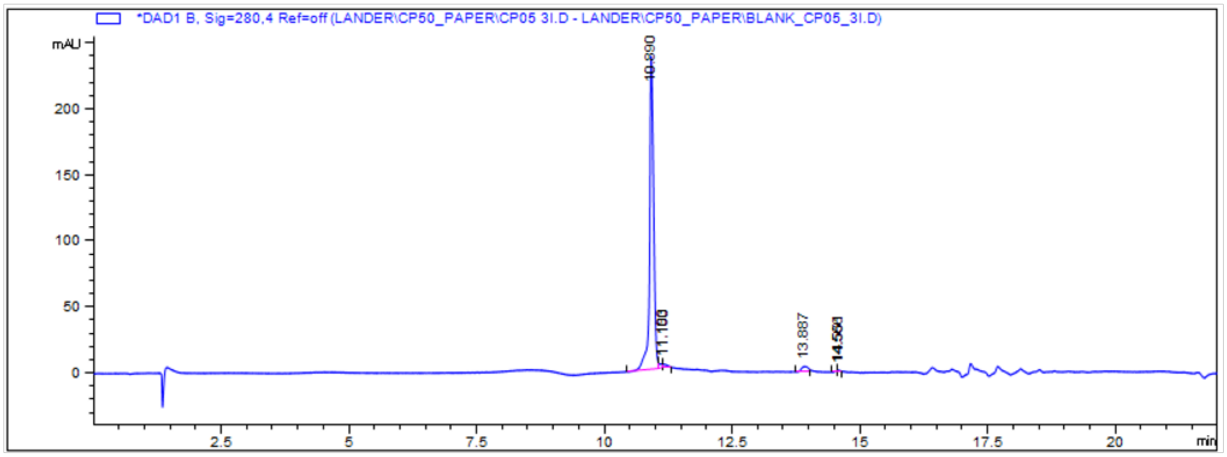

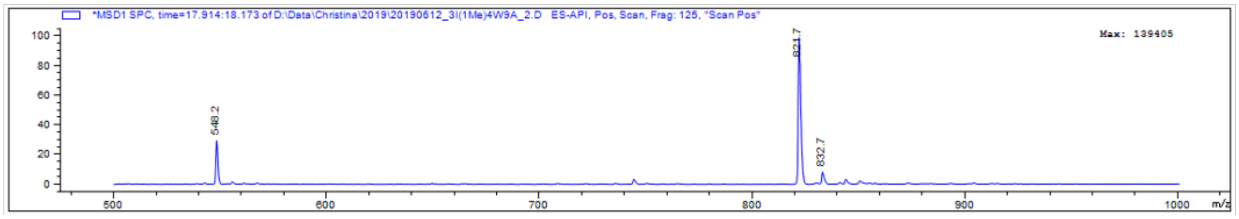

Figure S61. Chemical structure, calculated masses, HPLC chromatogram (214 nm) and ESI+ mass spectrum of isolated **Cp05 V3I**. Analytical HPLC purity: 95%, ESI+ MS (m/z): calculated 547.9 $[M+3H]^{3+}$, 821.4 $[M+2H]^{2+}$, observed 546.2 $[M+3H]^{3+}$, 821.7 $[M+2H]^{2+}$.

Cp40

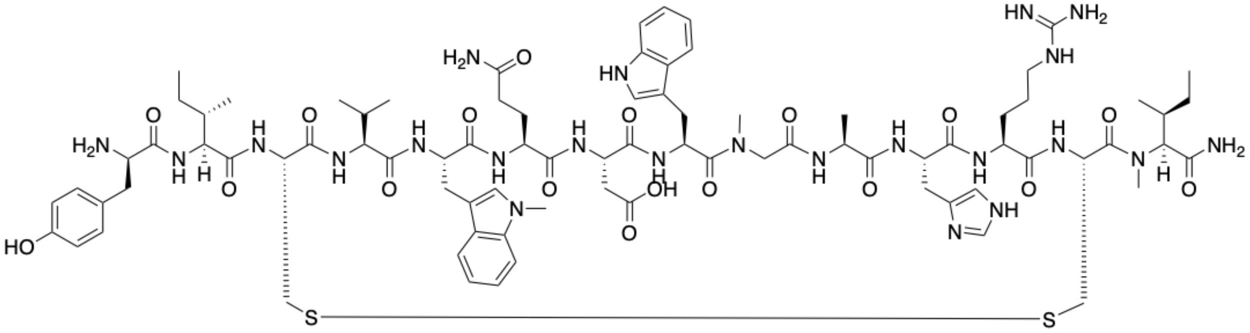

Exact Mass: 1787.84
Molecular Weight: 1789.11

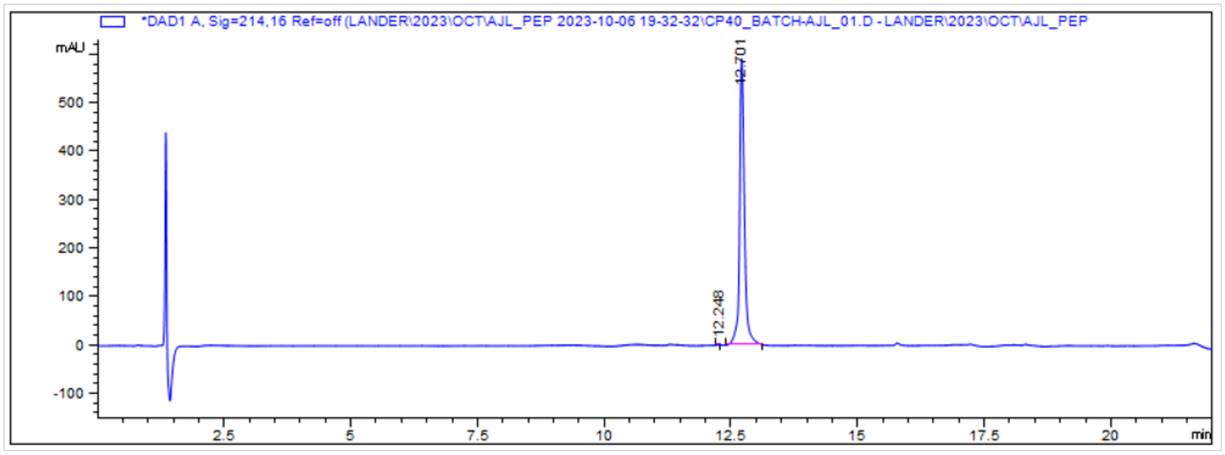

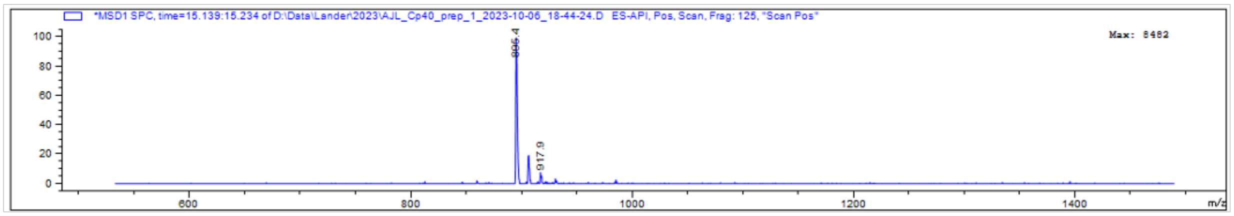

Figure S62. Chemical structure, calculated masses, HPLC chromatogram (214 nm) and ESI+ mass spectrum of isolated **Cp40**. Analytical HPLC purity: >98%, ESI+ MS (m/z): calculated 894.9 $[M+2H]^{2+}$, observed 894.4 $[M+2H]^{2+}$.

Cp60

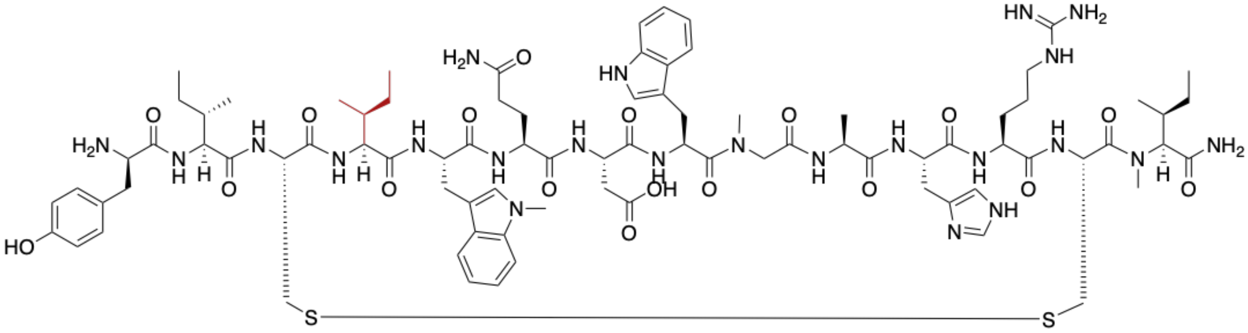

Exact Mass: 1801.85
Molecular Weight: 1803.14

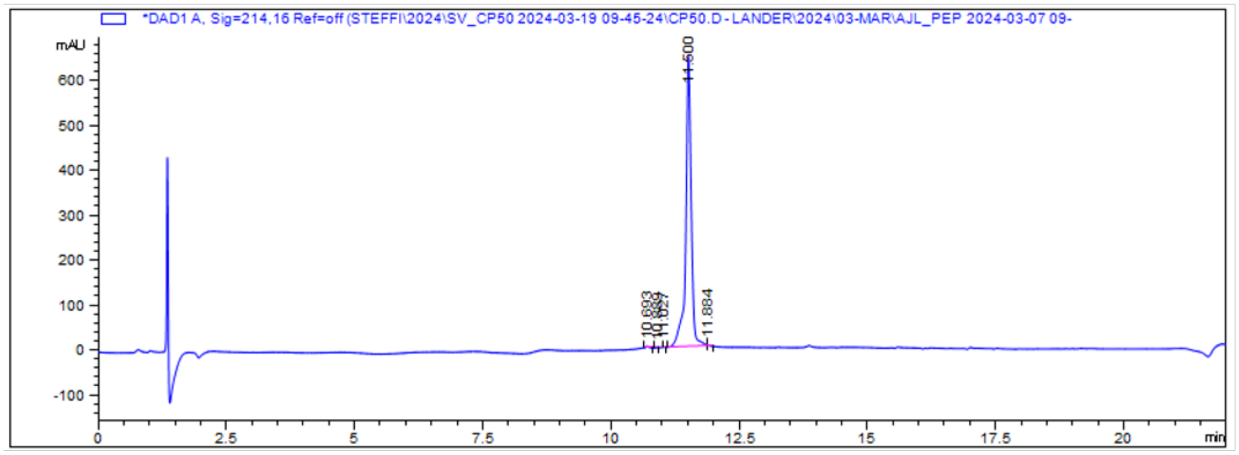

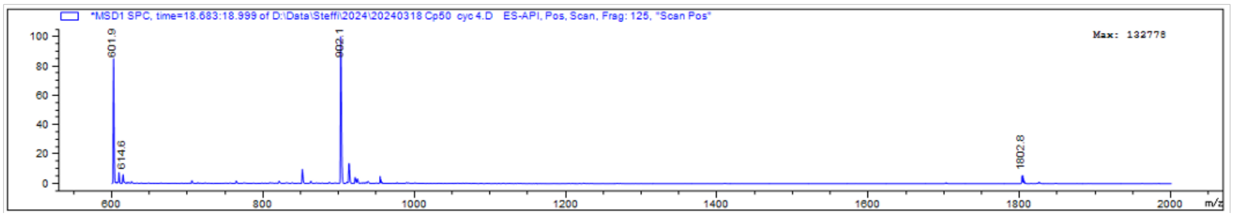

Figure S63. Chemical structure, calculated masses, HPLC chromatogram (214 nm) and ESI+ mass spectrum of isolated **Cp60**. Analytical HPLC purity: >98%, ESI+ MS (m/z): calculated 601.6 $[M+3H]^{3+}$, 902.0 $[M+2H]^{2+}$, 1802.9 $[M+H]^{+}$, observed 601.9 $[M+3H]^{3+}$, 902.1 $[M+2H]^{2+}$, 1802.8 $[M+H]^{+}$.

Cp01 V3aIle

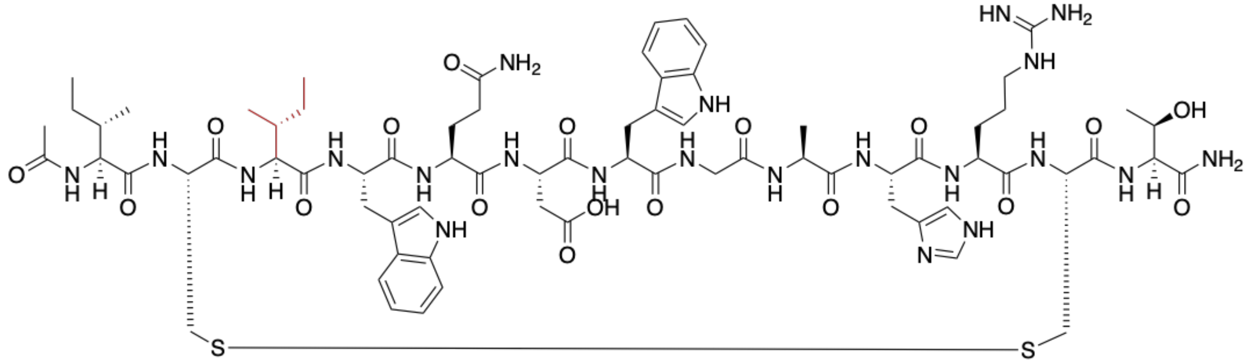

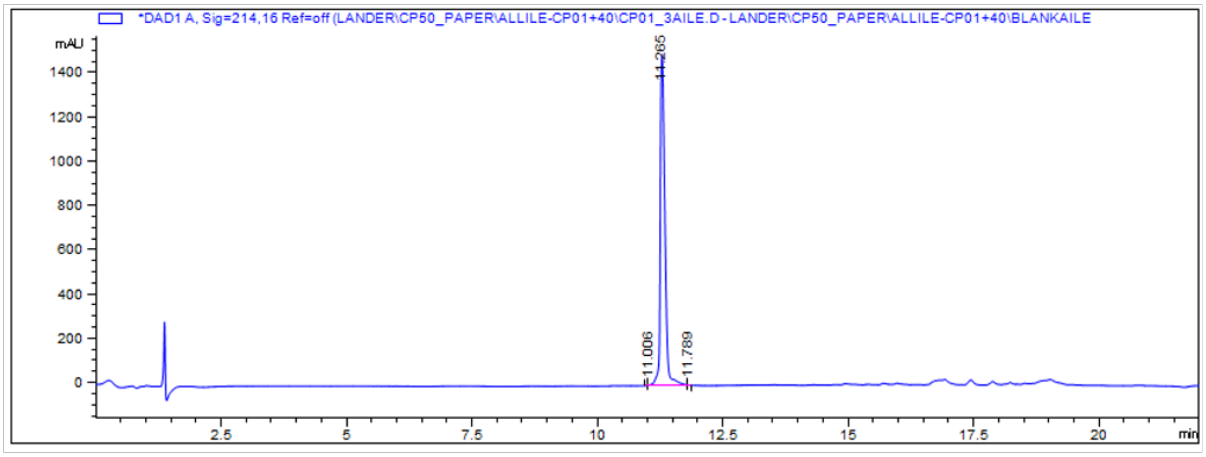

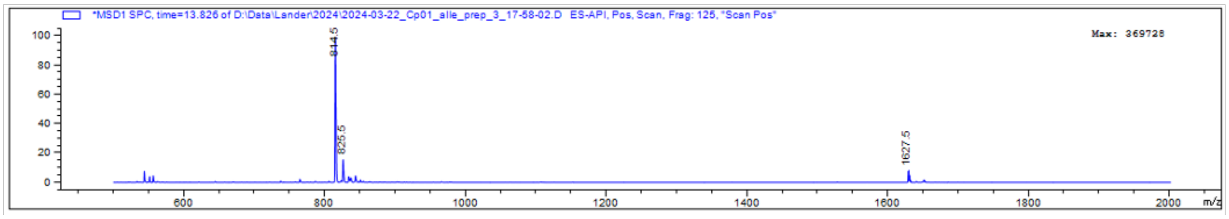

Figure S64. Chemical structure, calculated masses, HPLC chromatogram (214 nm) and ESI+ mass spectrum of isolated **Cp01 V3aIle**. Analytical HPLC purity: >98%, ESI+ MS (m/z): calculated 814.5 $[M+2H]^{2+}$, 1627.5 $[M+H]^{+}$, observed 814.5 $[M+2H]^{2+}$, 1627.5 $[M+H]^{+}$.

Cp01 V3Dea

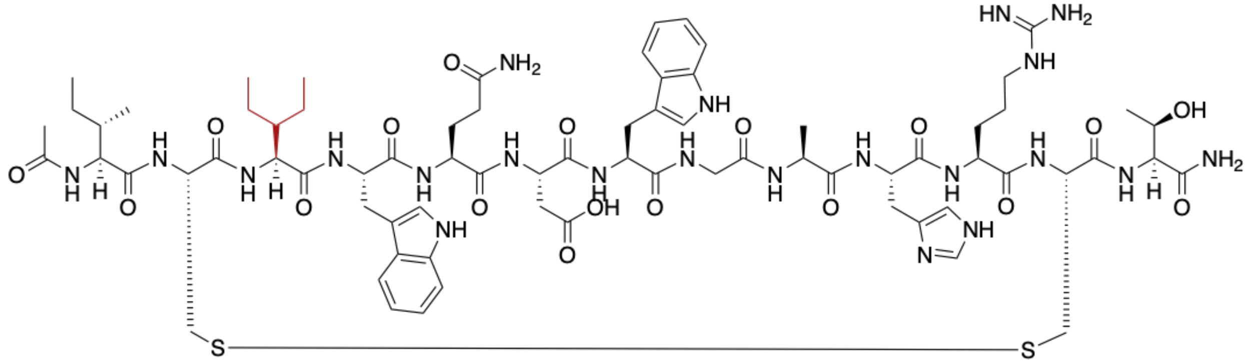

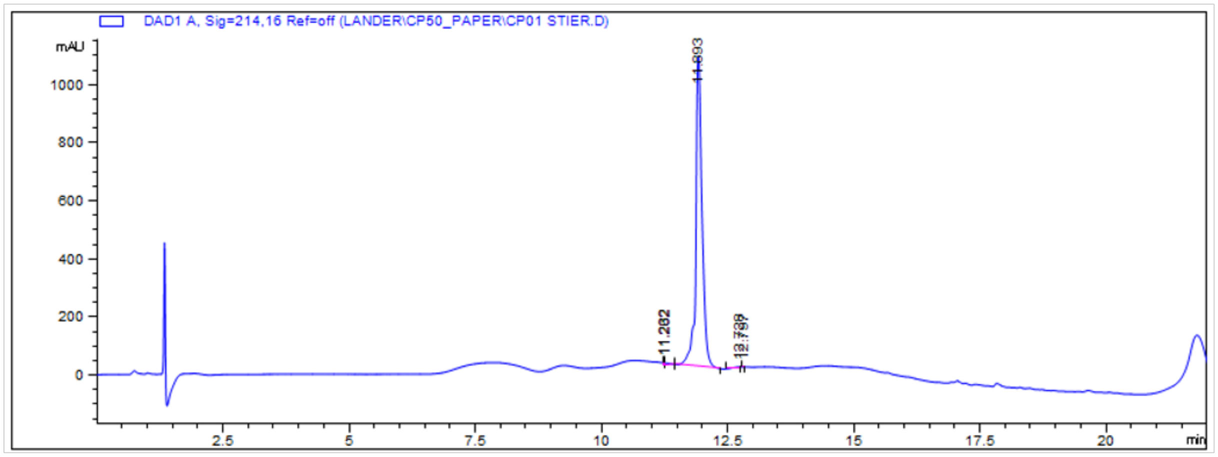

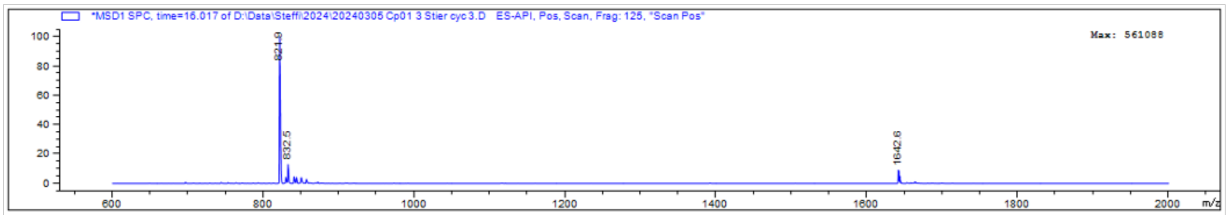

Figure S65. Chemical structure, calculated masses, HPLC chromatogram (214 nm) and ESI+ mass spectrum of isolated **Cp40 V3Dea**. Analytical HPLC purity: >98%, ESI+ MS (m/z): calculated 821.4 $[M+2H]^{2+}$, 1641.7 $[M+H]^{+}$, observed 821.9 $[M+2H]^{2+}$, 1642.6 $[M+H]^{+}$.

Cp40 V3aIle

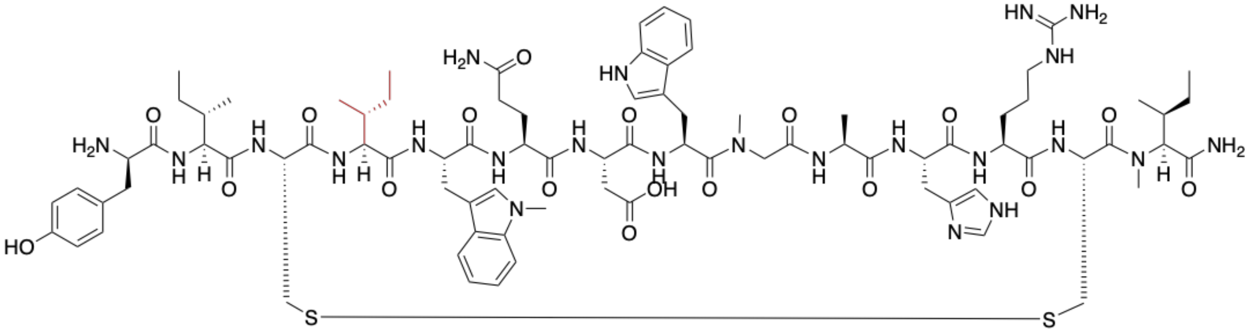

Exact Mass: 1801.85
Molecular Weight: 1803.14

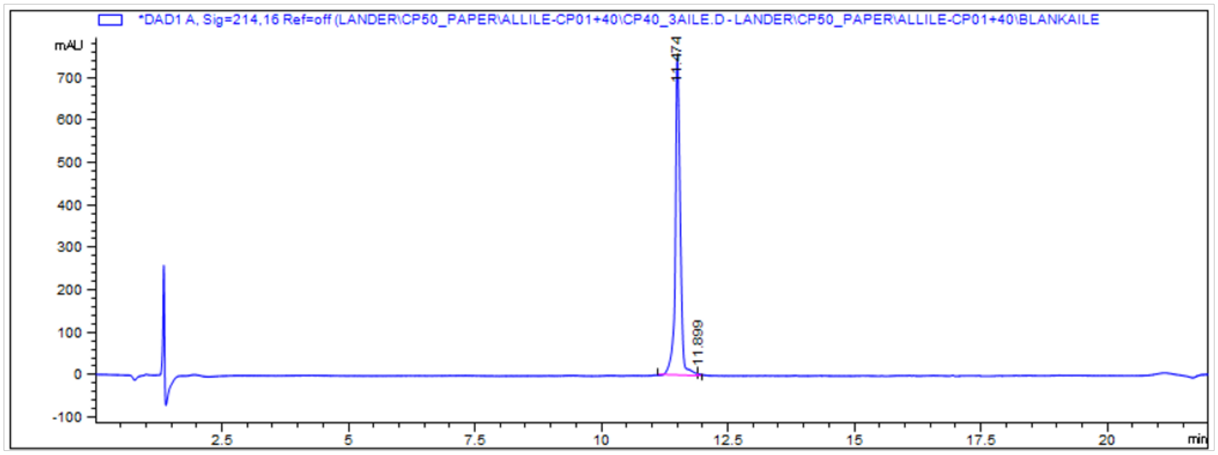

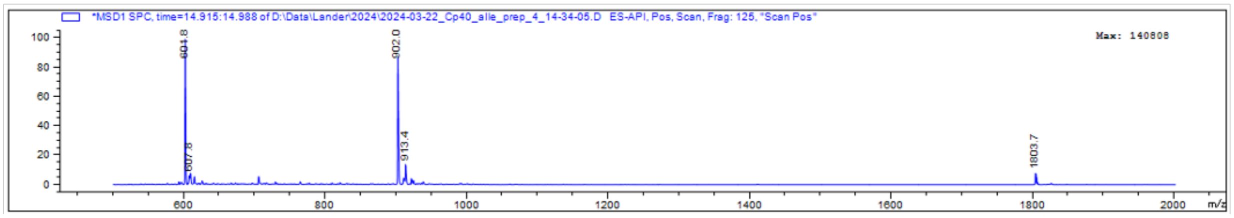

Figure S66. Chemical structure, calculated masses, HPLC chromatogram (214 nm) and ESI+ mass spectrum of isolated **Cp40 V3aile**. Analytical HPLC purity: >98%, ESI+ MS (m/z): calculated 601.6 $[M+3H]^{3+}$, 902.0 $[M+2H]^{2+}$, 1802.9 $[M+H]^{+}$, observed 601.8 $[M+3H]^{3+}$, 902.0 $[M+2H]^{2+}$, 1803.7 $[M+H]^{+}$.

Cp40 V3Dea

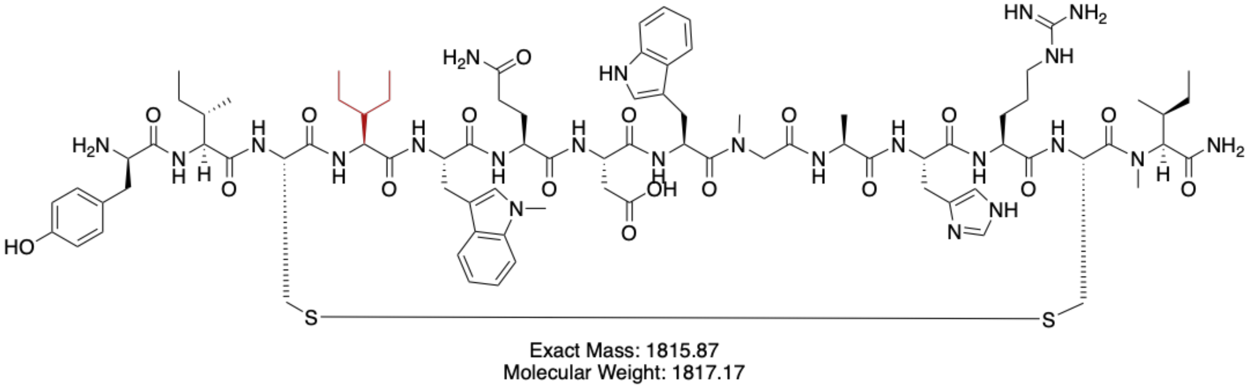

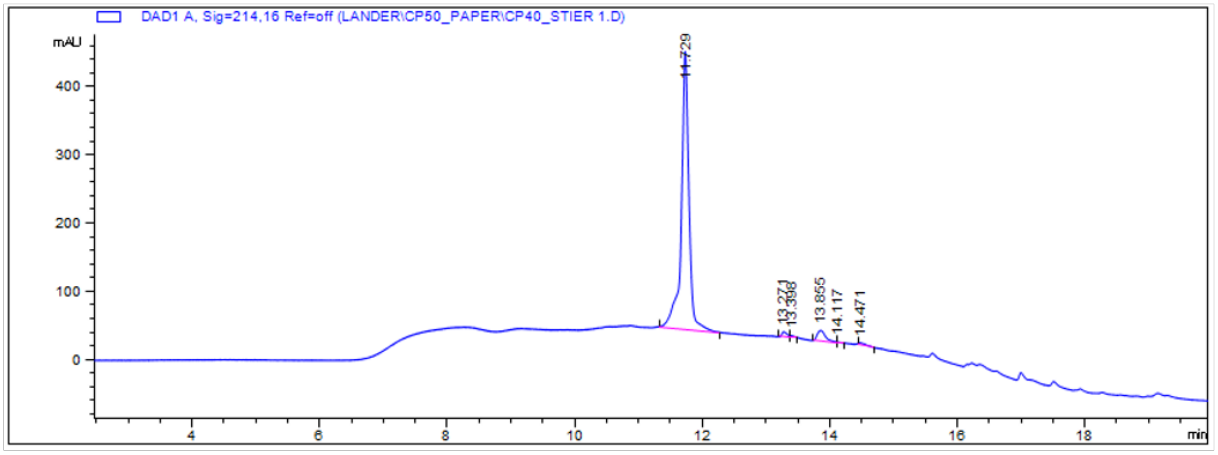

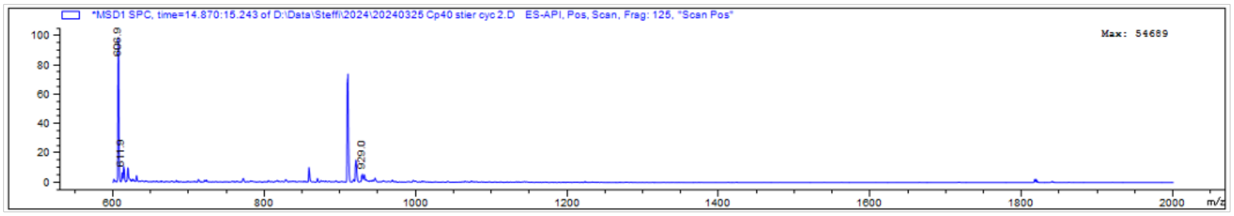

Figure S67. Chemical structure, calculated masses, HPLC chromatogram (214 nm) and ESI+ mass spectrum of isolated **Cp40 V3Dea**. Analytical HPLC purity: 95%, ESI+ MS (m/z): calculated 606.3 $[M+3H]^{3+}$, 909.0 $[M+2H]^{2+}$, observed 606.9 $[M+3H]^{3+}$, 909.1 $[M+2H]^{2+}$.

Cp01 K14(sulfo-Cyanine5)

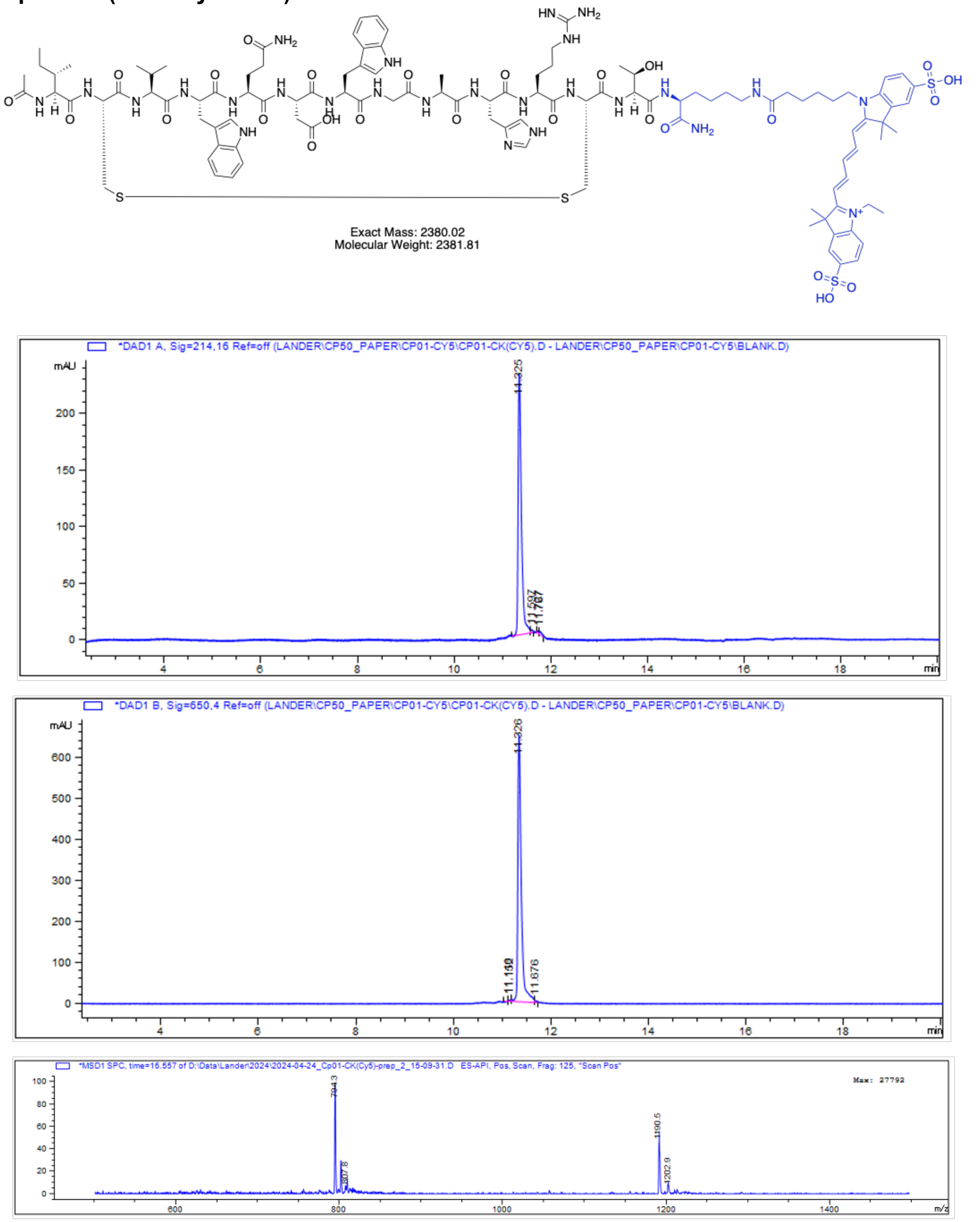

Figure S68. Chemical structure, calculated masses, HPLC chromatograms (214 and 650 nm) and ESI+ mass spectrum of isolated **Cp01 K14(sulfo-Cyanine5)**. Analytical HPLC purity: >98%, ESI+ MS (m/z): calculated 794.3 $[M+3H]^{3+}$, 1191.0 $[M+2H]^{2+}$, observed 794.3 $[M+3H]^{3+}$, 1190.5 $[M+2H]^{2+}$.

Cp01 V3I K14(sulfo-Cyanine5)

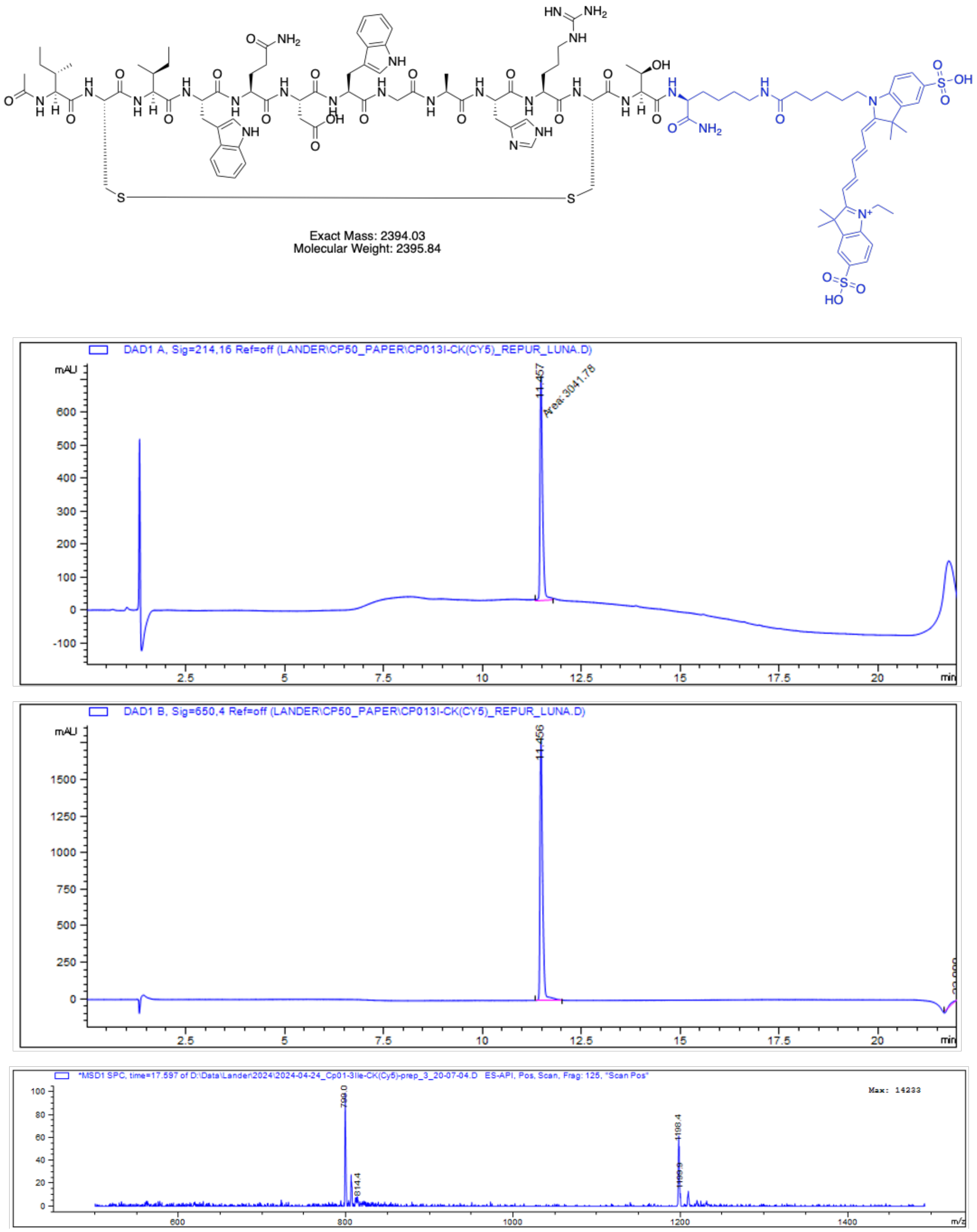

Figure S69. Chemical structure, calculated masses, HPLC chromatograms (214 and 650 nm) and ESI+ mass spectrum of isolated **Cp01 V3I K14(sulfo-Cyanine5)**. Analytical HPLC purity: >98%, ESI+ MS (m/z): calculated 799.0 $[M+3H]^{3+}$, 1198.0 $[M+2H]^{2+}$, observed 799.0 $[M+3H]^{3+}$, 1198.4 $[M+2H]^{2+}$.

Cp40 K14(sulfo-Cyanine5)

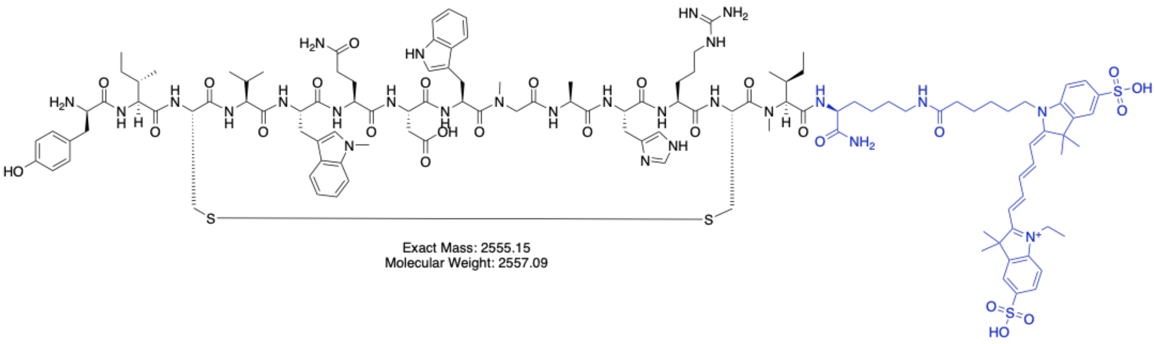

Exact Mass: 2555.15
Molecular Weight: 2557.09

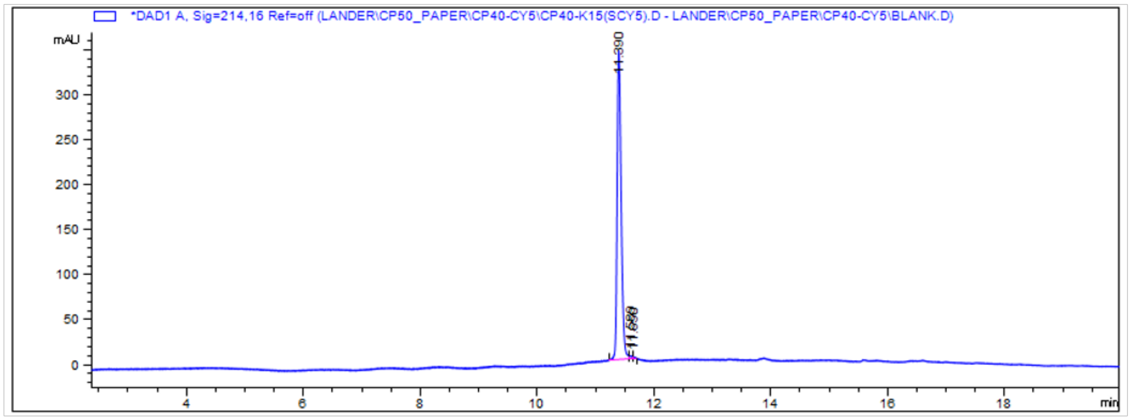

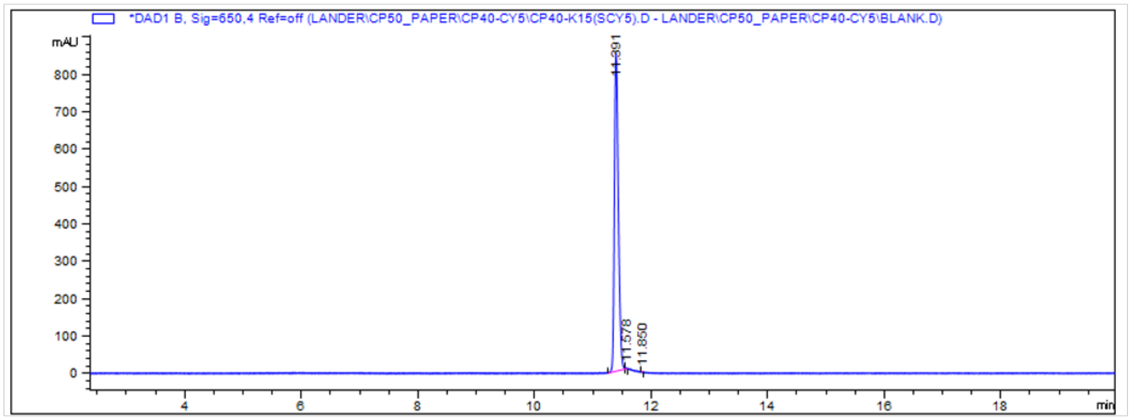

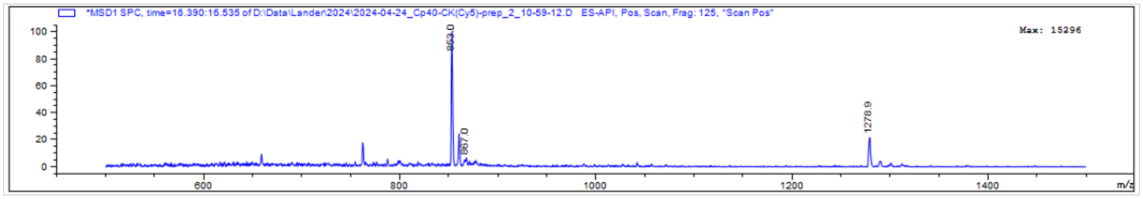

Figure S70. Chemical structure, calculated masses, HPLC chromatograms (214 and 650 nm) and ESI+ mass spectrum of isolated **Cp40 K14(sulfo-Cyanine5)**. Analytical HPLC purity: >98%, ESI+ MS (m/z): calculated 852.7 $[M+3H]^{3+}$, 1278.6 $[M+2H]^{2+}$, observed 853.0 $[M+3H]^{3+}$, 1278.9 $[M+2H]^{2+}$.

Cp60 K14(sulfo-Cyanine5)

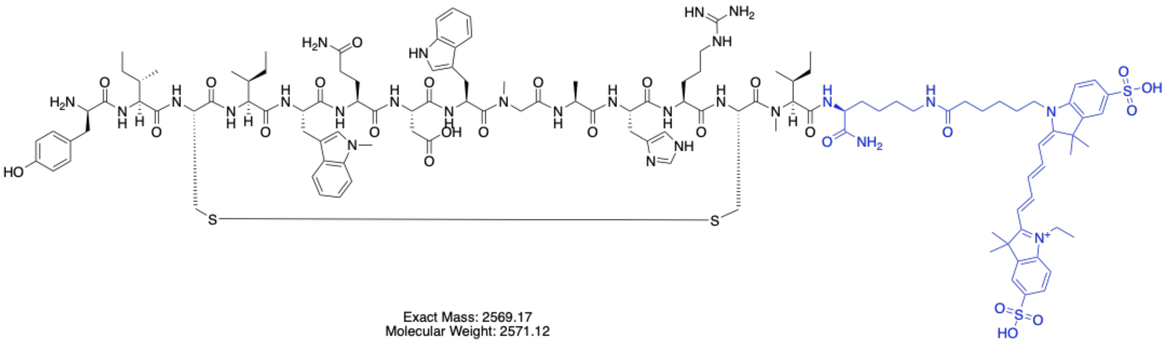

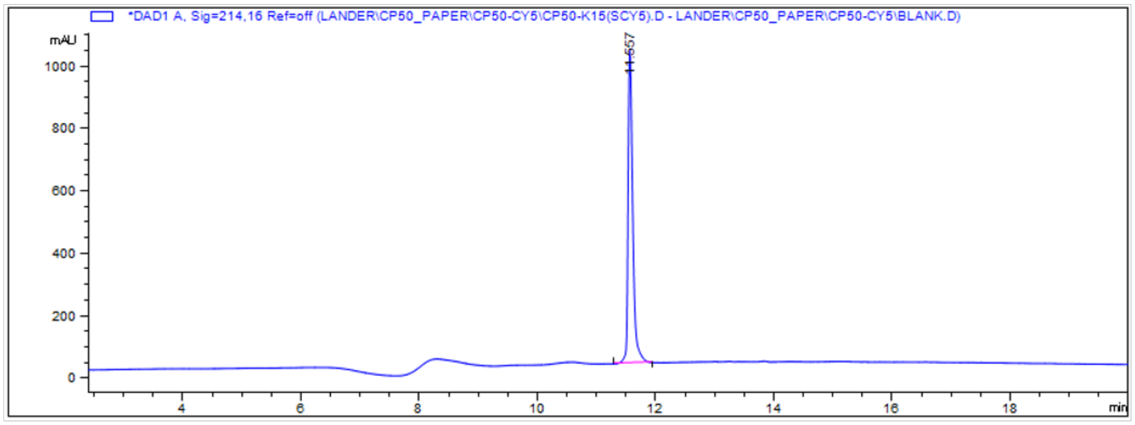

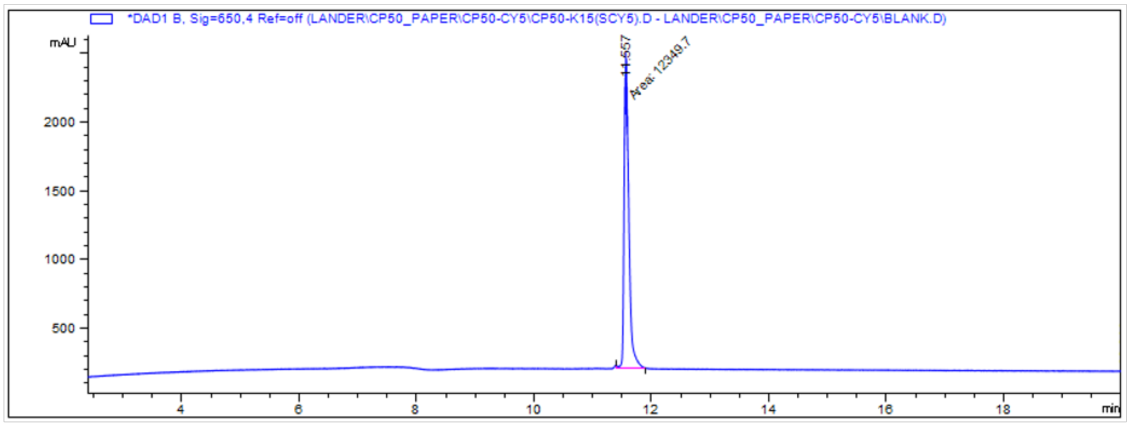

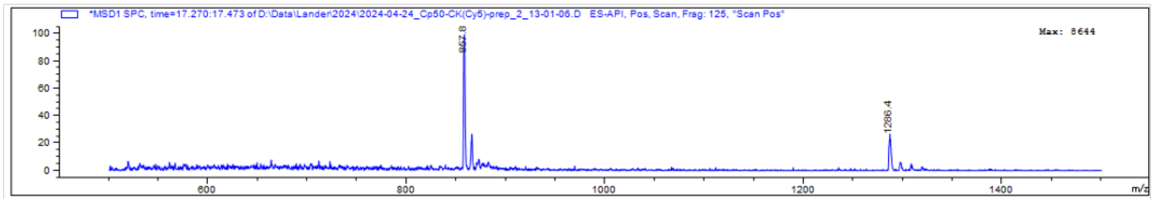

Figure S71. Chemical structure, calculated masses, HPLC chromatograms (214 and 650 nm) and ESI+ mass spectrum of isolated **Cp60 K14(sulfo-Cyanine5)**. Analytical HPLC purity: >98%, ESI+ MS (m/z): calculated 857.4 $[M+3H]^{3+}$, 1285.6 $[M+2H]^{2+}$, observed 857.8 $[M+3H]^{3+}$, 1286.4 $[M+2H]^{2+}$.

Cp60-K(Biotin)

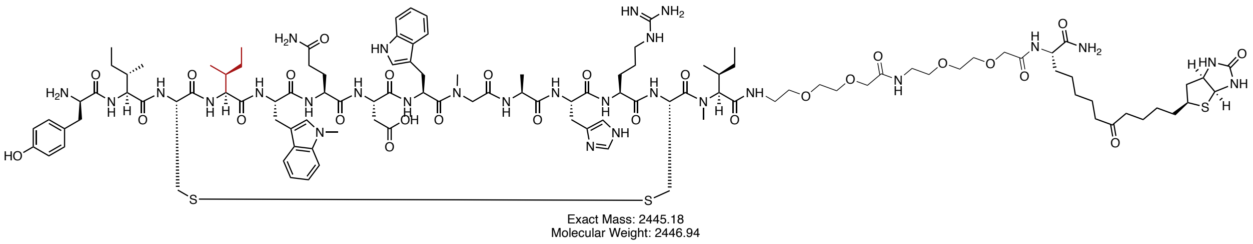

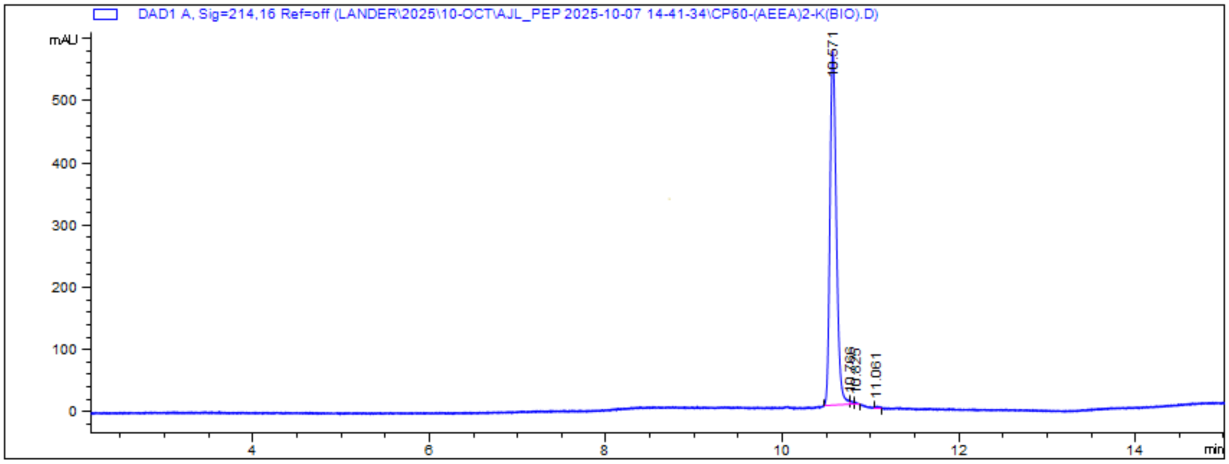

Figure S72. Chemical structure, calculated masses, HPLC chromatogram (214 nm) of isolated **Cp60-AEEA2-K(Biotin)**. Analytical HPLC purity: >95%.

Cp60-KK

Acetate salt used for preliminary *in vivo* pharmacokinetic analysis

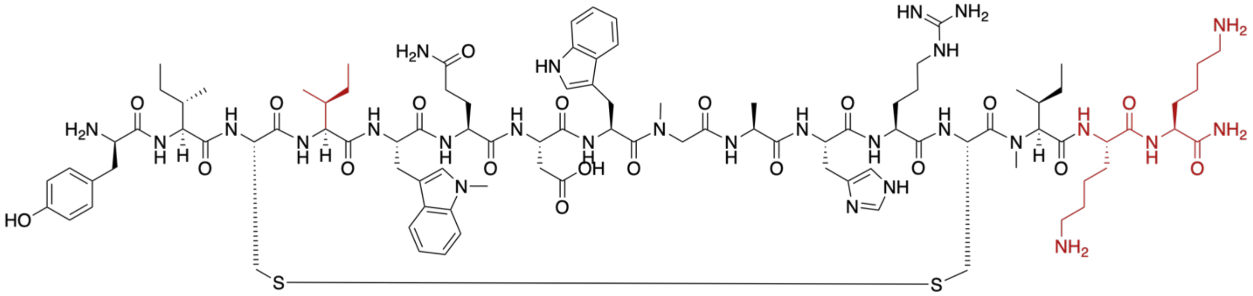

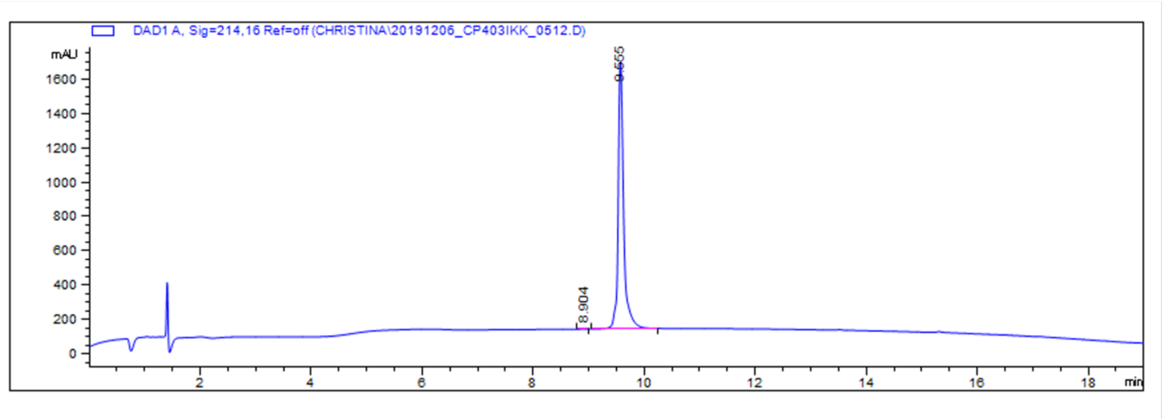

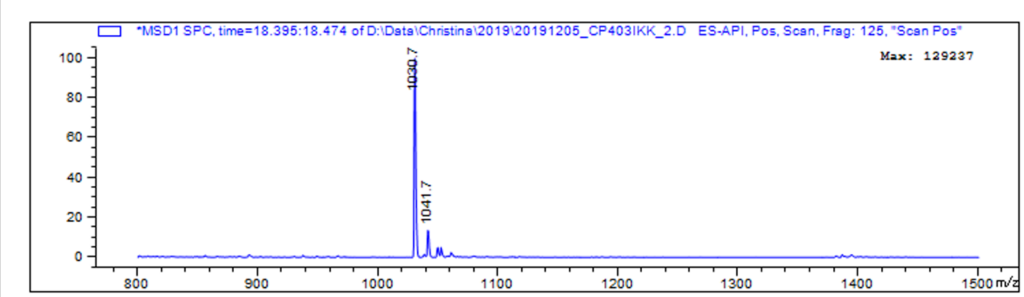

Figure S73. Chemical structure, calculated masses, HPLC chromatogram (214 nm) and ESI+ mass spectrum of isolated **Cp60-KK**. Analytical HPLC purity: >98%, ESI+ MS (m/z): calculated 1030.0 $[M+2H]^{2+}$, 1041.0 $[M+H+Na]^{2+}$, observed 1030.7 $[M+2H]^{2+}$, 1041.7 $[M+H+Na]^{2+}$.
